# Design, synthesis, X-ray crystal structures, anticancer, DNA binding, and molecular modelling studies of pyrazole–pyrazoline hybrid derivatives†

**DOI:** 10.1039/d3ra04873j

**Published:** 2023-09-06

**Authors:** Manish Rana, Hungharla Hungyo, Palak Parashar, Shaban Ahmad, Rabiya Mehandi, Vibha Tandon, Khalid Raza, Mohammed A. Assiri, Tarik E. Ali, Zeinhom M. El-Bahy, Rahisuddin

**Affiliations:** a Molecular and Biophysical Research Lab (MBRL), Department of Chemistry, Jamia Millia Islamia New Delhi 110025 India rahisuddin@jmi.ac.in +91 9871460479; b Department of Chemistry, Ramjas College, University of Delhi Delhi 110007 India; c Special Centre for Molecular Medicine, Jawaharlal Nehru University New Delhi 110067 India; d Department of Computer Science, Jamia Millia Islamia New Delhi 110025 India; e Department of Chemistry, Faculty of Science, King Khalid University Abha 61421 Saudi Arabia; f Department of Chemistry, Faculty of Science, Al-Azhar University Nasr City 11884 Cairo Egypt

## Abstract

We have designed and synthesized three pyrazole analogs (4, 5a, 5b), pyrazole-based chalcones (6a–6d) and (8a–8h), and *N*-formyl/acetyl 1,3,5-trisubstituted pyrazoline analogs (7a–7d), (9a–9d). FT-IR, ^1^H, ^13^C NMR, and mass spectrometry techniques were used to describe the structures of all the synthesized analogs. The single crystal X-ray method was used to identify the molecular structure of derivatives 4 and 5a. All synthesized analogs were screened by MTT assay on two cancer cell lines, the human lung cancer cell line (A549) and cervical cancer cell line (HeLa). Among all compounds, analog 9d demonstrates significant anticancer activity against HeLa (IC_50_ = 23.6 μM) and A549 (IC_50_ = 37.59 μM). The non-interactive interaction of active compound (9d) with Calf thymus DNA (Ct-DNA) has been investigated through various methods, such as UV-vis absorption, emission, cyclic voltammetry and circular dichroism. The DPPH (2,2-diphenyl-1-picrylhydrazyl) free radical has been used to measure the antioxidant capacity of the pyrazoline derivative (9d). The outcomes showed that active analog has significant antioxidant activity. In addition, MD simulation of the EGFR tyrosine kinase protein–ligand complex was performed at a time scale of 100 ns. The MMGBSA data of ligand–protein complex are showed stable interactions up to 100 ns.

## Abstract 2

We have designed and synthesized three pyrazole analogs (4, 5a, 5b), pyrazole-based chalcones (6a–6d) and (8a–8h), and *N*-formyl/acetyl 1,3,5-trisubstituted pyrazole analogs (7a–7d), (9a–9d) *via* Suzuki cross-coupling as the key step.

## Graphical abstract

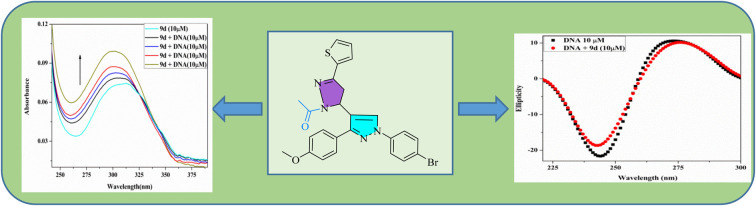

## 1. Introduction

Cancer is a significant health issue in both developed and developing countries since it is the second most feared illness in the world that causes death after cardiovascular diseases.^1–3^ Heterocyclic compounds have exceptional medicinal utility due to their unique action and are utilised in treating serious infectious disorders. A broad spectrum of these heterocycles has been investigated to produce therapeutically important compounds including chalcone and its derivatives, such as pyrazoles.^4,5^ Commonly referred to as chalcones, α–β unsaturated ketones are a significant family of natural products as well as synthetic chemicals that have shown a variety of biological activity.^6,7^ However, not many ways for derivatizing flavonoids, particularly chalcones, under Suzuki cross-coupling conditions have been reported.^8–10^ As parent heterocycles of several commercially available medications, pyrazoline and pyrazole heterocycles have unique biological properties.^11,12^ Pyrazole dihydropyrazole is an active heterocycle that was initially discovered in watermelon seeds and is utilised as an antibacterial,^13^ antitubercular,^14^ antiviral,^15^ anti-malarial,^16^ anti-cancer,^17,18^ analgesic,^19^ and anti-inflammatory drug.^20,21^ In the framework of the hybrid pharmacophore method, which enables the achievement of new pharmacological profiles, potentiation of action, and decrease in toxicity, it is noteworthy that combining pyrazole fragments with other heterocycles is a recognised strategy to construct drug-like compounds. A viable direction for contemporary medicinal chemistry in this situation is the synthesis of novel drug-like compounds based on the pharmacologically appealing scaffolds of pyrazole/pyrazoline.^22^ Pyrazole and pyrazoline, working together synergistically, have produced compounds with enhanced anticancer activity.^23^ Here, we attempted to develop a variety of heterocyclic derivatives with potential anticancer action against human lung cancer cells (A549) and cervical cell line (HeLa). The current research investigates the properties of heterocyclic derivatives in terms of MD simulation, DNA-binding, antioxidant, and ADMET assay. The design strategy for synthesized compounds is:
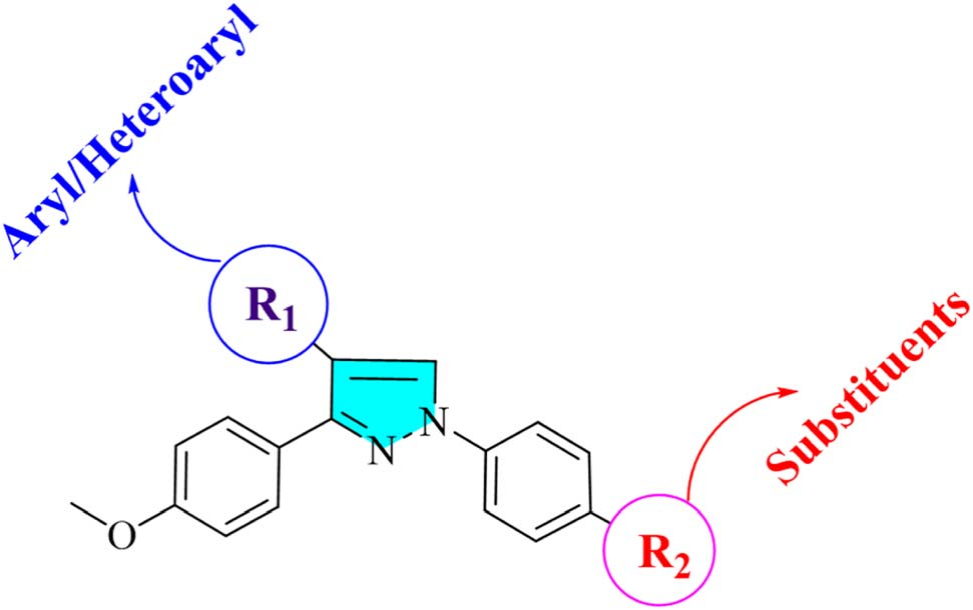


## 2. Result and discussion

### 2.1 Chemistry

**Fig. 1:**
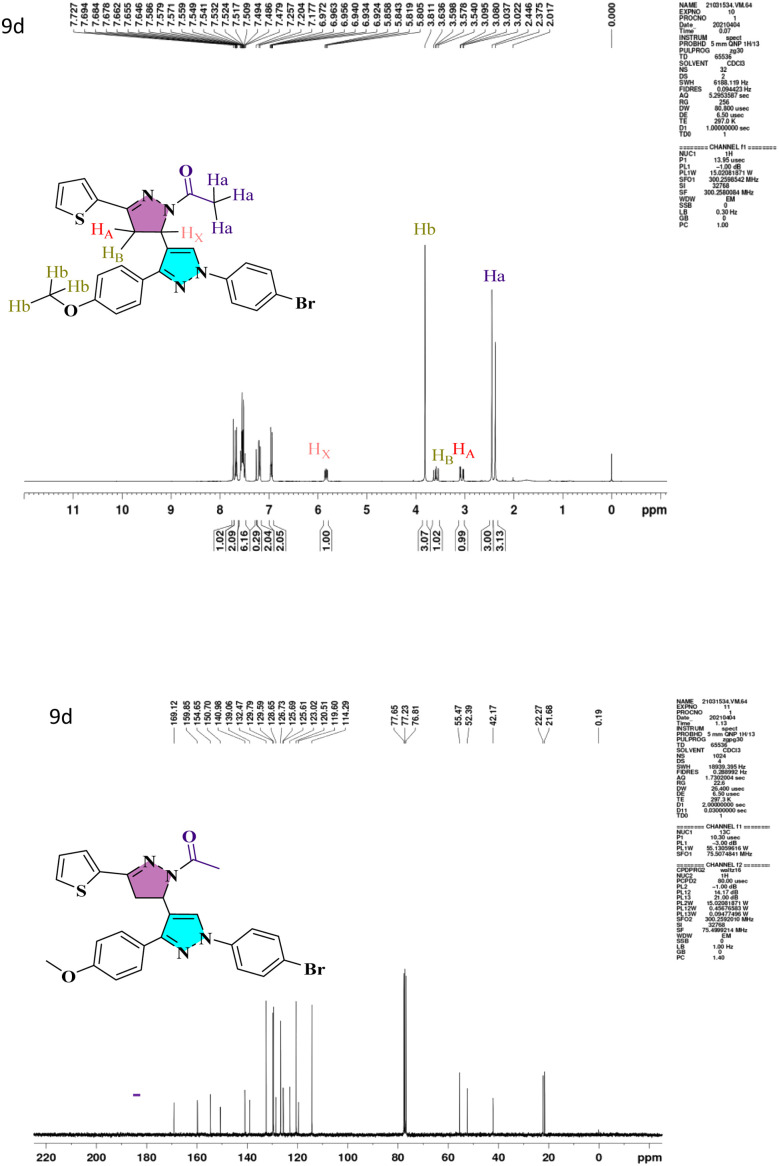
^1^H and ^13^C NMR spectra of lead compound 9d.

The intermediate 3 was synthesized by the condensation reaction of 4-bromo phenyl hydrazine and 4-methoxy acetophenone. To obtain pyrazole derivative 4, the intermediate 3 was cyclized with formylation by the Vilsmeier–Haack reaction. The chalcone derivatives 6a–6d were synthesized by the condensation reaction of pyrazole analog 4 with substituted acetophenone in the presence of 50% NaOH at room temperature. The derivatives 6a–6d further react with hydrazine hydrate in the presence of formic/acetic acid to obtain 7a–7d and 9a–9d, respectively. Analogs 4 and 6a–6d react with substituted boronic acid using Suzuki coupling reaction in the presence of Pd(PPh_3_)_4_ catalyst in solvent ratio 1 : 1 : 1 (H_2_O : THF : Toluene). The IR spectra of compound 4 showed a conspicuous, strong absorption band at 1678 cm^−1^ caused by the presence of the –C=O group in carbaldehyde. At 3130 cm^−1^, a second sharp, strong absorption band attributed to the C–H stretching of the carbonyl group occurred. Compound 6a–6d has a –C=O peak range of 1648–1663 cm^−1^ and a –C=C– range of 1529–1596 cm^−1^. The IR spectra of the pyrazolo-pyrazoline derivatives (7a–7d) and (9a–9d) show distinctive (C=O), (C=N) bands that emerge at 1663–1719 and 1592–1663 cm^−1^, respectively. The characteristic peaks of heterocyclic analogs are given in Fig. S1.† A conventional ABX system, in which methylene proton resonates as a pair of doublets seen at the range 3.05–3.27 ppm (*H*_A_) and 3.58–3.78 ppm (*H*_B_) for compounds 7a–7d and 9a–9d respectively, indicated the development of pyrazoline ring in the target compounds. Due to its vicinal interaction with the magnetically non-equivalent proton of the methylene (–CH_2_) group, methine (–CH) proton was observed as a doublet of doublets at 5.78–5.85 ppm (*H*_X_) and a singlet of methoxy proton in the range of 3.80–3.91 ppm. In ^13^C NMR, compounds 4, 5a, and 5b show peaks in the range 185.09–185.27 ppm belonging to –CHO groups, confirming the formylation of the pyrazol ring. The compounds 6a–6d and 8a–8h show –C=O groups at 181.40–191.14 ppm. The compounds 7a–7d exhibited an *N*-formyl peak in the range of 160.31–160.44 ppm and an *N*-acetyl peak for compounds 9a–9d in the range of 169.12–176.55 ppm. All the compounds show –OMe peak in the range of 55.26–55.61 ppm. The –CH protons of the pyrazoline ring for compounds 7a–7d and 9a–9d are in the range of 51.51–52.72 ppm, and –CH_2_ protons are at 42.06–44.90 ppm, which confirms the formation of pyrazoline ring. The ^1^H NMR and ^13^C NMR spectra of lead compound 9d are shown in Fig. 1 and remaining in Fig. S1.† The FT-IR and mass spectra of all heterocyclic analogs are given in Fig. S2 and S3.†

### 2.2 Crystal structures

**Fig. 2:**
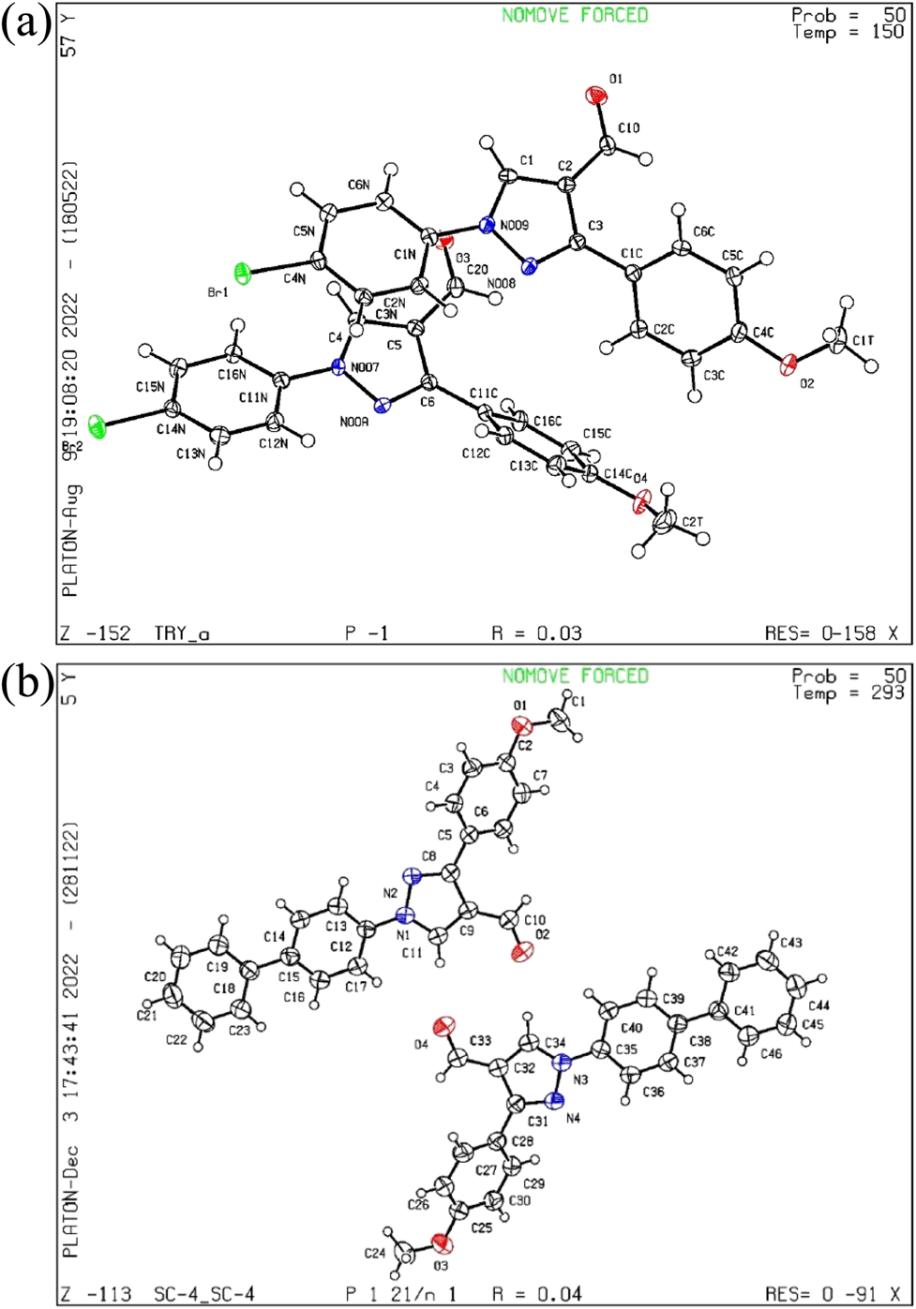
(a) The ORTEP view of compound 4, (b) the ORTEP view of compound 5a.

The pyrazol derivative structure and bonding characteristics were determined using X-ray crystallography.^24^ The X-ray crystal analyses demonstrate that the unit cell of compounds 4 and 5a was triclinic and monoclinic, respectively. Compound 4 has non-centrosymmetric space group *P*1̄ and 5a with *P*2_1_/*n* space group, and *z* = 4, *z* = 8 correspond to compounds 4 and 5a based on single X-ray crystal studies. Lattice parameters for 4, *a* = 9.348(2) Å, *b* = 9.793(2) Å, *c* = 16.366(4) Å, *α* = 87.493(6)°, *β* = 87.318(6)°, *γ* = 84.676(6)°, and for 5a, *a* = 21.54552(17) Å, *b* = 7.38135(7) Å, *c* = 22.77667(19) Å, *α* = 90°, *β* = 101.0921(8)°, *γ* = 90° unit cell, as shown in Tables 1 and 2. The CCDC codes for the crystalline compounds 4 and 5a are 2196195 and 2235766, respectively. The ORTEP view of 4 and 5a is shown in Fig. 2.

**Table 1:** Crystal data for compound 4

Identification code	4
Empirical formula	C_17_H_13_BrN_2_O_2_
Formula weight	357.19
Temperature/K	150(2) K
Crystal system	Triclinic
Space group	*P*1̄
*a*/Å	9.348(2) Å
*b*/Å	9.793(2) Å
*c*/Å	16.366(4) Å
*α*/°	87.493(6)°
*β*/°	87.318(6)°
*γ*/°	84.676(6)°
Volume/Å^3^	1489.0(6) Å^3^
*Z*	4
*ρ*_calc_mg m^−3^	1.593
*μ*/mm^−1^	2.769
*F*(000)	720.0
Crystal size/mm^3^	0.06 × 0.05 × 0.04
Radiation	MoKα (*λ* = 0.71073)
2*Θ* range for data collection/°	2.090 to 25.062°
Index ranges	−11 ≤ *h* ≤ 11, −11 ≤ *k* ≤ 11, −19 ≤ *l* ≤ 19
Reflections collected	35 618
Independent reflections	5267 [*R*(int) = 0.0586]
Data/restraints/parameters	5267/0/399
Goodness-of-fit on *F*^2^	1.024
Final *R* indexes [*I* ≥ 2*σ*(*I*)]	*R*_1_ = 0.0277, w*R*_2_ = 0.0717
Final *R* indexes [all data]	*R*_1_ = 0.0424, w*R*_2_ = 0.0781
Largest diff. peak/hole/e Å^−3^	0.349 and −0.480 e Å^−3^

**Table 2:** Crystal data and structure refinement for 5a

Identification code	5a
Empirical formula	C_23_H_18_N_2_O_2_
Formula weight	354.41
Temperature/K	293(2)
Crystal system	Monoclinic
Space group	*P*2_1_/*n*
*a*/Å	21.54552(17)
*b*/Å	7.38135(7)
*c*/Å	22.77667(19)
*α*/°	90
*β*/°	101.0921(8)
*γ*/°	90
Volume/Å^3^	3554.62(5)
*Z*	8
*ρ*_calc_mg m^−3^	1.3244
*μ*/mm^−1^	0.683
*F*(000)	1492.6
Crystal size/mm^3^	0.39 × 0.21 × 0.14
Radiation	Cu Kα (*λ* = 1.54184)
2*Θ* range for data collection/°	5.18 to 136.32
Index ranges	−23 ≤ *h* ≤ 25, −8 ≤ *k* ≤ 8, −27 ≤ *l* ≤ 21
Reflections collected	36 401
Independent reflections	6446 [*R*_int_ = 0.0294, *R*_sigma_ = 0.0161]
Data/restraints/parameters	6446/0/490
Goodness-of-fit on *F*^2^	1.032
Final *R* indexes [*I* ≥ 2*σ*(*I*)]	*R*_1_ = 0.0368, w*R*_2_ = 0.0989
Final *R* indexes [all data]	*R*_1_ = 0.0426, w*R*_2_ = 0.1031
Largest diff. peak/hole/e Å^−3^	0.18/−0.14

### 2.3 Cytotoxicity

**Fig. 3:**
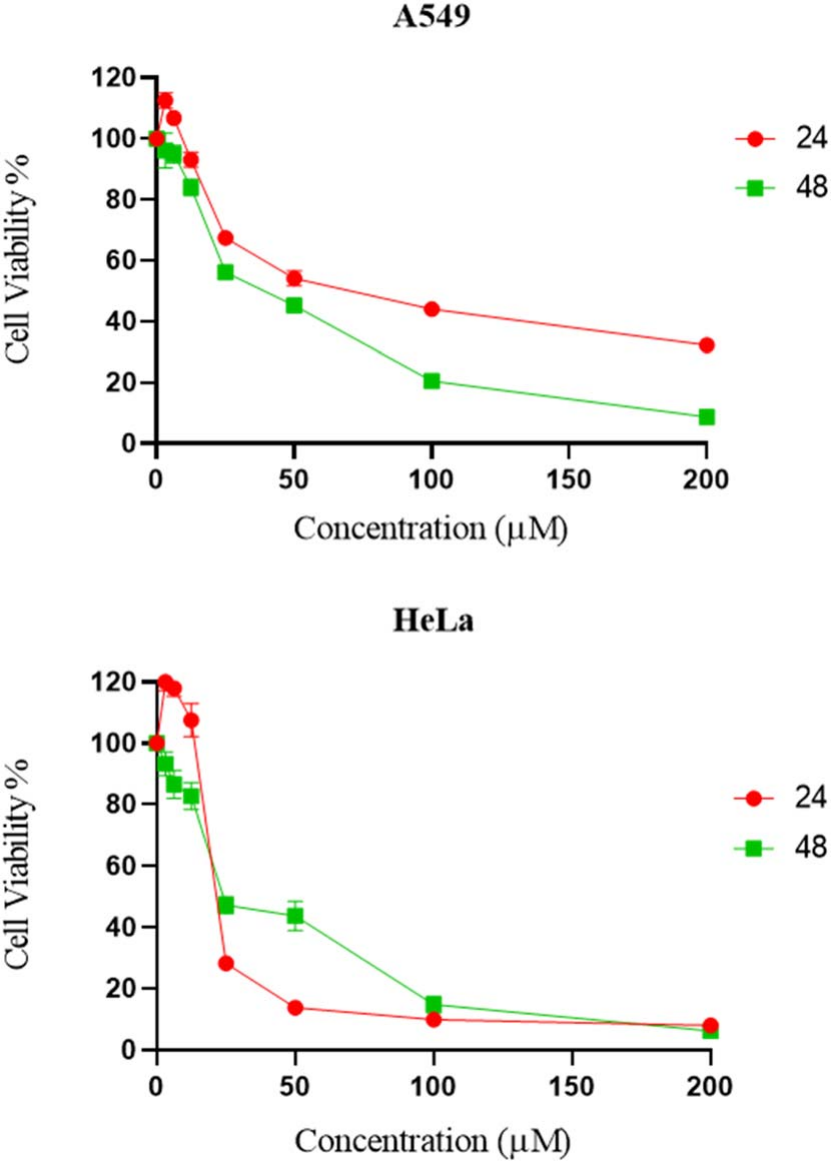
Dose response curves of compound 9d against A549 and HeLa cancer cell line.

The cytotoxicity of synthesized compounds was evaluated against two cancer cell lines (A549 and HeLa) *via* MTT assay.^25^ It was found that the majority of chemical compounds synthesized in the laboratory showed no significant inhibitory activity. Compound 9d showed significant bioactivity as compared to standard drug nocodazole.^26^ As shown in Fig. 3 and Table 3, compound 9d exhibited significant inhibitory activities against HeLa and A549 cells at a dose concentration of 23.6 μM and 37.59 μM at 48 h, respectively.

**Table 3:** *In vitro* cytotoxicity of heterocyclic derivatives 4, (5a, 5b) (6a–6d), (7a–7d), (8a–8h) and (9a–9d) against human cancer cell lines (A549 and HeLa) in terms of IC_50_ value in μM

Compounds	A549	HeLa
4	96.88	109.67
5a	46.25	>500
5b	54.06	186.93
6a	>500	>500
6b	>500	>500
6c	>500	>500
6d	>500	>500
7a	>500	>500
7b	>500	>500
7c	>500	>500
7d	29.40	41.48
8a	>500	>500
8b	>500	>500
8c	>500	>500
8d	>500	>500
8e	>500	>500
8f	>500	>500
8g	>500	>500
8h	>500	>500
9a	179.82	>500
9b	124.03	166
9c	>500	>500
9d	**23.60**	**37.59**
Nocodazole^26^	1.87 ± 0.5	2.83 ± 0.3

### 2.4 Molecular docking and MD simulation

#### 2.4.1 Interaction analysis and ADMET analysis

**Fig. 4:**
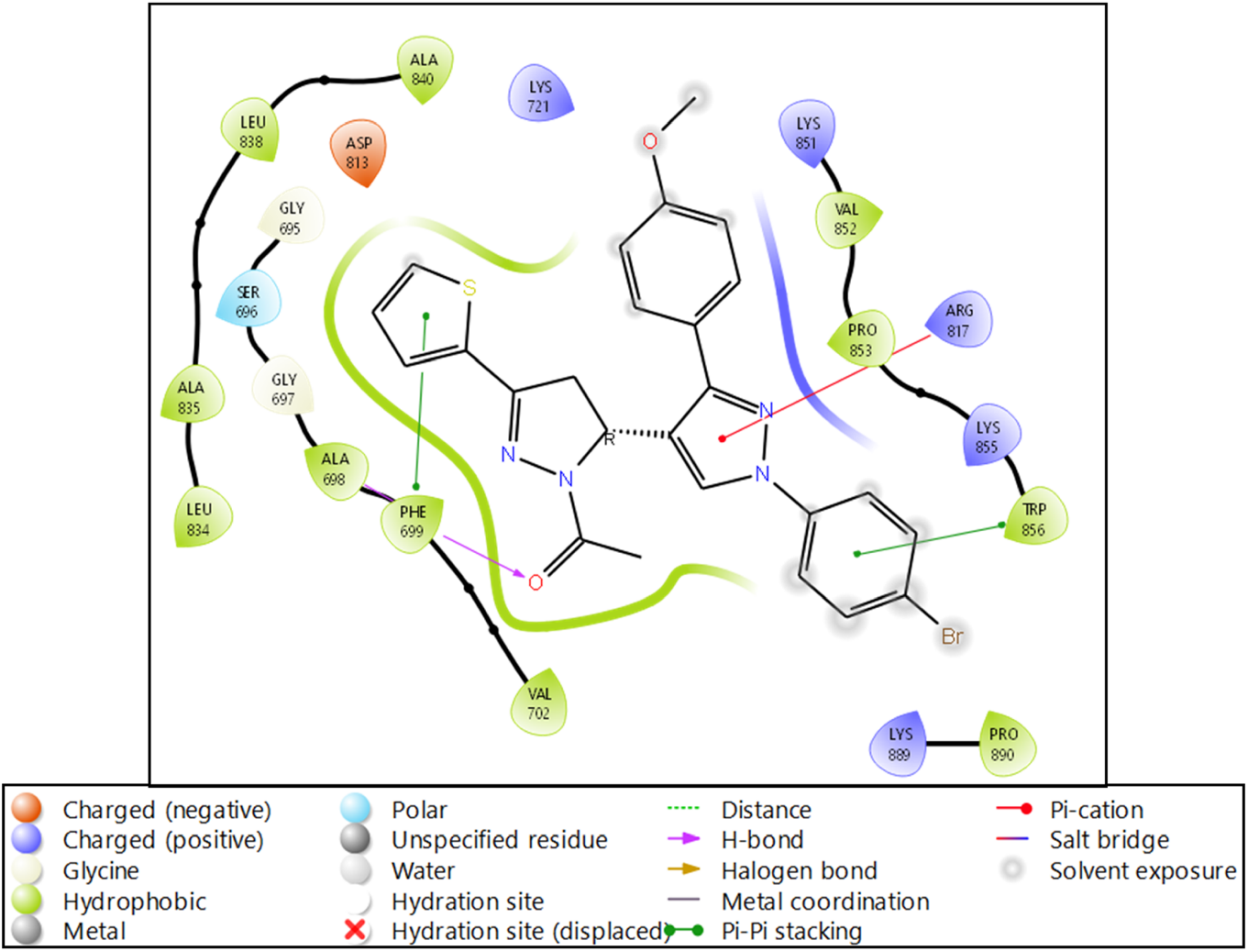
Ligand interaction diagram of EGFR tyrosine kinase with 9d and the legend is shown to understand the residues and interaction types.

The molecular docking of 4HJO-9d produced a docking score of −4.32 kcal mol^−1^ and an MM\GBSA score of −43.65 kcal mol^−1^, which are considered good scores (Table 4). 9d interacts with the protein's residues while forming four bonds.^27^ PHE699 formed one hydrogen bond with the O atom and one pi–pi stacking with the benzene ring. TRP856 also formed one pi–pi stacking with the benzene ring, while ARG817 formed the pi–cation interaction with another ring with two nitrogen atoms (Fig. 4). The ADMET of the compound shows zero amines, amidine, acid, amide, rotor, and rtvFG and is inactive against the CNS. The compound has zero donors and five acceptor hydrogen bonding capacity with MW 521.43. The details are shown in Table 5.

**Table 4:** The docking score, MM\GBSA score, and other calculations obtained during molecular docking

PDB ID	PDB resolution	Docking score	MM/GBSA dG bind	Glide ligand efficiency sa	Glide ligand efficiency ln
4HJO	2.75	−4.32	−43.65	−0.42	−0.961

**Table 5:** ADMET properties of 9d computed with the QikProp tool against standard values along with the descriptors

Descriptors	Standard values	9d	Descriptors	Standard values	9d
#stars	0–5	2	QPlogS	−6.5–0.5	−8.967
#amine	0–1	0	CIQPlogS	−6.5–0.5	−9.2
#amidine	0	0	QPlogHERG	Concern below −5	−7.252
#acid	0–1	0	QPPCaco	<25 poor, >500 great	3109.509
#amide	0–1	0	QPlogBB	−3.0–1.2	0.203
#rotor	0–15	1	QPPMDCK	<25 poor, >500 great	7799.249
#rtvFG	0–2	0	QPlogKp	−8.0 to −1.0	−0.938
CNS	−2 (inactive), +2 (active)	1	IP(eV)	7.9–10.5	8.744
mol MW	130.0–725.0	521.43	EA(eV)	−0.9–1.7	1.123
Dipole	1.0–12.5	7.054	#metab	1–8	4
SASA	300.0–1000.0	798.515	QPlogKhsa	−1.5–1.5	1.324
FOSA	0.0–750.0	208.69	HumanOralAbsorption	N/A	1
FISA	7.0–330.0	53.064	PercentHumanOralAbsorption	>80% is high, <25% is poor	100
PISA	0.0–450.0	415.337	SAfluorine	0.0–100.0	0
WPSA	0.0–175.0	121.424	SAamideO	0.0–35.0	0
Volume	500.0–2000.0	1430.482	PSA	7.0–200.0	64.693
donorHB	0.0–6.0	0	#NandO	2–15	6
accptHB	2.0–20.0	5.25	RuleOfFive	Maximum is 4	2
dip^2/V	0.0–0.13	0.0347858	RuleOfThree	Maximum is 3	1
ACxDN^.5/SA	0.0–0.05	0	#ringatoms	N/A	27
glob	0.75–0.95	0.7689006	#in34	N/A	0
QPpolrz	13.0–70.0	54.418	#in56	N/A	27
QPlogPC16	4.0–18.0	15.866	#noncon	N/A	2
QPlogPoct	8.0–35.0	21.98	#nonHatm	N/A	33
QPlogPw	4.0–45.0	9.82	Jm	N/A	0
QPlogPo/w	−2.0–6.5	6.653			

#### 2.4.2 Molecular dynamics simulations

MD simulation is a technique used to study the physical movements of atoms and molecules. It involves using computational algorithms to solve the equations of motion for a system of particles based on the interactions between them and external forces. In an MD simulation, the system is represented as a collection of atoms or molecules treated as point masses. The interactions between these particles are modelled using various potential energy functions, which describe the forces between the particles. The equations of motion for the system are then solved using numerical techniques, such as the Verlet algorithm or the Leapfrog algorithm. The result is a series of positions and velocities for each particle at discrete time steps, which can be used to calculate various properties of the system, such as temperature, pressure, or energy. MD simulation is very computationally intensive and requires significant amounts of computer power. However, advances in computing technology have made it possible to perform large-scale MD simulations on systems containing millions of atoms or molecules. This has led to a wide range of important scientific discoveries and practical applications, such as developing new materials and drugs and understanding biological processes at the molecular level. The molecular dynamics simulation was performed for 100 ns to understand the protein and ligand stability together and individually and analysed the root mean square deviation (RMSD), root mean square fluctuations (RMSF) and intermolecular interaction to understand which residues interact with the other molecules and ligand 9d.^28,29^

#### 2.4.3 RMSD and RMSF analysis

**Fig. 5:**
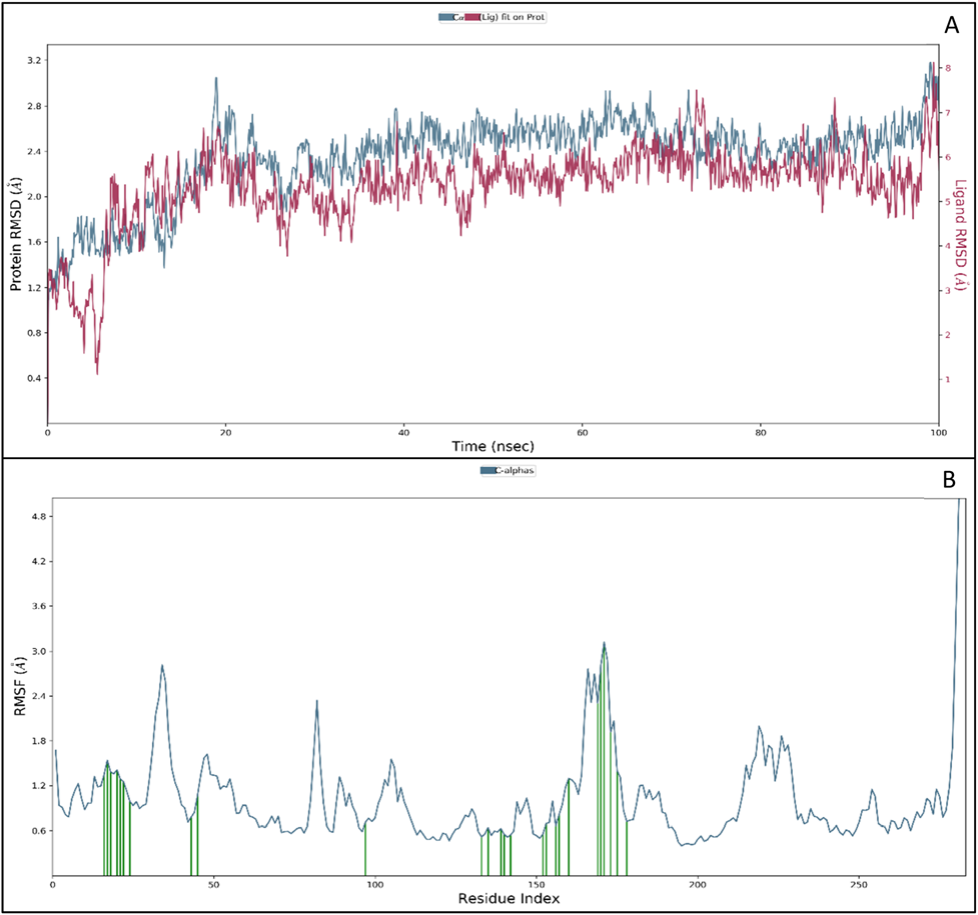
(A) Root Mean Square Deviation (RMSD) of protein (blue) and ligand (red) in Angstroms (Å) against time and (B) Root Mean Square Fluctuations (RMSF) of protein (Å) (blue) and ligand contact with the amino acids (green) against residue index.

RMSD measures the difference between two sets of values. It is often used in comparing the results of a simulation to experimental data or to compare the results of different simulations. It measures the overall deviation between the two sets of values and is often used to assess the accuracy or precision of a simulation or other computational models. It is a commonly used measure in chemistry, biology, and materials science, where it is often used to compare the results of a simulation to experimental data or the results of different simulations. The protein and ligand structures deviated initially, and in 1.50 ns, the protein deviated 1.34 (Å) while the ligand deviated 3.73 (Å) from zero and kept deviating till 20 ns with a deviation of protein at 2.75 (Å) and for ligand at 6.29 (Å). After the initial 20 ns, the protein showed an utterly stable performance with minor deviations, and at 100 ns, the protein exhibits 3.05 (Å) and 6.80 (Å) for the ligand (Fig. 5A). The cumulative deviation after excluding the initial deviation was 0.3 (Å) for protein. For the ligand, it was 0.51 (Å), which is considered best for the biological molecules, and the initial deviation was the result of an initial head change in the solute system, addition of ions, and application of forcefields.^30^

The RMSF measures the deviation of the positions of atoms or residues in a protein or other biological molecules from their average positions.^31^ It is often used in molecular dynamics (MD) simulations, where it can provide insight into the flexibility or stability of a protein or other biomolecules. The RMSF can be used to identify regions of a protein that are more flexible or less flexible, which can provide insight into the protein's function. It can also be used to compare the flexibility of different proteins or the results of different MD simulations. The RMSF is a valuable tool for understanding the structural and dynamic properties of proteins and other biological molecules at the atomic level. The protein did not show many fluctuations; however, a few residues, such as GLU710-LYS713, SER760, LYS843-GLY850, and GLN958-ASP960, show more fluctuation beyond 2 (Å). The fluctuated residues were not part of the interactions, so they do not bother much with the overall stability of the protein–ligand complex. There are many residues to which the ligand 9d formed the interactions. The interacting residues are LUE694, GLY695, SER696, ALA698PHE699, GLY700, VAL702, LYS721, LEU723, LEU775, HIS811, ASP813, ARG817, ASN818, LEU820, THR830, ASP831, LEU834, ALA835, LEU838, ALA847, GLU848, GLY849, LYS851, PRO853, and TRP856 (Fig. 5B).

#### 2.4.4 Intermolecular interactions

**Fig. 6:**
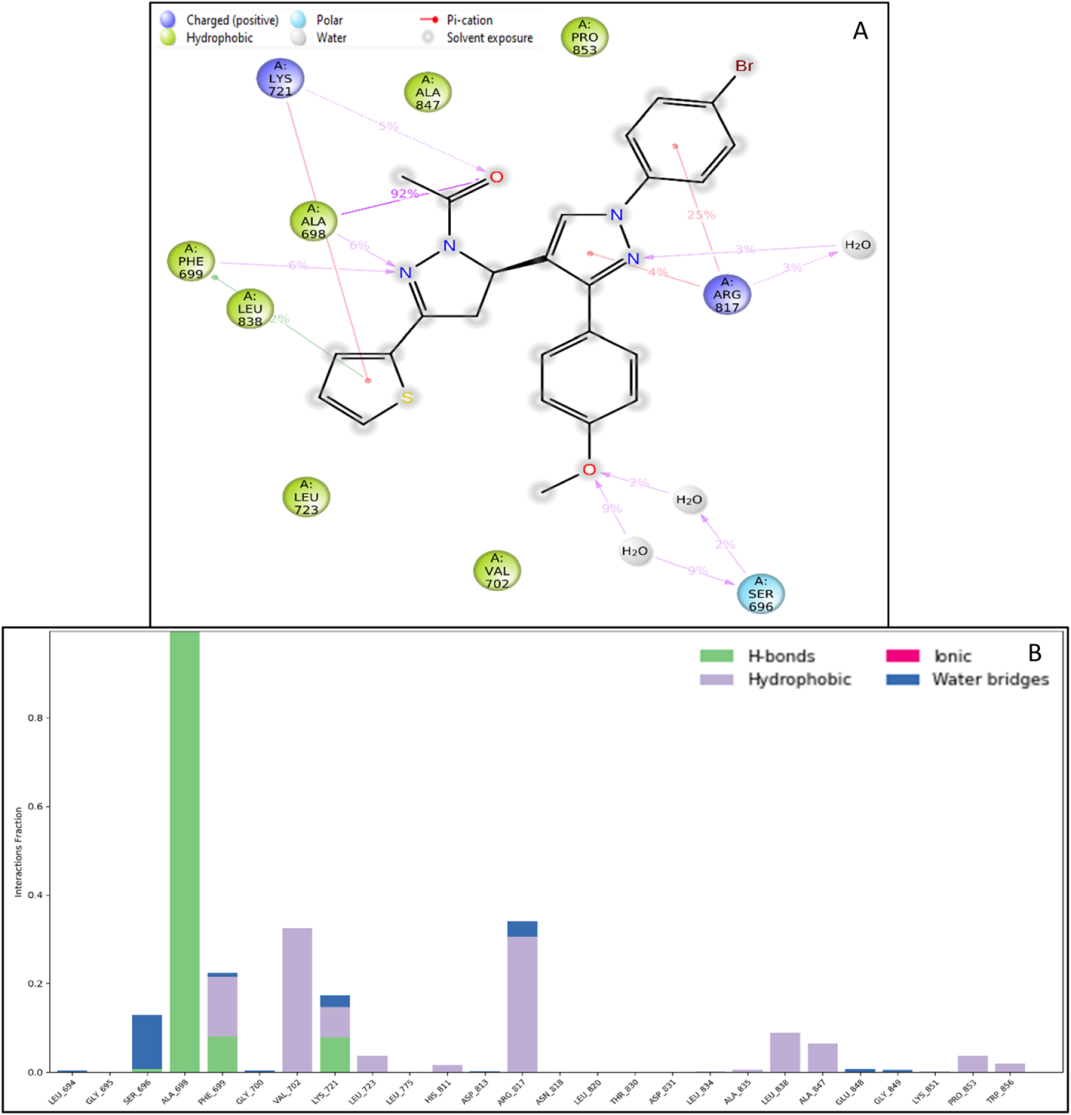
Intermolecular interactions formed during the molecular dynamics simulation (A) simulation interaction diagram and (B) histogram representation of the count of interactions and the legend is provided to understand each residue and bond type.

Intermolecular interactions are the forces that exist between molecules and that govern the behaviour of matter at the molecular level. These interactions play a crucial role in determining the properties of materials, such as their solubility and their physical and chemical behaviour. Several intermolecular interactions include van der Waals forces, hydrogen bonding, and coulombic interactions. These are studied using molecular dynamics simulations and quantum chemical calculations, which allow us to understand the behaviour of molecules at the atomic level and predict the properties of materials. For the complete 100 ns, we have extracted the interaction among the protein and ligand, including water molecules that participated in the water bridge. ALA698, PHE699, and LYS721 formed hydrogen bonds; PHE699 and HIS811 formed pi–pi stacking. LYS721 and ARG817 formed two pi-cations each, while SER696 and LYS721 formed water bridges (Fig. 6A). Further, the histogram representation in Fig. 6B shows the count of interactions.

### 2.5 DNA binding study

#### 2.5.1 Absorption titration

**Fig. 7:**
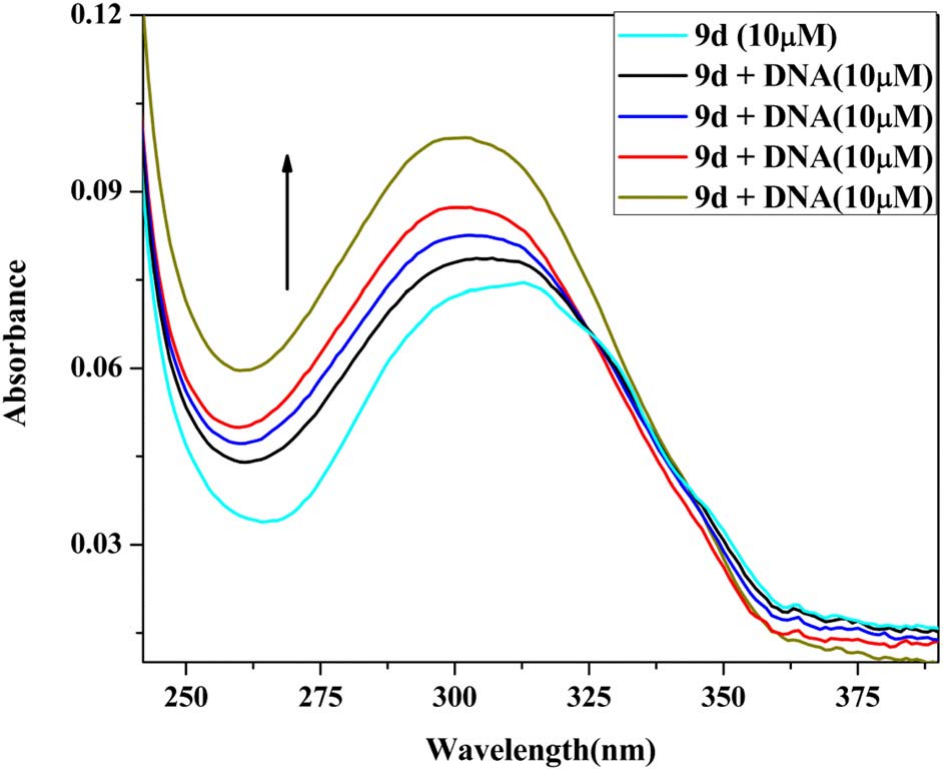
UV absorption spectra of 9d (10 μM) with increasing amounts of Ct-DNA (10–40 μM).

**Fig. 8:**
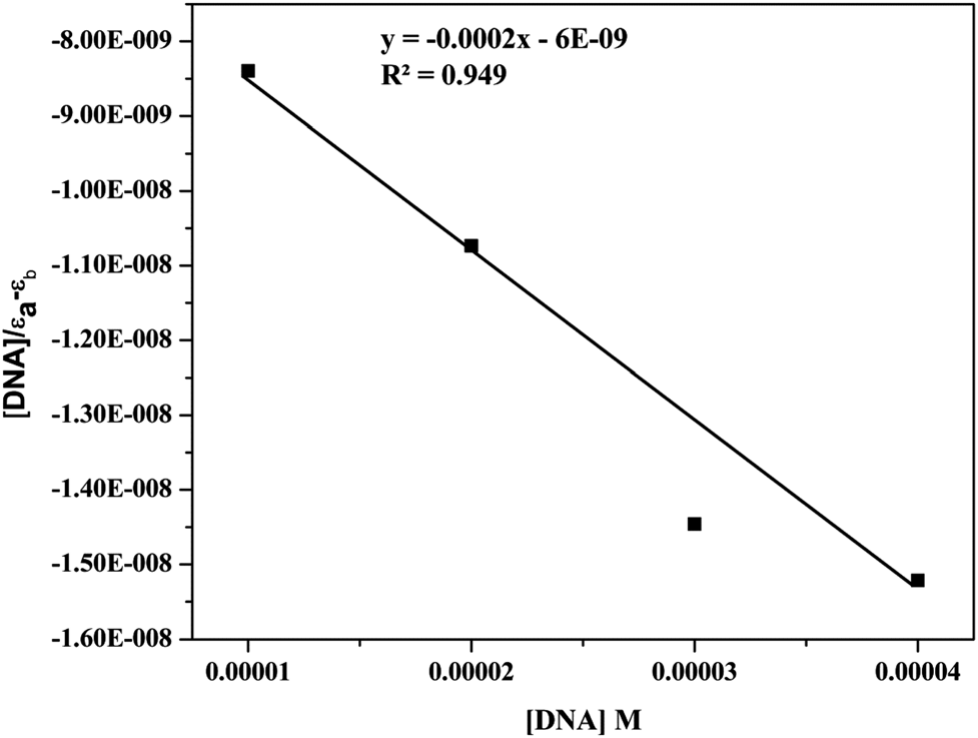
UV spectra and inset plot between [DNA]/[*ε*_a_ − *ε*_f_] *vs.* [DNA] of 9d (10 μM) with increasing amounts of Ct-DNA (10–40 μM).

By using UV-visible spectroscopic titration of the active compound (9d) with calf thymus CT-DNA, the manner and strength of DNA binding of the compound were investigated.^32,33^ Four different concentrations of CT-DNA (10–40 μM) were added slowly to a constant concentration (10 μM) of compound 9d. When CT-DNA is gradually added to compound 9d, the compound exhibits hyperchromism with a slight blue shift (Fig. 7). The extent of wavelength shift and hyperchromism in a molecule affects its ability to bind to DNA, and this ability typically correlates with the efficiency of the electrostatic and groove binding. The spectrum shifts observed in this study strongly indicated that the compound adhered to the CT-DNA *via* the electrostatic mode of binding. The intrinsic binding constant (*K*_b_) was calculated using the equation:[DNA]/(*ε*_a_ − *ε*_f_) = [DNA]/(*ε*_b_ − *ε*_f_) + 1/*K*_b_(*ε*_b_ − *ε*_f_)where *ε*_a_ represents the extinction coefficient for *A*(observed)/[complex], *ε*_f_ represents the extinction coefficient for pure compound, and *ε*_b_ represents the extinction coefficient for the compound in fully bound form. When the compound data was fitted into the aforementioned equation, it produced a straight line with a slope of 1/(*ε*_b_ − *ε*_f_) and a *y*-intercept of 1/*K*_b_(*ε*_b_ − *ε*_f_), and the value of *K*_b_ was calculated from the slope to intercept ratio (Fig. 8), found to be 3.6 × 10^4^.

#### 2.5.2 Emission titration

**Fig. 9:**
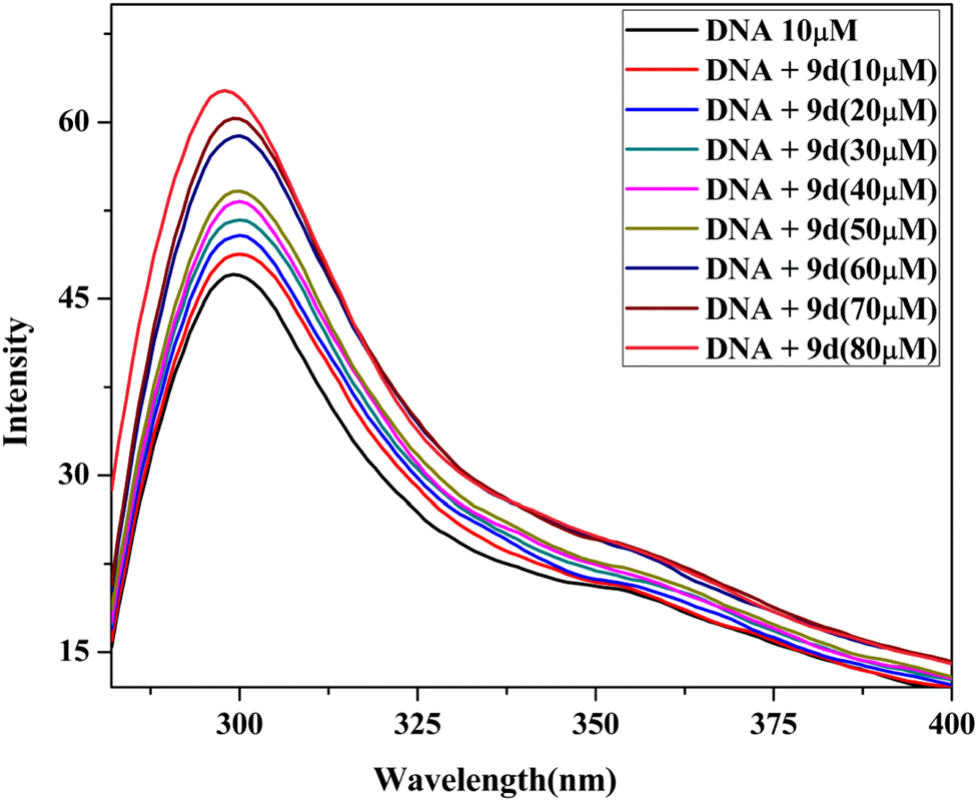
Emission titration of the lead compound 9d.

One of the most reliable and sensitive methods to ascertain how a molecule binds to DNA is fluorescence spectroscopy.^34,35^ When excited at a maximum wavelength of 260 nm in a buffer of 5 mM Tris–HCl/50 mM NaCl at room temperature, the fluorescence emission spectra of compound 9d showed an emission band in the 280–330 nm region. In both the presence and absence of CT DNA, compound fluorescence titration tests have been conducted (10–60 μM). The mode of a compound binding to CT DNA is determined by variations in wavelength shift and fluorescence intensity. As depicted in Fig. 9, the DNA shows an increase in the fluorescence intensity in the emission peak at 360 nm on continuous addition of increasing concentration of compound 9d to a constant volume of DNA. With no change in the position of the emission peak, the compound only exhibits hyperchromism. When a compound interacts electrostatically with the negatively charged phosphate backbone of DNA, the intensity of the emission increases, and there occurs a red/blue shift.

#### 2.5.3 Cyclic voltammetry

**Fig. 10:**
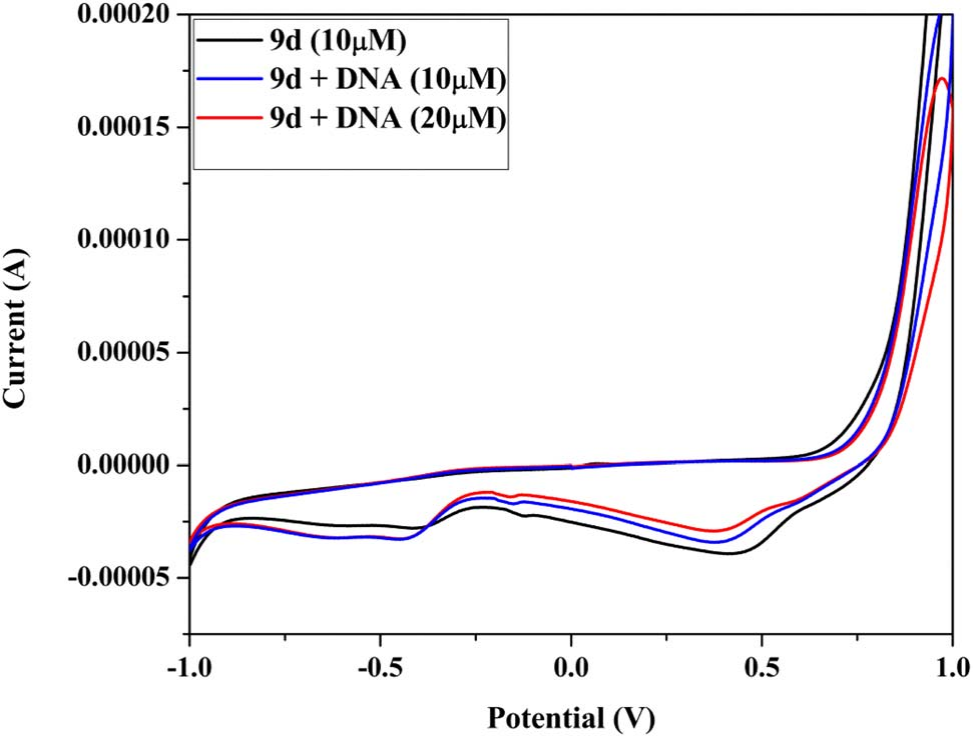
Cyclic voltammogram of analog 9d in Tris-buffer, at 100 mV s^−1^ scan rate with DNA (10–20 μM).

In order to understand the interaction between the biomolecule and the redox active molecule, electrochemical investigations can support the findings from other relevant biophysical methods.^36,37^ In an aqueous solution (50 mM NaCl/5 mM Tris–HCl buffer, pH 7.4), cyclic voltammogram of the compound 9d has been recorded in the absence and in the presence of Ct-DNA (Fig. 10). When a molecule binds with the DNA helix by electrostatic interaction, there will be a negative shift in the electrochemical potential value. If it binds to DNA by intercalation interaction, the shift is positive.^38^ The noticeable changes have been seen in the CV of the compound in the presence of DNA (Fig. 10). Peak potentials are significantly changed, and cathodic and anodic peak currents are reduced considerably, indicating some kind of interaction between the compound and DNA. The compound 9d may bind with DNA through electrostatic interaction because of the negative shift in the corresponding potential value.

#### 2.5.4 Circular dichroism

**Fig. 11:**
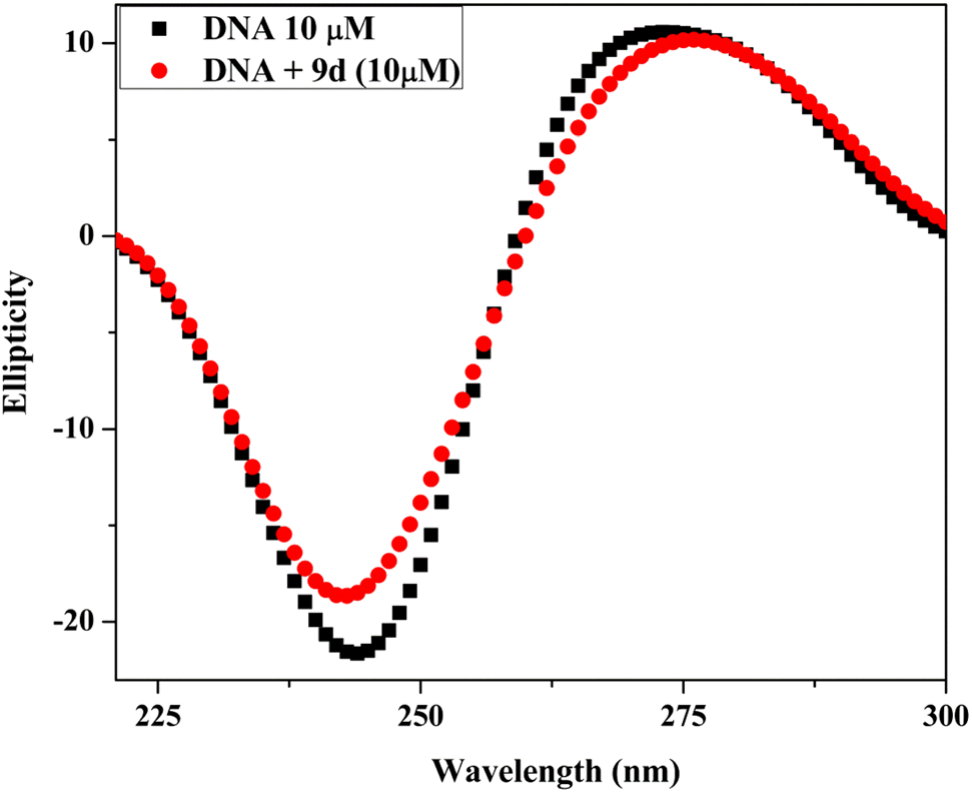
Circular dichroism spectra of Ct-DNA (10 μM) in the absence and presence of compound 9d (10 μM).

The respective addition of the compound to CT-DNA resulted in the recording of CD spectral alterations of CT-DNA. The CD spectra of CT-DNA after it was added to compound 9d are shown in Fig. 11. The right-handed B form of DNA has a positive band at 277 nm caused by base stacking and a negative band at 245 nm caused by helicity, as seen in the CD spectrum of calf thymus DNA. Intercalation increases the intensities of both bands, maintaining the right-handed B conformation of CT-DNA, while groove binding interactions between small molecules with DNA cause little to no changes in base stacking and helicity bands. Positive and negative band intensities in each of the two cases significantly decrease.^39,40^ These modifications support the groove-binding nature and suggest a non-intercalative manner of binding.

### 2.6 Antioxidant assay

**Fig. 12:**
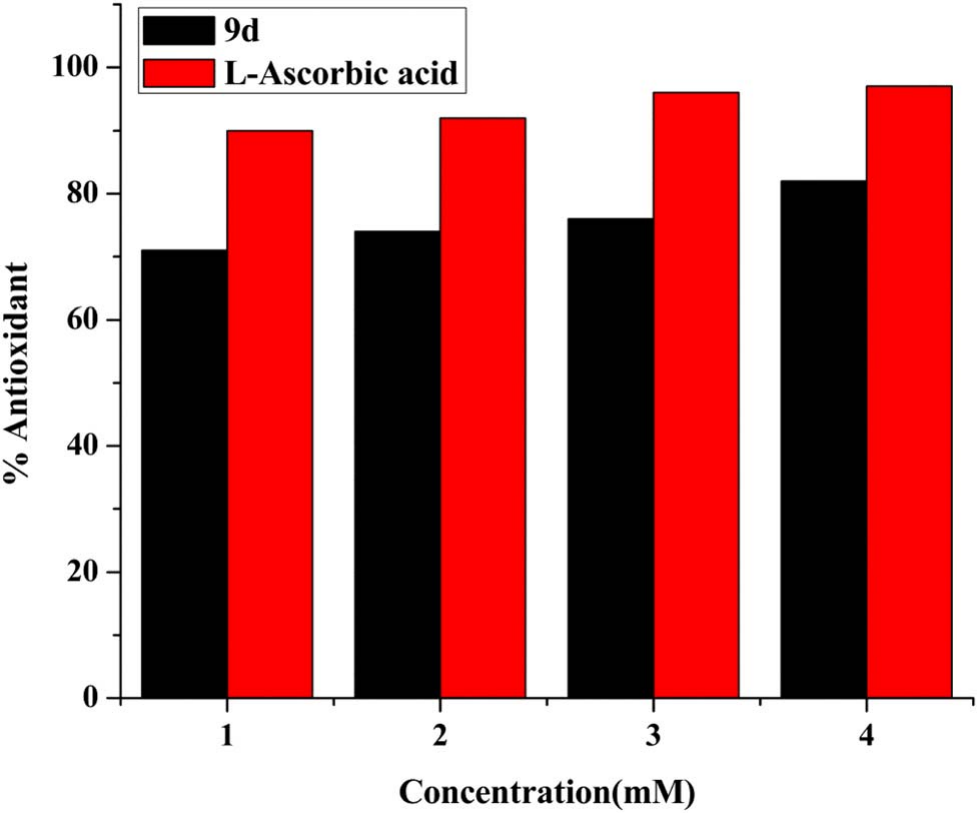
Concentration based bar graph of antioxidant ability of lead compound 9d.

DPPH (2,2 diphenyl-1-picrylhydrazyl hydrate) is a free radical species that is stable and tends to accept an electron or a hydrogen and is therefore reduced by an electron or hydrogen provided by an oxidant. The presence of the antioxidant moiety was confirmed after 1 h of incubation when the colour changed from purple to yellow, and the absorbance dropped around 516 nm. Calculating the IC_50_ value of the compound has been done by monitoring the antioxidant activity.^41,42^ Ascorbic acid has been taken as standard. The calculated IC_50_ of the compound was 1.37 ± 0.015 mg mL^−1^. The obtained results demonstrate that the antioxidant activity of the test compound rises with concentration, as seen in Fig. 12.

## 3. Experimental section

### 3.1 Material and methods

#### 3.1.1 General procedure for the synthesis of compound 3

The mixture of 4-bromo phenyl hydrazine (268.20 mg) and 4-methoxy acetophenone (150.18 mg) in methanol was refluxed at 80 °C for 4 h. TLC (Thin Layer Chromatography) was used to monitor the reaction progress. After the completion of the reaction, the mixture was cooled down, and a precipitate of hydrazone was obtained, filtered, and washed with methanol.

#### 3.1.2 General procedure for the synthesis of compound 4

POCl_3_ (2 mL) was added dropwise using a dropping funnel in DMF solvent (5 mL) at 0 °C for 30 min. After that, 319.20 mg solution of hydrazone (3) in DMF was added dropwise in Vilsmeier–Haack reagent, and then the reaction mixture was heated at 80 °C for 8 h, and the cool reaction mixture was poured in ice cold water and neutralized by the saturated solution of NaHCO_3_. The obtained precipitate was filtered off, washed with water, and recrystallized in ethanol solvent.

#### 3.1.3 General procedure for the synthesis of compounds 5a, 5b

The mixture of compound 4 (357.21 mg) and phenyl/4-methoxy phenyl boronic acid (121.93/151.96 mg) was refluxed at 100 °C for 4–12 h in solvent ratio 1 : 1 : 1 (H_2_O : THF : Toluene) at N_2_ as inert atmosphere in the presence of catalyst Pd(PPh_3_)_4_ (5 mol%). The progress of the reaction was monitored by TLC. After completion of the reaction, the reaction mixture is extracted with EtOAc (5 × 30 mL), the organic layer was dried with anhydrous Na_2_SO_4_ and concentrated under reduced pressure. The precipitates obtained were filtered, washed, dried and recrystallized in ethanol.

#### 3.1.4 General procedure for the synthesis of compounds 6a–6d

The chalcone derivatives (6a–6d) were synthesized by the condensation reaction of the pyrazole derivative (4) (357.21 mg) and substituted acetophenones stirred at r.t for 12 h in a solvent mixture of dioxane : ethanol (1 : 4) using 50% NaOH as a base. The precipitates obtained during the reaction were filtered, dried in a vacuum desiccator, and crystallized in ethanol.

#### 3.1.5 General procedure for the synthesis of compounds 7a–7d

**Scheme 1:**
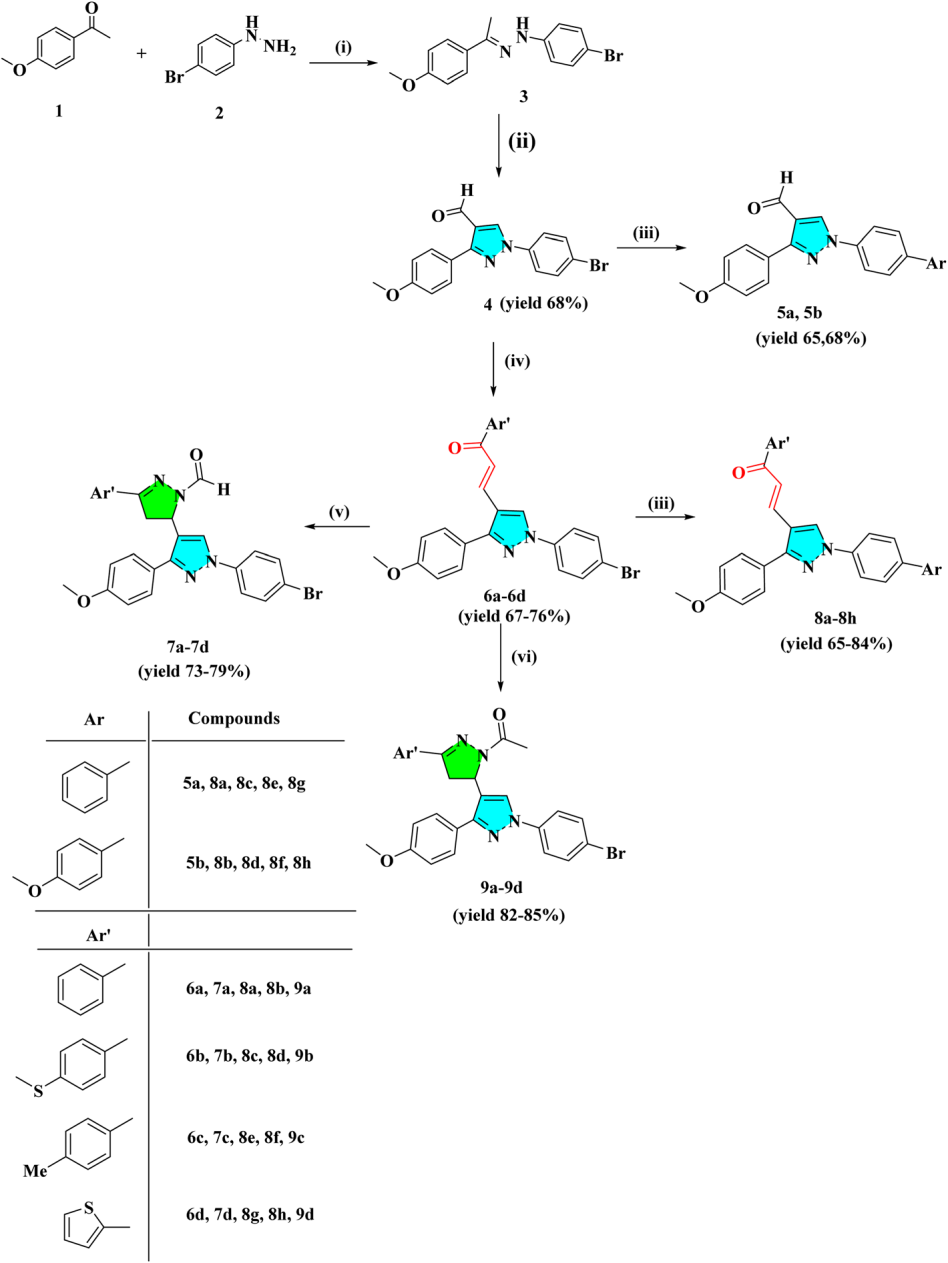
General procedure for the synthesis of heterocyclic derivatives. (i) Methanol, reflux for 4 h; (ii) DMF/POCl_3_ 0–80 °C, NaHCO_3_ (iii) Pd(PPh_3_)_4_ (5 mol%), Ar-B(OH)_2_, H_2_O : THF : Toluene (1 : 1 : 1) reflux for 4–12 h (iv) Ar′COCH_3_, 50% NaOH, dioxane : ethanol (1 : 4), r.t (v) NH_2_NH_2_·H_2_O/HCOOH, reflux for 4–6 h (vi) NH_2_NH_2_·H_2_O/CH_3_COOH, reflux for 8–12 h.

5 mL of formic acid and 1 mmol chalcone derivatives (6a–6d) were mixed with 1 mL hydrazine hydrate and heated under reflux for 4–6 h. TLC with EtOAc/hexane (1 : 3) as mobile phase was used to monitor the reaction progress. After the consume of chalcones, the hot reaction mixture was poured into ice-cold water. Precipitates were then filtered, washed with water, dried, and recrystallized in chloroform, and the affordable pyrazoline compounds (7a–7d) obtained are shown in Scheme 1.

#### 3.1.6 General procedure for the synthesis of compounds 8a–8h

The mixture of chalcone derivatives (6a–6d) (1 mmol) and phenyl/4-methoxy phenyl boronic acid (121.93/151.96 mg) were refluxed at 100 °C for 4–12 h in solvent ratio 1 : 1 : 1 (H_2_O : THF : Toluene) at N_2_ as inert atmosphere in the presence of catalyst Pd(PPh_3_)_4_ (5 mol%). The progress of the reaction was monitored by TLC. After the completion of the reaction, the reaction mixture is extracted with EtOAc (5 × 30 mL). The organic layer was dried with anhydrous Na_2_SO_4_ and concentrated under reduced pressure. The precipitates obtained were filtered, washed, dried and recrystallized in ethanol.

#### 3.1.7 General procedure for the synthesis of compounds 9a–9d

1 mmol of chalcone derivatives (6a–6d) in 5 mL acetic acid and 1 mL hydrazine hydrate reaction mixture were refluxed for 8–12 h. TLC with EtOAc/hexane (1 : 3) as mobile phase was used to monitor reaction progress. After the consume of chalcones, the hot reaction mixture was poured into ice-cold water. The precipitate was then filtered, washed with water, dried, and recrystallized in chloroform to obtain the affordable pyrazoline compounds. The synthetic reactions are shown in Scheme 1.

##### 3.1.7.1 1-(4-Bromophenyl)-3-(4-methoxyphenyl)-1*H*-pyrazole-4-carbaldehyde (4)

White solid; yield 68%; mp 168–170 °C; IR (neat, *ν*_max_): (–C=O str.) 3130, (–C=N str.) 1678 cm^−1^; ^1^H NMR (Chloroform-D, *δ* ppm): 8.52 (s, 1H), 7.78–7.80 (d, 2H), 7.69–7.72 (d, 2H), 7.64–7.66 (m, 3H), 7.04–7.06 (d, 2H), 3.90 (s, 3H); ^13^C NMR (CDCl_3_, *δ*): 185.12, 160.66, 154.73, 138.04, 132.77, 131.03, 130.26, 123.58, 122.62, 121.36, 121.07, 114.26, 55.41, ESI-MS. Calcd for C_17_H_13_BrN_2_O_2_, [M + H]^+^: *m*/*z* 356.02. Found: *m*/*z* 356.95.

##### 3.1.7.2 1-([1,1′-Biphenyl]-4-yl)-3-(4-methoxyphenyl)-1*H*-pyrazole-4-carbaldehyde (5a)

White solid; yield 65%; mp 220–225 °C; IR (neat, *ν*_max_): (–C=O str.) 2925, (–C=N str.) 1674 cm^−1^; ^1^H NMR (Chloroform-D, *δ* ppm): *δ* 8.58 (s, 1H), 7.87–7.90 (m, 4H), 7.82–7.84 (d, 2H), 7.74–7.76 (d, 2H), 7.64–7.66 (d, 2H), 7.05–7.08 (m, 2H), 3.91 (s, 3H); ^13^C NMR (CDCl_3_, *δ*): 185.09, 160.67, 154.74, 140.82, 139.87, 138.09, 131.07, 130.30, 128.98, 128.26, 127.84, 127.04, 123.67, 119.97, 114.34, 55.26, ESI-MS. Calcd for C_23_H_18_N_2_O_2_, [M + H]^+^: *m*/*z* 354.14. Found: *m*/*z* 355.21.

##### 3.1.7.3 1-(4′Methoxy-([1,1′-biphenyl]-4-yl))-3-(4-methoxyphenyl)-1*H*-pyrazole-4-carbaldehyde (5b)

White solid; yield 68%; mp 210–212 °C; IR (neat, *ν*_max_): (–C=O str.) 2921, (–C=N str.) 1607 cm^−1^; ^1^H NMR (Chloroform-D, *δ* ppm): 8.56 (s, 1H), 7.82–7.84 (d, 2H), 7.69–7.71 (d, 2H), 7.57–7.59 (d, 2H), 7.49–7.51 (d, 2H), 6.97–7.07 (m, 4H), 3.86 (s, 3H); ^13^C NMR (CDCl_3_, *δ*): 185.27, 160.58, 159.58, 158.69, 154.58, 140.47, 137.63, 132.06, 131.04, 130.30, 128.10, 127.74, 123.85, 122.34, 119.99, 114.42, 114.23, 114.18, 55.36, ESI-MS. Calcd for C_24_H_20_N_2_O_3_, [M + H]^+^: *m*/*z* 384.15. Found: *m*/*z* 385.26.

##### 3.1.7.4 (*E*)-3-((1-Bromophenyl)-3-(4-methoxyphenyl)-1*H*-pyrazol-4-yl)-1-phenylprop-2-en-1-one (6a)

Yellow solid; yield 67%; mp 180–185 °C; IR (neat, *ν*_max_): (–C=O str.) 1596, (–C=N str.) 1663 cm^−1^; ^1^H NMR (Chloroform-D, *δ* ppm): 8.27 (s, 1H), 7.86–7.88 (d, 2H), 7.81–7.85 (d, 2H), 7.56–7.68 (m, 6H), 7.32–7.37 (d, 2H), 7.24–7.27 (d, 3H), 6.99–7.03 (m, 3H), 3.83 (s, 3H); ^13^C NMR (CDCl_3_, *δ*): 189.63, 160.38, 154.03, 144.66, 143.74, 138.65, 135.78, 134.93, 132.43, 130.20, 129.94, 128.71, 128.48, 126.61, 124.77, 121.82, 120.77, 120.51, 120.35, 118.77, 114.48, 114.25, 55.57, 21.85, ESI-MS. Calcd for C_25_H_19_BrN_2_O_2_, [M + H]^+^: *m*/*z* 459.97. Found: *m*/*z* 458.06.

##### 3.1.7.5 (*E*)-3-(1-(4-Bromophenyl)-3-(4-methoxyphenyl)-1*H*-pyrazol-4-yl)-1-(4-(methylthio)phenyl)prop-2-en-1-one (6b)

Yellow solid; yield 71%; mp 225–228 °C; IR (neat, *ν*_max_): (–C=O str.) 1592, (–C=N str.) 1648 cm^−1^; ^1^H NMR (Chloroform-D, *δ* ppm): 8.29 (s, 1H), 7.94–7.96 (d, 2H), 7.83–7.88 (d, 2H), 7.75–7.78 (d, 2H), 7.48–7.75 (m, 3H), 7.44–7.47 (d, 2H), 7.25–7.38 (d, 2H), 7.00–7.04 (d, 2H), 3.86 (s, 3H); ^13^C NMR (CDCl_3_, *δ*): 190.21, 160.42, 154.13, 138.66, 138.40, 135.44, 132.95, 132.91, 132.80, 131.24, 130.44, 130.22, 128.80, 128.58, 126.67, 124.74, 121.83, 121.25, 120.83, 120.61, 118.73, 114.51, 55.60, ESI-MS. Calcd for C_26_H_21_N_2_O_2_S, [M + H]^+^: *m*/*z* 505.43. Found: *m*/*z* 506.05.

##### 3.1.7.6 (*E*)-3-(1-(4-bromophenyl)-3-(4-methoxyphenyl)-1*H*-pyrazol-4-yl)-1-(*p*-tolyl)prop-2-en-1-one (6c)

Yellow solid; yield 71%; mp 218–222 °C; IR (neat, *ν*_max_): (–C=O str.) 1603, (–C=N str.) 1663 cm^−1^; ^1^H NMR (Chloroform-D, *δ* ppm): 8.29 (s, 1H), 7.85–7.89 (d, 2H), 7.75–7.78 (d, 2H), 7.52–7.74 (m, 4H), 7.20–7.25 (d, 2H), 7.13–7.16 (d, 2H), 6.99–7.07 (d, 3H), 3.87 (s, 3H); ^13^C NMR (CDCl_3_, *δ*): 181.93, 160.44, 154.12, 145.76, 138.65, 134.69, 134.14, 133.86, 132.81, 132.59, 132.49, 131.67, 130.26, 129.95, 128.42, 126.86, 124.74, 121.50, 120.84, 120.62, 120.45, 118.52, 114.52, 114.37, 55.61, 44.80 ESI-MS. Calcd for C_26_H_21_BrN_2_O_2_, [M + H]^+^: *m*/*z* 472.08. Found: *m*/*z* 473.06.

##### 3.1.7.7 (*E*)-3-(1-(4-Bromophenyl)-3-(4-methoxyphenyl)-1*H*-pyrazol-4-yl)-1-(thiophen-2-yl)prop-2-en-1-one (6d)

Yellow solid; yield 76%; mp 190–195 °C; IR (neat, *ν*_max_): (–C=O str.) 1592, (–C=N str.) 1663 cm^−1^; ^1^H NMR (Chloroform-D, *δ* ppm): 8.28 (s, 1H), 7.94–7.98 (d, 2H), 7.80–7.93 (d, 2H), 7.75–7.78 (d, 2H), 7.55–7.68 (m, 4H), 7.26–7.37 (m, 4H), 6.91–7.02 (m, 4H), 3.84 (s, 3H). ^13^C NMR (CDCl_3_, *δ*): 188.38, 163.56, 160.35, 153.96, 138.66, 134.51, 132.91, 132.74, 131.24, 130.85, 130.42, 130.19, 126.58, 124.81, 121.65, 121.21, 120.75, 120.47, 118.82, 114.46, 114.01, 55.60, ESI-MS. Calcd for C_23_H_17_BrN_2_O_2_S, [M + H]^+^: *m*/*z* 466.02. Found: *m*/*z* 466.93.

##### 3.1.7.8 1′-(4-Bromophenyl)-3′-(4-methoxyphenyl)-5-phenyl-3,4-dihydro-1′*H*,2*H*-[3,4′-bipyrazole]-2-carbaldehyde (7a)

White solid; yield 77%; mp 202–205 °C; IR (neat, *ν*_max_): (–C=O str.) 1596, (–C=N str.) 1674 cm^−1^; ^1^H NMR (Chloroform-D, *δ* ppm): 8.99 (s, 1H), 7.78 (s, 1H), 7.66–7.63 (d, 2H), 7.58–7.56 (d, 2H), 7.53–7.50 (d, 2H), 7.41–7.39 (m, 4H), 6.96–6.94 (d, 2H), 5.82–5.78 (dd, 1H), 3.81 (s, 3H), 3.71–3.64 (dd, 1H), 3.16–3.10 (dd, 1H); ^13^C NMR (CDCl_3_, *δ*): 160.44, 160.03, 154.98, 150.74, 138.5, 133.72, 133.01, 132.58, 130.47, 129.47, 129.75, 126.04, 125.40, 125.28, 123.15, 121.44, 120.58, 119.89, 114.40, 55.52, 51.78, 42.13, ESI-MS. Calcd for C_26_H_21_BrN_4_O_2_, [M + H]^+^: *m*/*z* 501.18. Found: *m*/*z* 503.08.

##### 3.1.7.9 1′-(4-Bromophenyl)-3′-(4-methoxyphenyl)-5-(*p*-tolyl)-3,4-dihydro-1′*H*,2*H*-[3,4′-bipyrazole]-2-carbaldehyde (7b)

White solid; yield 78%; mp 221–224 °C; IR (neat, *ν*_max_): (–C=O str.) 1592, (–C=N str.) 1674 cm^−1^; ^1^H NMR (Chloroform-D, *δ* ppm): 8.97 (s, 1H), 7.77 (s, 1H), 7.63–7.65 (d, 2H), 7.50–7.57 (m, 7H), 7.20–7.18 (d, 2H), 6.96–6.94 (d, 2H), 5.80–5.76 (dd, 1H), 3.81 (s, 3H), 3.66–3.61 (dd, 1H), 3.14–3.08 (dd, 1H), 2.37 (s, 3H). ^13^C NMR (CDCl_3_, *δ*): 160.33, 159.96, 156.52, 150.68, 141.41, 139.00, 132.95, 132.79, 132.54, 130.01, 129.75, 129.69, 128.18, 126.85, 125.92, 125.41, 121.80, 120.81, 120.54, 119.79, 114.39, 55.50, 51.51, 42.47, 21.70, ESI-MS. Calcd for C_27_H_23_BrN_4_O_2_, [M + H]^+^: *m*/*z* 515.00. Found: *m*/*z* 514.10.

##### 3.1.7.10 1′-(4-Bromophenyl)-3′-(4-methoxyphenyl)-5-(4-(methylthio)phenyl)-3,4-dihydro-1′*H*,2*H*-[3,4′-bipyrazole]-2-carbaldehyde (7c)

White solid; yield 79%; mp 240–244 °C; IR (neat, *ν*_max_): (–C=O str.) 1592, (–C=N str.) 1670 cm^−1^; ^1^H NMR (Chloroform-D, *δ* ppm): 8.97 (s, 1H), 7.78 (s, 1H), 7.68–7.49 (m, 4H), 7.30–7.27 (d, 2H), 7.25–7.21 (d, 2H), 6.96–6.93 (dd, 1H), 5.81–5.75 (dd, 1H), 3.81 (s, 3H), 3.68–3.58 (dd, 1H), 3.13–3.05 (dd, 1H), 2.49 (s, 3H). ^13^C NMR (CDCl_3_, *δ*): 160.31, 159.99, 156.02, 150.71, 142.78, 138.99, 138.63, 132.81, 132.56, 130.20, 130.24, 129.77, 129.03, 127.33, 127.16, 126.70, 125.97, 125.38, 125.32, 121.70, 120.83, 120.56, 119.83, 114.39, 55.51, 51.58, 42.29, 15.32, ESI-MS. Calcd for C_27_H_23_BrN_4_O_2_S, [M + H]^+^: *m*/*z* 548.12. Found: *m*/*z* 548.07.

##### 3.1.7.11 1′-(4-Bromophenyl)-3′-(4-methoxyphenyl)-5-(thiophen-2-yl)-3,4-dihydro-1′*H*,2*H*-[3,4′-bipyrazole]-2-carbaldehyde (7d)

White solid; yield 73%; mp 207–210 °C; IR (neat, *ν*_max_): (–C=O str.) 1503, (–C=N str.) 1666 cm^−1^; ^1^H NMR (Chloroform-D, *δ* ppm): 8.94 (s, 1H), 7.79 (s, 1H), 7.64–7.61 (d, 2H), 7.58–7.56 (d, 2H), 7.53–7.51 (d, 2H), 7.44–7.43 (d, 2H), 7.13–7.12 (d, 2H), 7.04–7.02 (d, 2H), 6.96–6.94 (d, 2H) 5.82–5.78 (dd, 1H), 3.82 (s, 3H), 3.71–3.64 (dd, 1H), 3.13–3.08 (dd, 1H). ^13^C NMR (CDCl_3_, *δ*): 160.42, 160.01, 155.33, 150.73, 138.96, 136.98, 132.58, 129.79, 129.48, 129.26, 128.10, 126.09, 125.33, 121.48, 120.58, 119.89, 114.39, 55.50, 51.78, 42.17, ESI-MS. Calcd for C_24_H_19_BrN_4_O_2_S, [M + H]^+^: *m*/*z* 508.15. Found: *m*/*z* 507.41.

##### 3.1.7.12 (*E*)-3-(1-([1,1′-Biphenyl]-4-yl)-3-(4-methoxyphenyl)-1*H*-pyrazol-4-yl)-1-phenylprop-2-en-1-one (8a)

Yellow solid; yield 78%; mp 250–252 °C; IR (neat, *ν*_max_): (–C=O str.) 1529, (–C=N str.) 1663 cm^−1^; ^1^H NMR (Chloroform-D, *δ* ppm): 8.33 (s, 1H), 8.28–8.27 (s, 1H), 8.00–7.90 (d, 2H), 7.80–7.88 (d, 2H), 7.78–7.58 (m, 5H), 7.56–7.48 (d, 2H), 7.44–7.29 (m, 4H), 7.06–7.04 (d, 2H), 3.90 (s, 3H). ^13^C NMR (CDCl_3_, *δ*): 190.19, 160.24, 153.99, 138.45, 138.17, 135.65, 135.38, 133.57, 132.78, 132.71, 132.63, 131.14, 130.10, 130.05, 128.94, 128.63, 128.42, 128.17, 128.00, 127.01, 126.53, 124.51, 121.62, 121.34, 120.67, 120.46, 119.58, 118.53, 114.33, 55.41, ESI-MS. Calcd for C_32_H_26_N_2_O_2_, [M + H]^+^: *m*/*z* 456.55. Found: *m*/*z* 458.98.

##### 3.1.7.13 (*E*)-3-(1-(4′-Methoxy-[1,1′-biphenyl]-4-yl)-3-(4-methoxyphenyl)-1*H*-pyrazol-4-yl)-1-phenylprop-2-en-1-one (8b)

Yellow solid; yield 72%; mp 209–211 °C; IR (neat, *ν*_max_): (–C=O str.) 1529, (–C=N str.) 1637 cm^−1^; ^1^H NMR (Chloroform-D, *δ* ppm): 8.39 (s, 1H), 8.01–7.99 (s, 1H), 7.87–7.85 (d, 2H), 7.70–6.68 (d, 2H), 7.60–7.58 (d, 2H), 7.53–7.50 (d, 2H), 7.43–7.39 (m, 4H), 7.29–7.28 (d, 2H), 7.07–7.02 (d, 2H), 3.91 (s, 3H). ^13^C NMR (CDCl_3_, *δ*): 189.90, 160.23, 159.34, 158.34, 153.61, 138.49, 138.43, 138.31, 138.07, 135.69, 132.66, 132.42, 132.06, 130.09, 128.60, 128.40, 128.05, 127.65, 126.56, 124.86, 124.62, 121.25, 119.60, 118.18, 114.39, 55.40, ESI-MS. Calcd for C_33_H_28_N_2_O_3_, [M + H]^+^: *m*/*z* 486.19. Found: *m*/*z* 487.28.

##### 3.1.7.14 (*E*)-3-(1-([1,1′-Biphenyl]-4-yl)-3-(4-methoxyphenyl)-1*H*-pyrazol-4-yl)-1-(4(methylthio) phenyl)prop-2-en-1-one (8c)

Yellow solid; yield 80%; mp 240–242 °C; IR (neat, *ν*_max_): (–C=O str.) 1599, (–C=N str.) 1659 cm^−1^; ^1^H NMR (Chloroform-D, *δ* ppm): 8.40 (s, 1H), 8.28–8.27 (s, 1H), 7.94–7.89 (d, 2H), 7.75–7.74 (d, 2H), 7.70–7.65 (d, 2H), 7.56–7.54 (d, 2H), 7.53–7.49 (d, 2H), 7.44–7.41 (d, 2H), 7.33–7.31 (d, 2H), 7.07–7.05, (d, 2H), 3.91 (s, 3H), 2.46 (s, 3H). ^13^C NMR (CDCl_3_, *δ*): 188.82, 160.18, 153.87, 145.58, 140.07, 139.98, 138.64, 135.32, 134.53, 130.17, 129.03, 128.94, 128.23, 127.08, 125.12, 120.99, 119.63, 119.60, 119.57, 118.36, 114.40, 114.38, 114.35, 114.32, 55.49, 29.81 ESI-MS. Calcd for C_23_H_17_N_3_O, [M + H]^+^: *m*/*z* 502.17. Found: *m*/*z* 503.08.

##### 3.1.7.15 (*E*)-3-(1-(4′-Methoxy-[1,1′-biphenyl]-4-yl)-3-(4-methoxyphenyl)-1*H*-pyrazol-4-yl)-1-(4-(methylthio)phenyl)prop-2-en-1-one (8d)

Yellow solid; yield 76%; mp 248–250 °C; IR (neat, *ν*_max_): (–C=O str.) 1544, (–C=N str.) 1659 cm^−1^; ^1^H NMR (Chloroform-D, *δ* ppm): 8.38 (s, 1H), 7.93–7.90 (d, 2H), 7.87–7.85 (d, 2H), 7.70–7.68 (d, 2H), 7.60–7.58 (d, 2H), 7.51–7.49 (d, 2H), 7.43–7.40 (m, 4H), 7.32–7.28 (d, 2H), 7.07–7.02 (d, 2H), 3.89 (s, 3H), 2.46 (s, 3H). ^13^C NMR (CDCl_3_, *δ*): 188.99, 159.53, 153.60, 137.48, 135.56, 135.48, 135.35, 132.15, 130.10, 128.86, 128.56, 128.11, 127.64, 126.60, 126.44, 125.11, 120.98, 120.86, 119.60, 116.07, 114.36, 113.44, 55.41, 29.71 ESI-MS. Calcd for C_30_H_24_N_2_O_3_S, [M + H]^+^: *m*/*z* 532.18. Found: *m*/*z* 533.39.

##### 3.1.7.16 (*E*)-3-(1-([1,1′-Biphenyl]-4-yl)-3-(4-methoxyphenyl)-1*H*-pyrazol-4-yl)-1-(*p*-tolyl)prop-2-en-1-one (8e)

Yellow solid; yield 68%; mp 260–262 °C; IR (neat, *ν*_max_): (–C=O str.) 1611, (–C=N str.) 1633 cm^−1^; ^1^H NMR (Chloroform-D, *δ* ppm): 8.39 (s, 1H), 7.94–7.88 (d, 2H), 7.75–7.65 (d, 2H), 7.52–7.48 (d, 2H), 7.44–7.39 (d, 2H), 7.32–7.30 (d, 2H), 7.07–7.05 (d, 2H), 3.90 (s, 3H), 2.45 (s, 3H). ^13^C NMR (CDCl_3_, *δ*): 189.67, 160.12, 153.77, 143.55, 140.01, 138.61, 135.67, 135.16, 132.15, 132.05, 130.09, 129.32, 128.94, 128.56, 128.15, 127.68, 127.00, 126.57, 124.80, 121.37, 119.55, 118.32, 114.29, 55.41, 29.72 ESI-MS. Calcd for C_32_H_36_N_2_O_2_, [M + H]^+^: *m*/*z* 470.20. Found: *m*/*z* 471.34.

##### 3.1.7.17 (*E*)-3-(1-(4′-Methoxy-[1,1′-biphenyl]-4-yl)-3-(4-methoxyphenyl)-1*H*-pyrazol-4-yl)-1-(*p*-tolyl)prop-2-en-1-one (8f)

Yellow solid; yield 65%; mp 280–282 °C; IR (neat, *ν*_max_): (–C=O str.) 1611, (–C=N str.) 1633 cm^−1^; ^1^H NMR (Chloroform-D, *δ* ppm): 8.37 (s, 1H), 7.93–7.89 (d, 2H), 7.86–7.84 (d, 2H), 7.70–7.67 (d, 2H), 7.60–7.58 (d, 2H), 7.51–7.49 (d, 2H), 7.43–7.39 (m, 4H), 7.32–7.28 (d, 2H), 7.07–7.01 (d, 2H), 3.89 (s, 3H), 2.45 (s, 3H). ^13^C NMR (CDCl_3_, *δ*): 189.65, 160.10, 159.45, 153.70, 143.52, 138.10, 135.69, 135.19, 130.09, 129.31, 128.55, 128.04, 127.62, 126.53, 124.84, 121.29, 119.57, 118.23, 114.38, 114.28, 55.41, 29.72 ESI-MS. Calcd for C_33_H_28_N_2_O_3_, [M + H]^+^: *m*/*z* 500.21. Found: *m*/*z* 501.32.

##### 3.1.7.18 (*E*)-3-(1-([1,1′-Biphenyl]-4-yl)-3-(4-methoxyphenyl)-1*H*-pyrazol-4-yl)-1-(thiophen-2-yl)prop-2-en-1-one (8g)

Yellow solid; yield 78%; mp 275–277 °C; IR (neat, *ν*_max_): (–C=O str.) 1596, (–C=N str.) 1640 cm^−1^; ^1^H NMR (Chloroform-D, *δ* ppm): 8.32 (s, 1H), 7.93–7.88 (d, 2H), 7.82–7.77 (d, 2H), 7.70–7.64 (d, 2H), 7.54–7.48 (d, 2H), 7.43–7.41 (d, 2H), 7.19–7.17 (m, 4H), 7.07–7.04 (d, 2H), 3.90 (s, 3H). ^13^C NMR (CDCl_3_, *δ*): 191.14, 160.24, 153.97, 145.44, 134.62, 133.70, 132.62, 132.14, 132.04, 128.73, 128.61, 127.00, 121.25, 120.67, 120.57, 119.59, 115.37, 114.33, 55.33 ESI-MS. Calcd for C_29_H_22_N_2_O_2_S, [M + H]^+^: *m*/*z* 462.14. Found: *m*/*z* 463.25.

##### 3.1.7.19 (*E*)-3-(1-(4′-Methoxy-[1,1′-biphenyl]-4-yl)-3-(4-methoxyphenyl)-1*H*-pyrazol-4-yl)-1-(thiophen-2-yl)prop-2-en-1-one (8h)

Yellow solid; yield 84%; mp 268–270 °C; IR (neat, *ν*_max_): (–C=O str.) 1599, (–C=N str.) 1655 cm^−1^; ^1^H NMR (Chloroform-D, *δ* ppm): 8.32 (s, 1H), 8.20–8.18 (d, 2H), 7.94–7.77 (m, 2H), 7.72–7.57 (d, 2H), 7.51–7.49 (d, 2H), 7.24–7.17 (d, 2H), 7.10–6.92 (d, 2H), 3.90 (s, 3H). ^13^C NMR (CDCl_3_, *δ*): 181.40, 160.25, 145.44, 132.65, 132.15, 132.05, 130.08, 128.68, 128.56, 128.25, 127.24, 121.27, 120.67, 119.61, 118.32, 116.07, 114.78, 114.38, 114.33, 114.18, 113.46, 55.36 ESI-MS. Calcd for C_30_H_24_N_2_O_3_S, [M + H]^+^: *m*/*z* 492.15. Found: *m*/*z* 493.28.

##### 3.1.7.20 1-(1′-(4-Bromophenyl)-3′-(4-methoxyphenyl)-5-phenyl-3,4-dihydro-1′*H*,2*H*-[3,4′-bipyrazol]-2-yl)ethan-1-one (9a)

White solid; yield 82%; mp 185–187 °C; IR (neat, *ν*_max_): (–C=O str.) 1596, (–C=N str.) 1663 cm^−1^; ^1^H NMR (Chloroform-D, *δ* ppm): 7.80 (s, 1H), 7.67–7.57 (d, 2H), 7.42–7.40 (d, 2H), 7.39–7.35 (d, 2H), 7.33–7.25 (d, 2H), 6.97–6.94 (d, 2H), 5.85–5.79 (dd, 1H), 3.81 (s, 3H), 3.83–3.73 (dd, 1H), 3.31–3.23 (dd, 1H), 2.43 (s, 3H). ^13^C NMR (CDCl_3_, *δ*): 169.65, 159.93, 154.10, 150.93, 139.08, 133.18, 132.53, 131.17, 131.11, 130.54, 130.50, 129.90, 127.11, 125.89, 125.58, 122.67, 120.59, 119.71, 114.32, 55.50, 52.72, 44.90, 22.28. ESI-MS. Calcd for C_27_H_23_BrN_4_O_2_, [M + H]^+^: *m*/*z* 514.10. Found: *m*/*z* 515.00.

##### 3.1.7.21 1-(1′-(4-Bromophenyl)-3′-(4-methoxyphenyl)-5-(4-(methylthio)phenyl)-3,4-dihydro-1′*H*,2*H*-[3,4′-bipyrazol]-2-yl)ethan-1-one (9b)

White solid; yield 85%; mp 214–217 °C; IR (neat, *ν*_max_): (–C=O str.) 1592, (–C=N str.) 1655, cm^−1^; ^1^H NMR (Chloroform-D, *δ* ppm): 7.72 (s, 1H), 7.66–7.64 (d, 2H), 7.63–7.49 (d, 2H), 7.42–7.40 (d, 2H), 7.11–7.10 (d, 2H) 7.09–6.92 (d, 2H), 5.87–5.82 (dd, 1H), 3.82 (s, 3H), 3.66–3.57 (dd, 1H), 3.09–3.01 (dd, 1H), 2.40 (s, 3H). ^13^C NMR (CDCl_3_, *δ*): 169.09, 167.79, 159.28, 150.71, 150.28, 139.04, 134.95, 132.51, 129.80, 129.10, 128.99, 127.80, 125.86, 125.51, 122.67, 120.55, 119.70, 114.31, 55.51, 52.61, 42.76, 22.21, 20.86. ESI-MS. Calcd for C_28_H_25_BrN_4_O_2_S, [M + H]^+^: *m*/*z* 560.09. Found: *m*/*z* 561.16.

##### 3.1.7.22 1-(1′-(4-Bromophenyl)-3′-(4-methoxyphenyl)-5-(*p*-tolyl)-3,4-dihydro-1′*H*,2*H*-[3,4′-bipyrazol]-2-yl)ethan-1-one (9c)

White solid; yield 82%; mp 197–199 °C; IR (neat, *ν*_max_): (–C=O str.) 1592, (–C=N str.) 1663 cm^−1^; ^1^H NMR (Chloroform-D, *δ* ppm): 7.74 (s, 1H), 7.67–7.63 (d, 2H), 7.58–7.49 (d, 2H), 7.42–7.36 (d, 2H), 6.96–6.91 (d, 2H), 5.88–5.83 (dd, 1H), 3.80 (s, 3H), 3.65–3.56 (dd, 1H), 3.11–3.04 (dd, 2H), 2.46 (s, 3H), 2.08 (s, 3H). ^13^C NMR (CDCl_3_, *δ*): 169.45, 159.87, 154.83, 150.74, 139.04, 132.49, 131.37, 130.66, 129.81, 128.89, 126.79, 125.86, 125.55, 122.80, 120.57, 119.67, 114.30, 55.48, 52.52, 42.06, 22.24, 20.91. ESI-MS. Calcd for C_28_H_25_BrN_4_O_2_, [M + H]^+^: *m*/*z* 529.10. Found: *m*/*z* 528.12.

##### 3.1.7.23 1-(1′-(4-Bromophenyl)-3′-(4-methoxyphenyl)-5-(thiophen-2-yl)-3,4-dihydro-1′*H*,2*H*-[3,4′-bipyrazol]-2-yl)ethan-1-one (9d)

White solid; yield 85%; mp 191–193 °C; IR (neat, *ν*_max_): (–C=O str.) 1568, (–C=N str.) 1666 cm^−1^; ^1^H NMR (Chloroform-D, *δ* ppm): *δ* 7.72 (s, 1H), 7.69–7.64 (d, 2H), 7.55–7.47 (d, 2H), 7.25–7.17 (d, 2H), 6.97–6.92 (d, 2H), 5.85–5.80 (dd, 1H), 3.81 (s, 3H), 3.63–3.54 (dd, 1H), 3.09–3.02 (dd, 1H), 2.44 (s, 3H). ^13^C NMR (CDCl_3_, *δ*): 169.12, 159.85, 154.65, 150.70, 140.98, 139.06, 132.47, 129.79, 129.59, 128.65, 126.73, 125.69, 125.61, 123.02, 120.51, 119.60, 114.29, 55.47, 52.39, 42.17, 21.68. ESI-MS. Calcd for C_25_H_21_BrN_4_O_2_S, [M + H]^+^: *m*/*z* 522.05. Found: *m*/*z* 523.00.

### 3.2 *In vitro* cytotoxicity

The cytotoxicity evaluation of compounds 4, 5a–5b, 6a–6d, 7a–7d, 8a–8h, and 9a–9d was performed using MTT assay on human adenocarcinoma (A549) and cervical cancer (HeLa) cell lines purchased from ATCC. The chemical compounds were tested at 24 h and 48 h with various concentrations ranging from 0 to 200 μM. The following day, cells were treated with various concentrations of chemical compounds and incubated for 24 h and 48 h at 37 °C with 5% CO_2_. After that, 0.5 mg mL^−1^ MTT was added to each well and incubated for 4 h. Next, the solution was removed, and 150 μl of dimethyl sulfoxide (DMSO) was added to the wells. Then, the plate was read at 575 nm by Infinite M200 pro. The results were analyzed as the percentage of viable cells to the concentration of the compounds.^43^

### 3.3 DNA binding study and antioxidant activity

The Calf-thymus DNA interactions and antioxidant activity of the active compound have been studied by a previously reported method.^44,45^

### 3.4 MD simulation

#### 3.4.1 Data processing and preparations

The crystal structure of the EGFR tyrosine kinase domain with erlotinib with PDBID 4HJO was downloaded from https://www.rcsb.org/ database and prepared with Protein preparation workflow (PPW) in Schrodinger Maestro to preprocess, optimize, and minimize the structure. The protein termini were capped and filled the missing side chains, assigned bond orders using the CCD database, replaced hydrogen and created zero-order bonds to metal and disulphide bonds. Added termini oxygen to protein chains, converted selenomethionines to methionine, and filled missing loops using prime. The hetero state was generated using the Epik module at pH 7 ± 2. Sample water orientation was chosen, crystal symmetry was used, and then the protein was optimized with PROPKA. In the minimization option, the convergence of heavy atoms to RMSD was 0.30 Å, water molecules beyond 5 Å to ligand were deleted, and then the protein was minimized with OPLS4 force field. The ligand structure was drawn with a 2-D sketcher and then prepared with the LigPrep tool to generate the possible states in 3-D for molecular docking. The ligand size filter was kept for 500 atoms and OPLS4 force field was used for the ligand preparation. Ionisation was performed to generate the possible states at a target pH of 7 ± 2 and the Epik was used while adding metal binding states and including the original states along with the desalt and generating tautomers. The stereoisomer computations were kept while retaining the specified chiralities to generate at most 32 states per compound 9d. The LigPrep generated two states to help the best during the docking.^46,47^

#### 3.4.2 Grid generation and molecular docking

For molecular docking, we have used the Glide, which is a grid-based ligand docking tool in Schrodinger Maestro. The protein is already with a native ligand and has been used as the active site. In the receptor tab, the pick to identify the ligand molecule option was checked, and the native ligand was selected in the workspace to put the grid on the centroid of the workspace ligand and then the size was extended to get the best fit on the active site. The molecular docking was performed with extra precise (XP) sampling algorithms in Glide followed by molecular mechanics with generalized born and surface area solvation (MM/GBSA) calculation in a flexible state and computed the absorption, distribution, metabolism, excretion, and toxicity (ADMET) properties with the QikProp tool in Schrödinger Maestro while keeping Epik state penalties.^48^

#### 3.4.3 Molecular dynamics simulation

The docked pose was merged for the molecular dynamics (MD) simulation to get one protein and ligand PDB file. We have used the Desmond package for the molecular dynamics simulation developed by the D. E. Shaw research after obtaining the academic license. The system builder tool was used to prepare the system for further production runs. In the solvation tab, we have taken the SPC solvent model in orthorhombic boundary conditions in a buffer state at a distance of 10 × 10 × 10 Å and then minimized the volume of the boundary to get it fit at best on the protein. The ion and salt placement within 20 Å was excluded, and 3 Cl^−^ ions were added to neutralize the system and used the OPLS4 force field.^49^ The system builder has produced 36 665 atoms for the complete protein, added ions, and water as a solute medium. The production run was kept for 100 ns to record the trajectory at 100 ps at an energy level of 1.2 that has produced 1000 frames. The NPT ensemble class was used at 300 K temperature and 1.01325 pressure (bar) and then relaxed the model system before simulation. The trajectories were analyzed with the help of a simulation interaction diagram tool to understand the deviation, fluctuation, and intermolecular interactions.

## 4. Conclusions

In this study, a series of novel pyrazole–pyrazoline derivatives have been synthesized. Structures of all synthesized compounds were fully characterized by detailed FT-IR, ^1^H NMR, ^13^C NMR, and mass spectrometry. Compound 9d exhibited significant anti-proliferative activity against A549 (lung cancer) and HeLa (human cervix) cancer cell lines. By utilizing UV-visible, fluorescence, circular dichroism, and cyclic voltammetry, the binding affinity of the active analog 9d with Ct-DNA was investigated. The molecular dynamics analysis of derivative 9d was also carried out with the KGFR receptor.

## Conflicts of interest

There are no conflicts to declare.

## Supplementary Material

RA-013-D3RA04873J-s001

RA-013-D3RA04873J-s002
