# Supplementary material for: Design, synthesis, X-ray crystal structures, anticancer, DNA binding, and molecular modelling studies of pyrazole–pyrazoline hybrid derivatives
Source: RSC Adv. 2023 Sep 6;13(38):26766–79. doi: 10.1039/d3ra04873j (PMC10481259; doi:10.1039/d3ra04873j)
Supplement: RA-013-D3RA04873J-s001 [file RA-013-D3RA04873J-s001.pdf]

Electronic Supplementary Material (ESI) for RSC Advances.
This journal is © The Royal Society of Chemistry 2023

Design, synthesis, X-ray crystal structures, anticancer, DNA binding and molecular modelling studies of pyrazole–pyrazoline hybrid derivatives

Manish Rana[a,b], Hungharla Hungyo[c], Palak Parashar[c], Shaban Ahmad[d], Rabiya Mehandi[a], Vibha Tandon[c], Khalid Raza[d], Mohammed A. Assiri[e], Tarik E. Ali[e], Zeinhom M. El-Bahy[f], Rahisuddin[a, *]

[a]*Molecular and Biophysical Research Lab (MBRL), Department of Chemistry, Jamia Millia Islamia, New Delhi 110025, INDIA*

[b]*Department of Chemistry, Ramjas College, University of Delhi, Delhi 110007, INDIA*

[c]*Special Centre for Molecular Medicine, Jawaharlal Nehru University, New Delhi 110067, INDIA*

[d]*Department of Computer Science, Jamia Millia Islamia, New Delhi 110025, INDIA*

[e]*Department of Chemistry, Faculty of Science, King Khalid University, Abha, 61421 Saudi Arabia*

[f]*Department of Chemistry, Faculty of Science, Al-Azhar University, Nasr City 11884, Cairo, Egypt*

*Author for correspondence:
rahisuddin@jmi.ac.in (+919871460479)

Materials and methods

All the reagents are commercially available were used as received without further purification. Substituted acetophenones (Spectrochem), formic acid (Fisher scientific), hydrazine hydrate (S.D. Fine Chemicals), and catalyst $Pd(PPh_3)_4$ Alfa Aesar. Melting points have been determined using open capillary tube and are uncorrected. Reaction progress was routinely monitored by TLC (thin layer chromatography) on silica gel (precoated 60 F_{254}Al

sheets, Merck). IR spectra of neat compounds were rerecorded on Agilent Technologies and expressed in wavenumber (cm^{-1}). ^{1}H and ^{13}C-NMR spectra were recorded using $CDCl_3$ as solvent on a Bruker 300 MHz spectrometer. Chemical shifts (δ) are given in ppm and tetramethylsilane (TMS) used as reference. To investigate the DNA-drug interaction, IVIUM potentiostat was used with a three-electrode system that was obtained from Metrohm, Dropsens (DS 220BT). Circular dichroism experiments were carried out using the Jasco J-815 spectrometer using a rectangular quartz cell of 1 cm path length. Fluorescence spectra were recorded at room temperature using an Agilent spectrophotometer. Electronic spectra of the compounds were obtained on a Labman UV–Visible spectrophotometer. The mass spectral analysis of all the heterocyclic derivatives was done by LC MS/MS Waters.

4

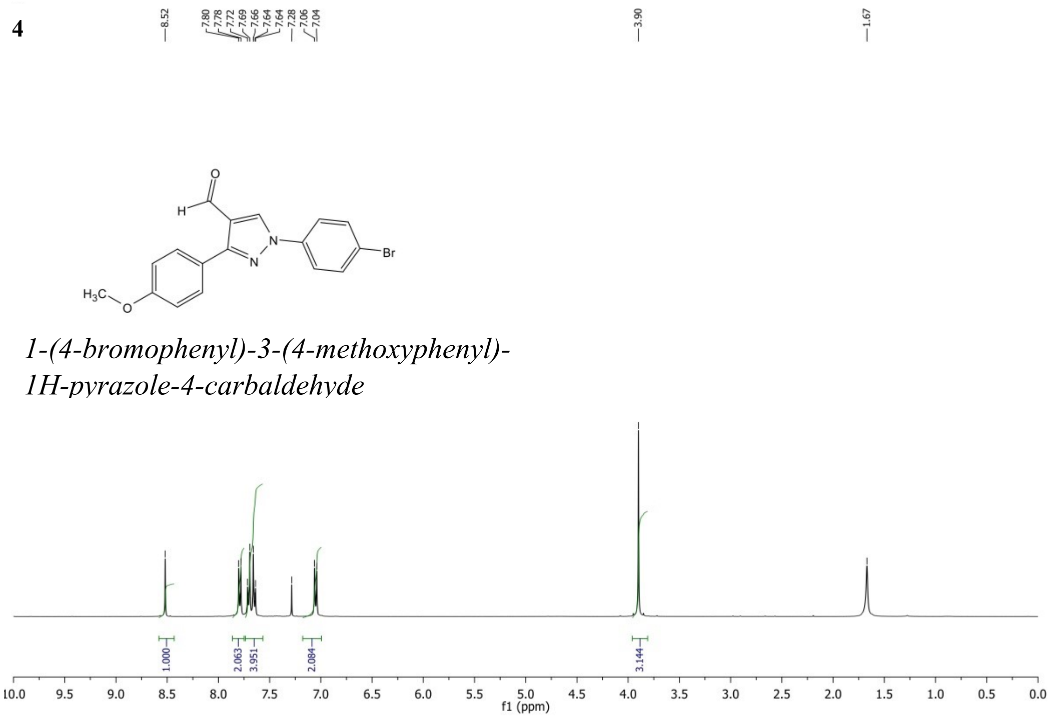

^{1}H-NMR spectra at 400 MHz in $CDCl_3$ of compound 4

4

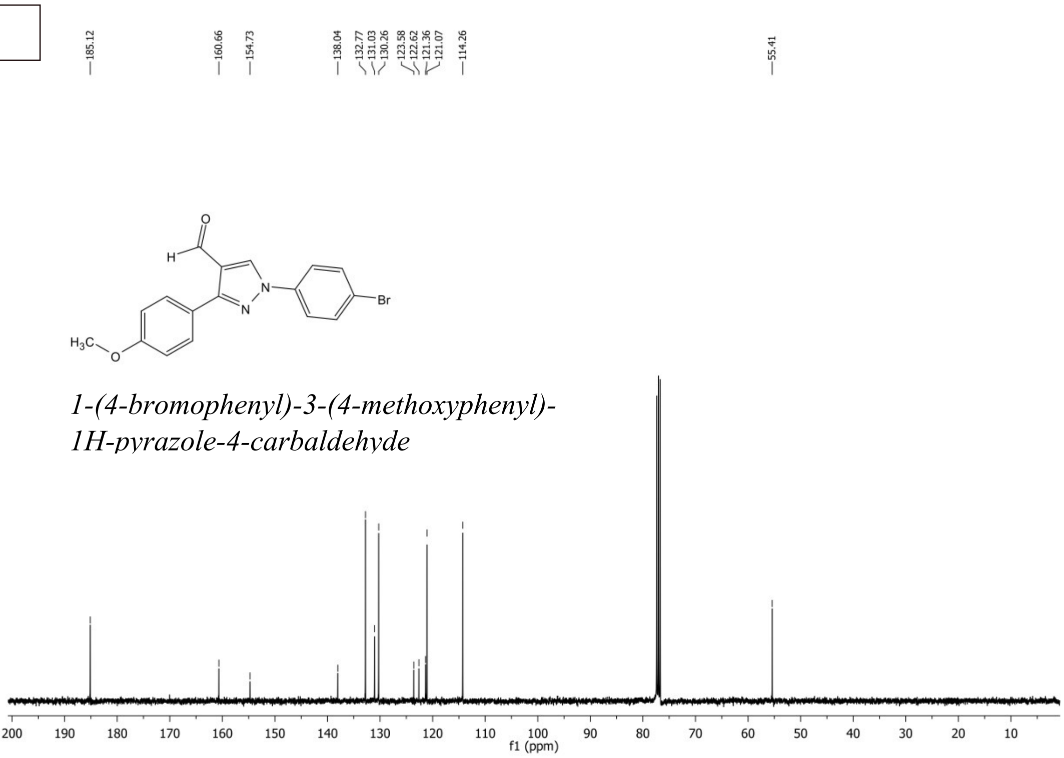

^{13}C-NMR spectra at 100 MHz in $CDCl_3$ of compound 4

5a

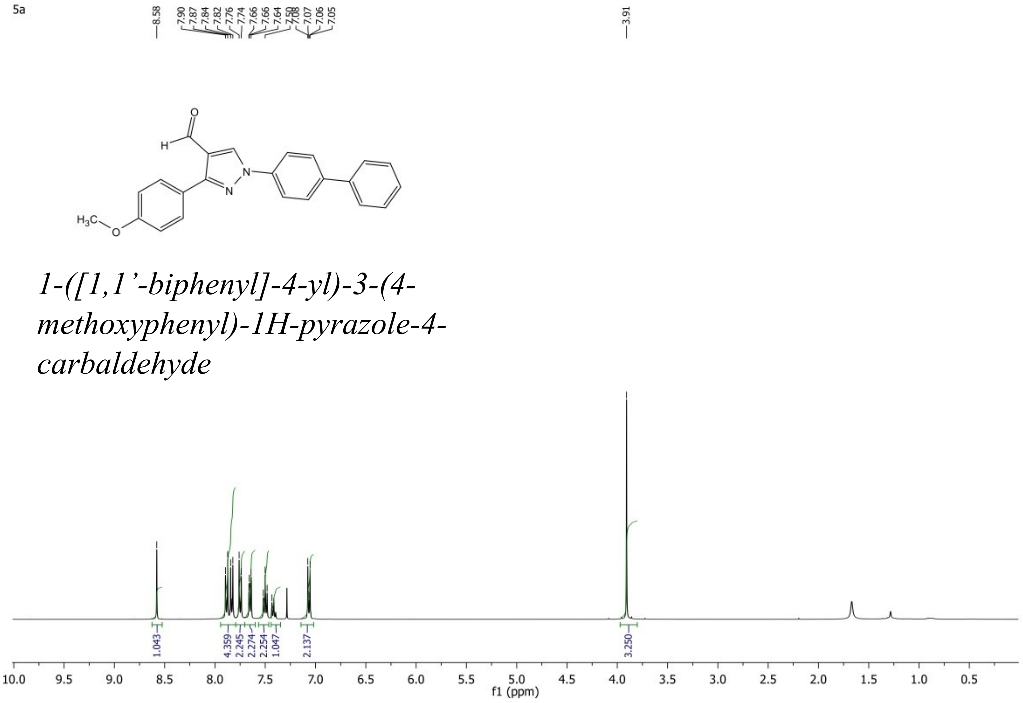

^{1}H-NMR spectra at 400 MHz in $CDCl_3$ of compound 5a

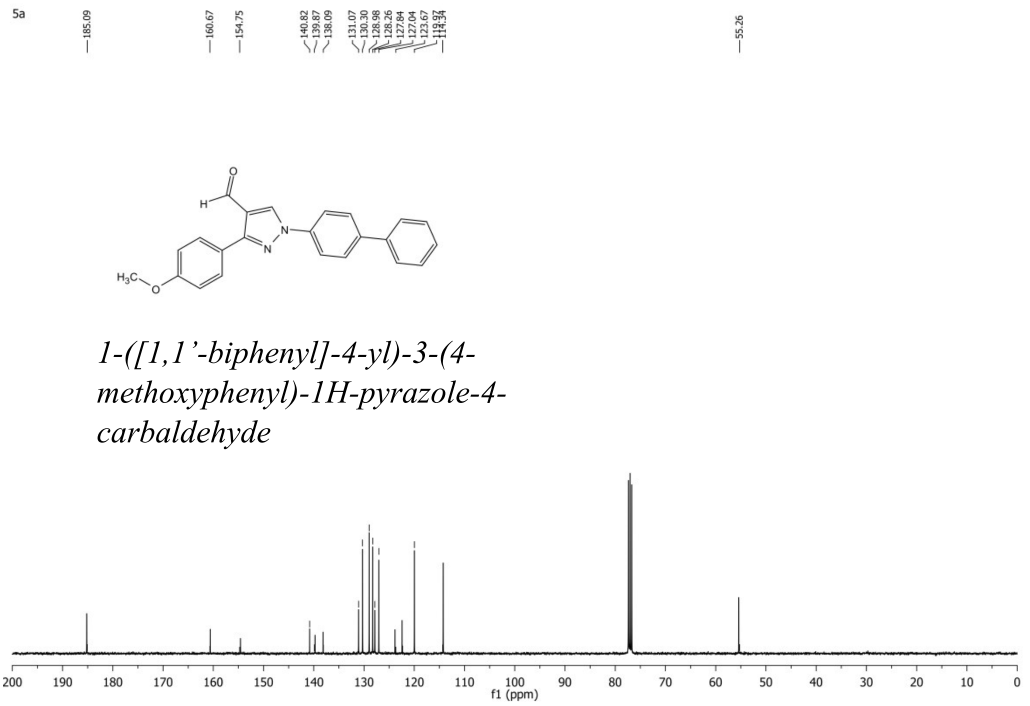

^{13}C-NMR spectra at 100 MHz in $CDCl_3$ of compound 5a

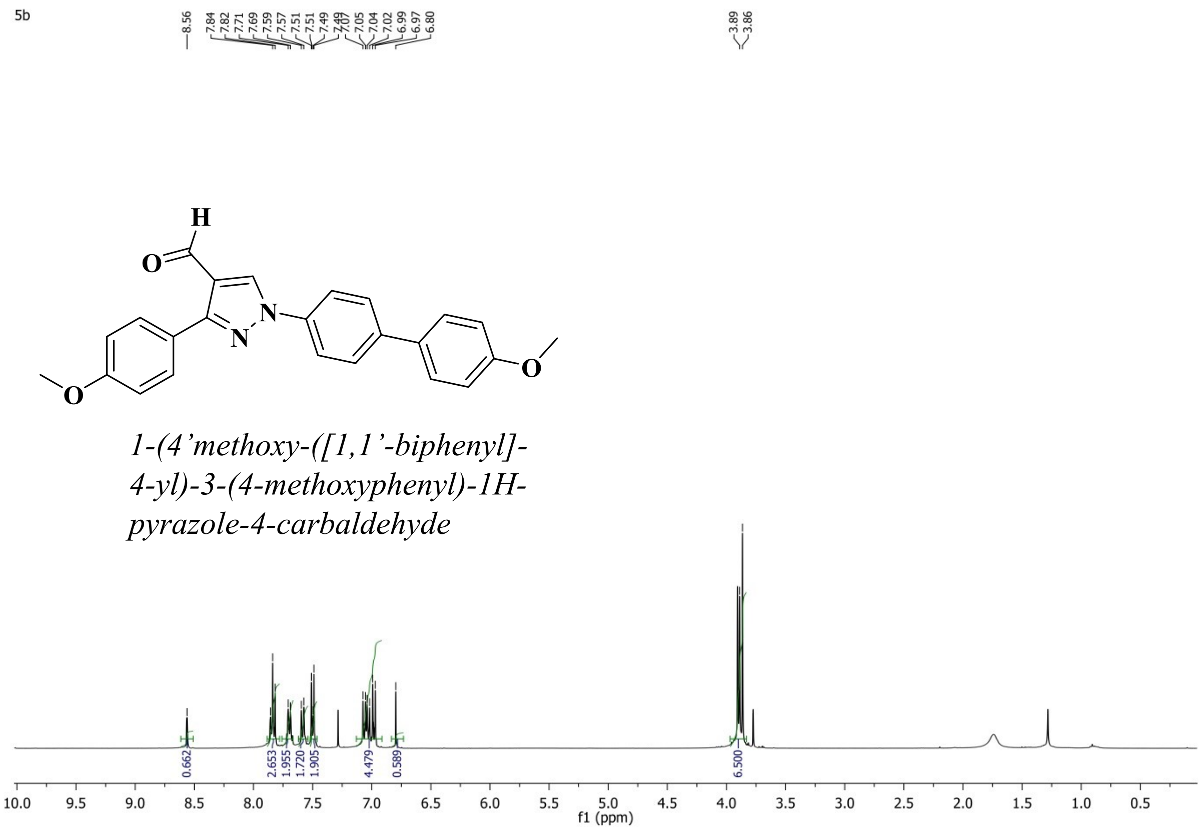

^{1}H-NMR spectra at 400 MHz in $CDCl_3$ of compound 5b

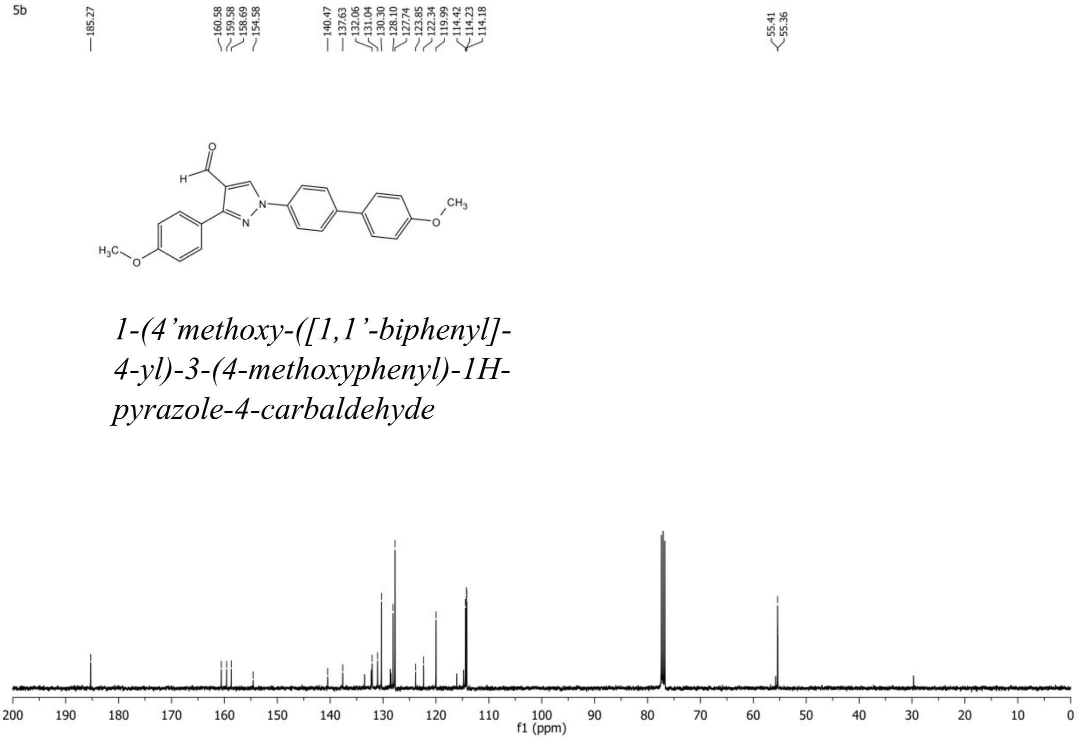

^{13}C-NMR spectra at 100 MHz in $CDCl_3$ of compound 5b

6a

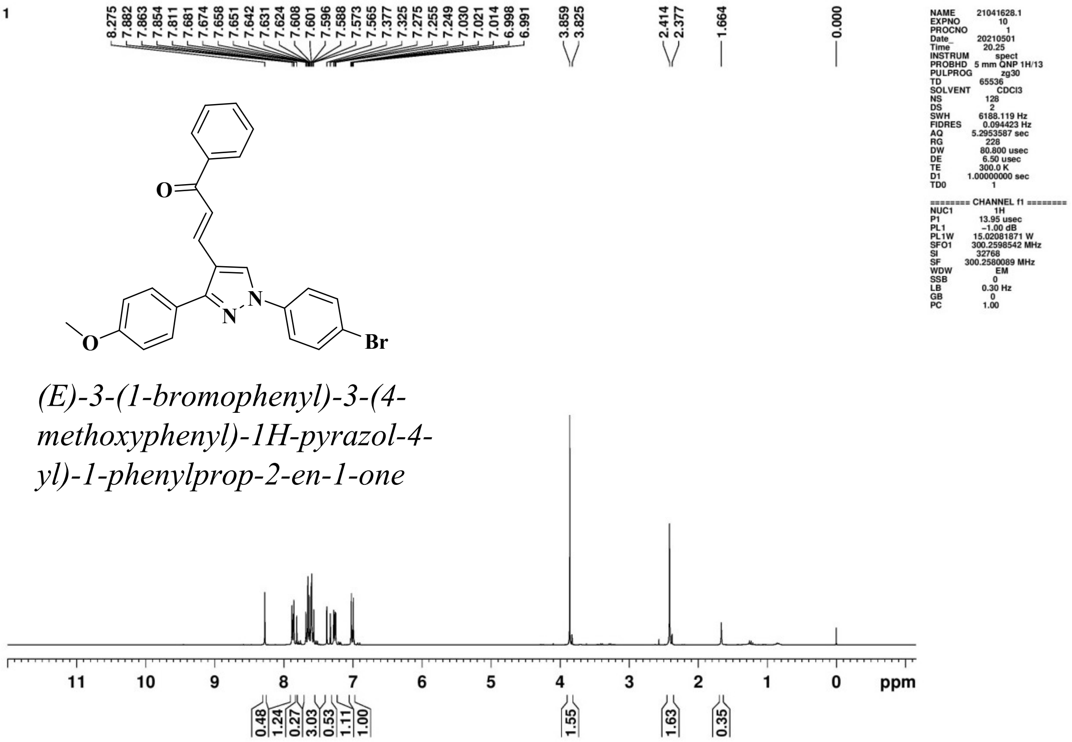

^{1}H-NMR spectra at 400 MHz in $CDCl_3$ of compound 6a

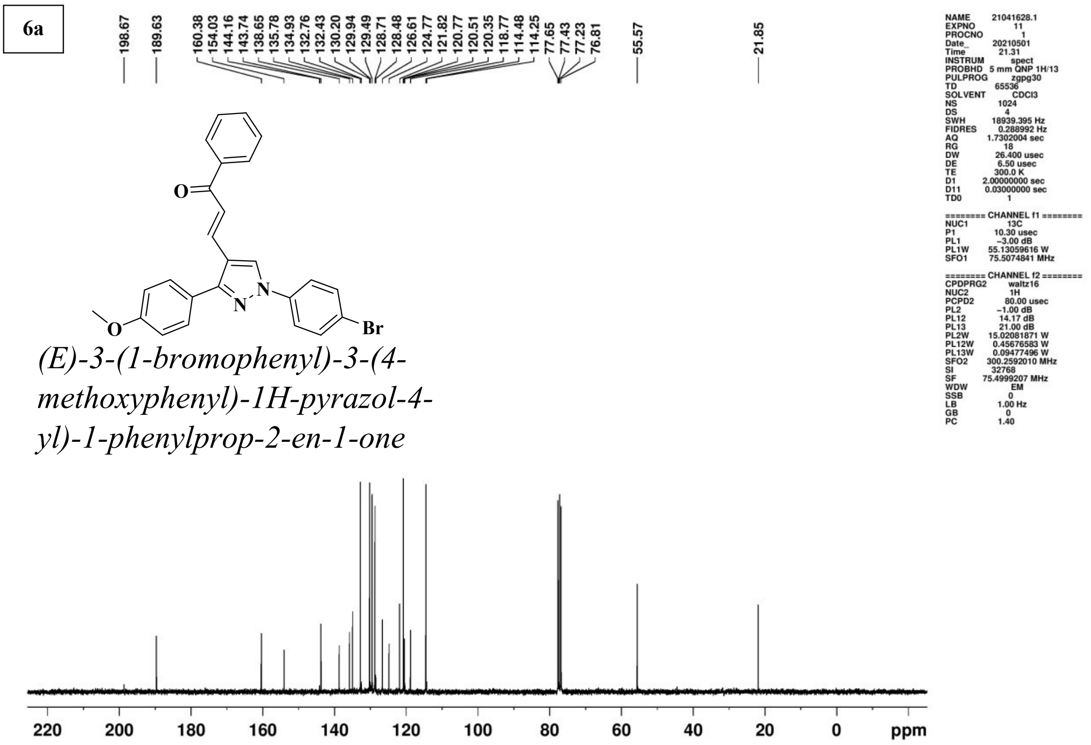

^{13}C-NMR spectra at 75 MHz in $CDCl_3$ of compound 6a

6b

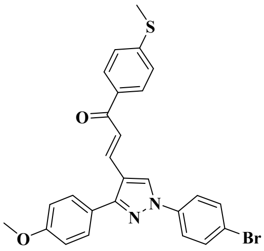

(E)-3-(1-(4-bromophenyl)-3-(4-methoxyphenyl)-1H-pyrazol-4-yl)-1-(4-(methylthio)phenyl)

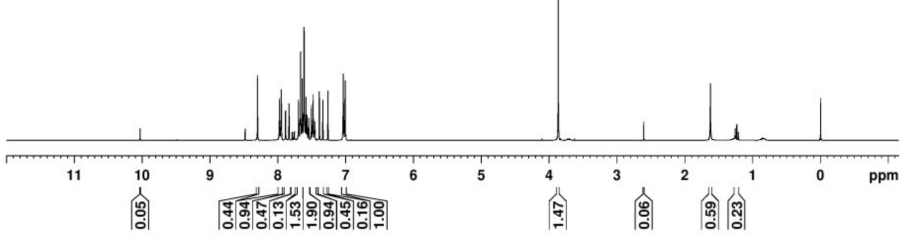

^{1}H-NMR spectra at 300 MHz in $CDCl_3$ of compound 6b

6b

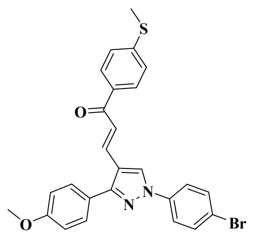

(E)-3-(1-bromophenyl)-3-(4-methoxyphenyl)-1H-pyrazol-4-yl)-1-phenylprop-2-en-1-one

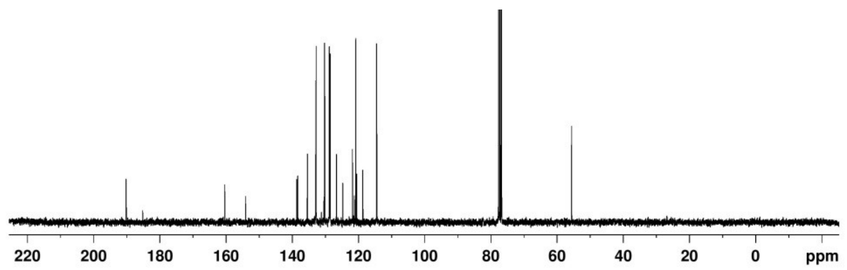

^{13}C-NMR spectra at 75 MHz in $CDCl_3$ of compound 6b

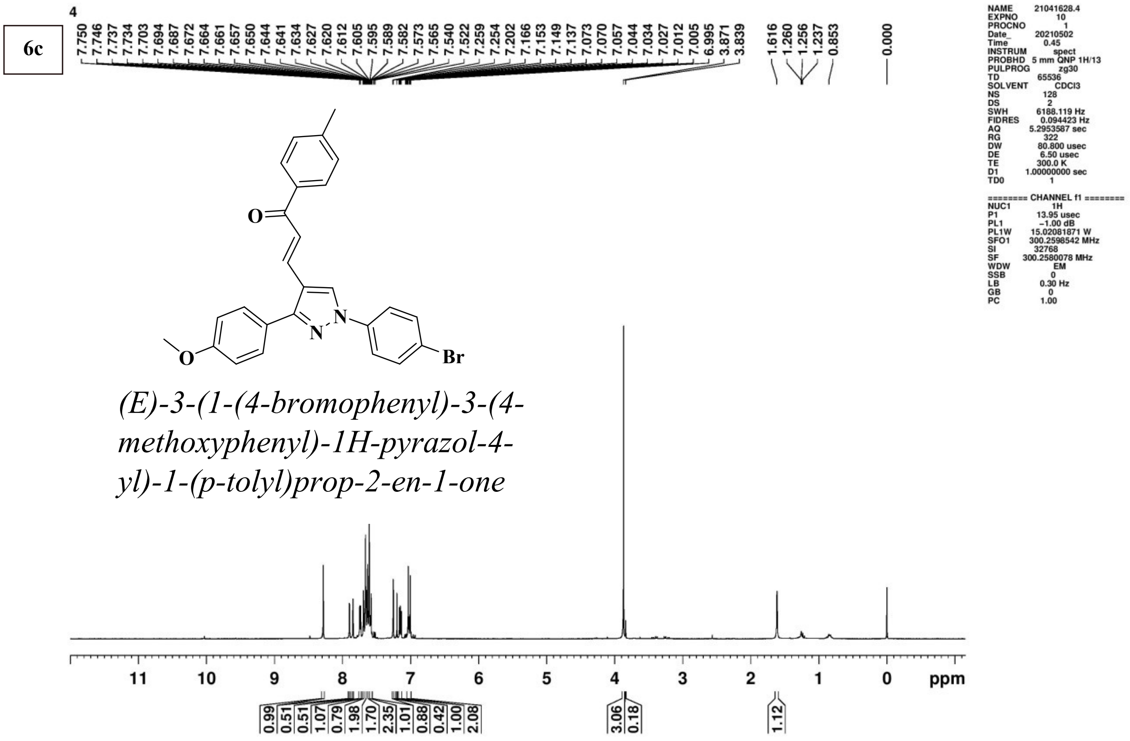

^{1}H-NMR spectra at 300 MHz in $CDCl_3$ of compound 6c

6c

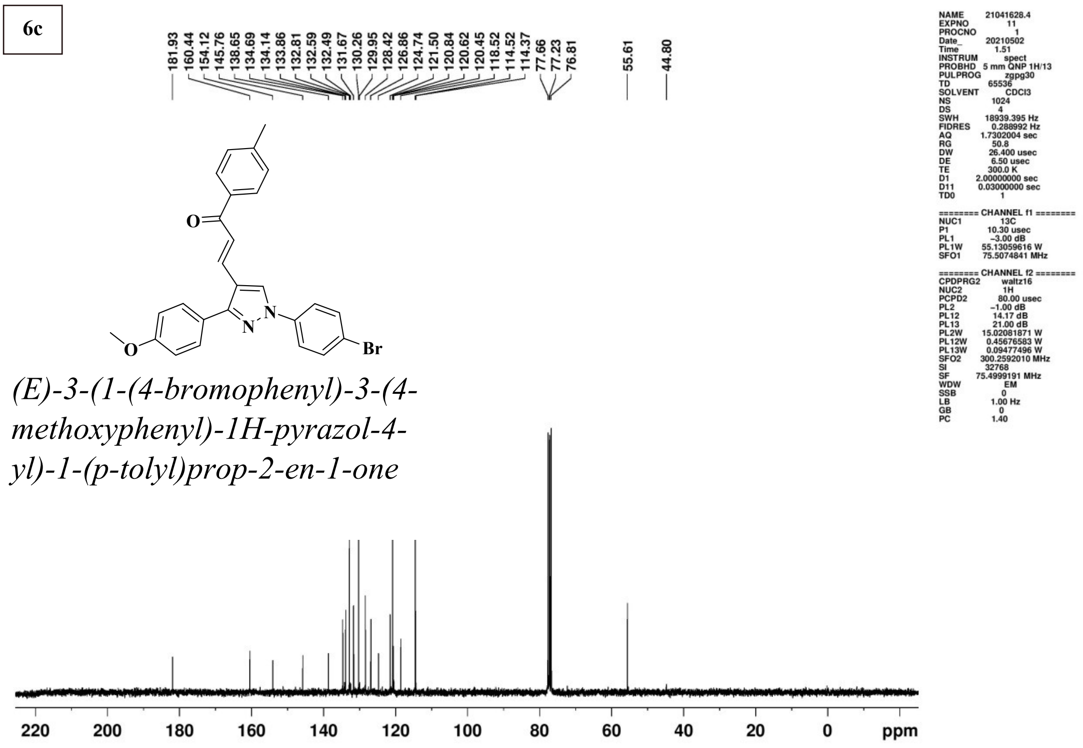

(E)-3-(1-(4-bromophenyl)-3-(4-methoxyphenyl)-1H-pyrazol-4-yl)-1-(p-tolyl)prop-2-en-1-one

^{13}C-NMR spectra at 75 MHz in $CDCl_3$ of compound 6c

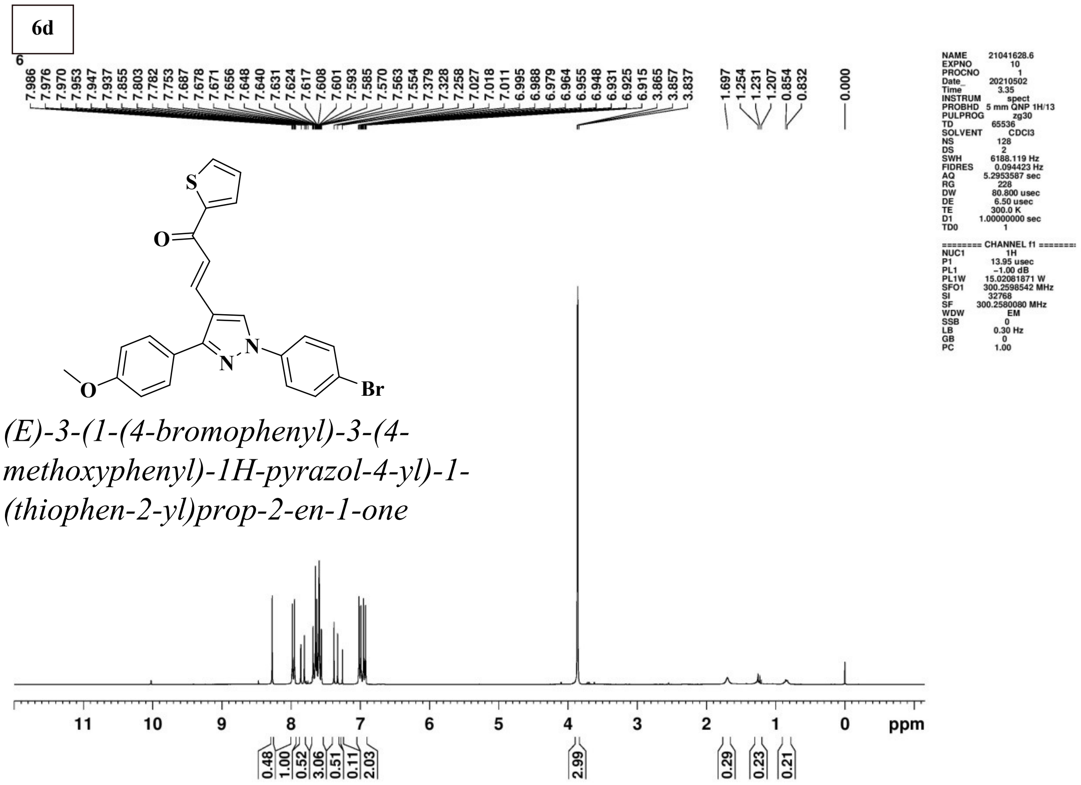

^{1}H-NMR spectra at 300 MHz in $CDCl_3$ of compound 6d

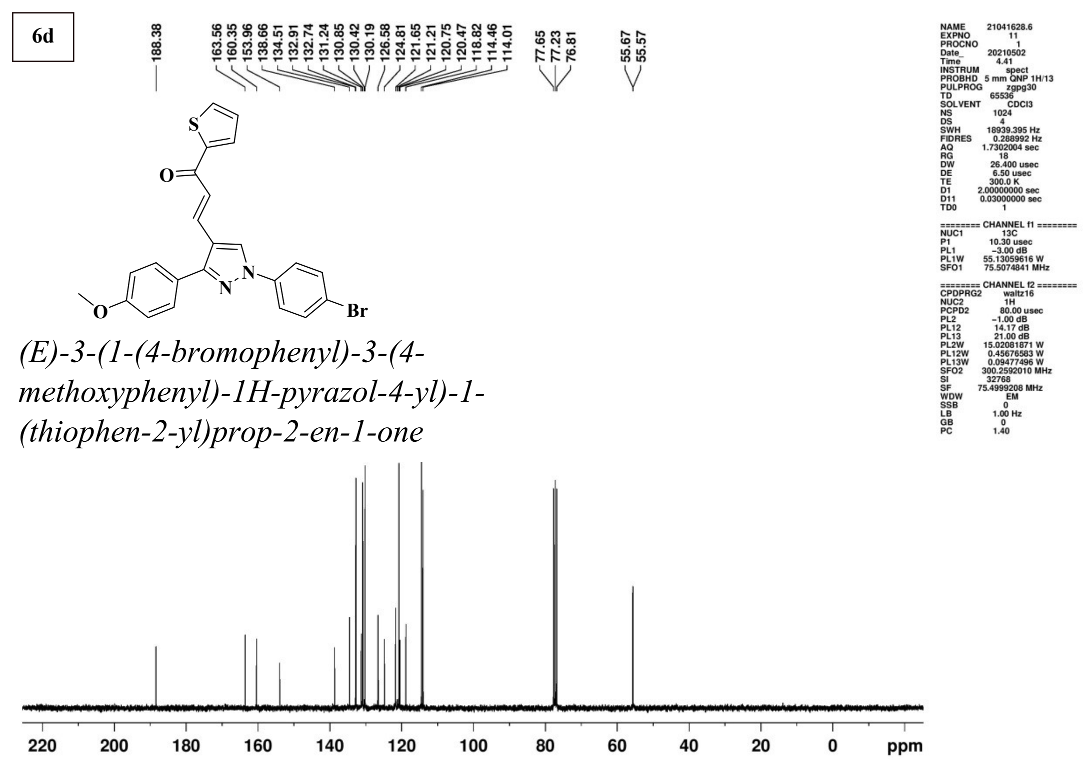

^{13}C-NMR spectra at 75 MHz in $CDCl_3$ of compound 6d

7a

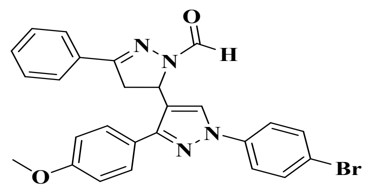

1'-(4-bromophenyl)-3'-(4-methoxyphenyl)-5-phenyl-3,4-dihydro-1'H,2H-[3,4'-bipyrazole]-2-carbaldehyde

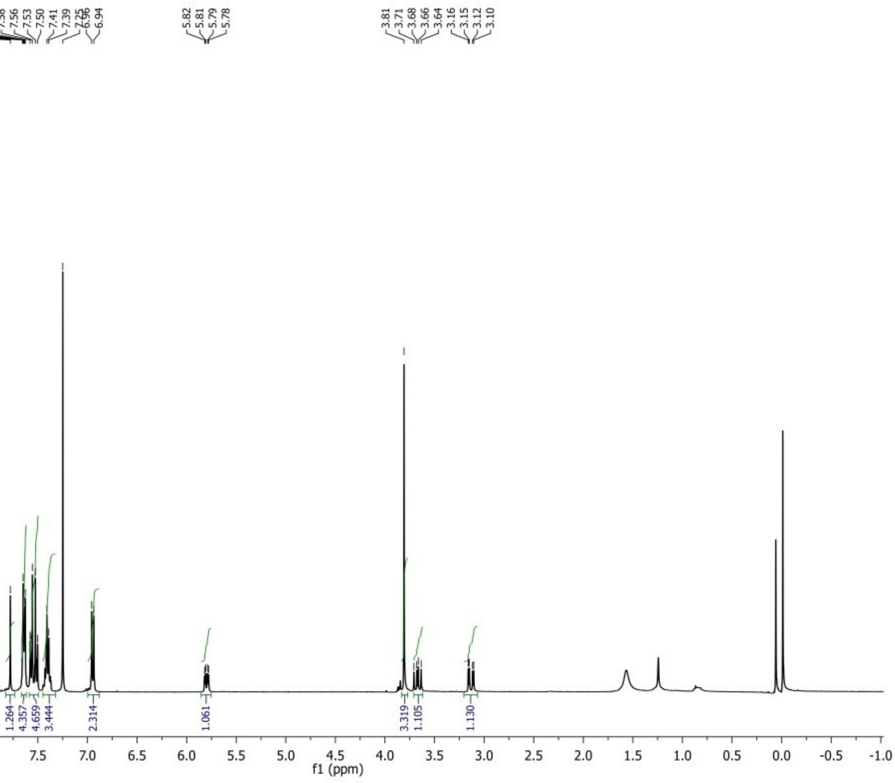

^{1}H-NMR spectra at 400 MHz in $CDCl_3$ of compound 7a

7a

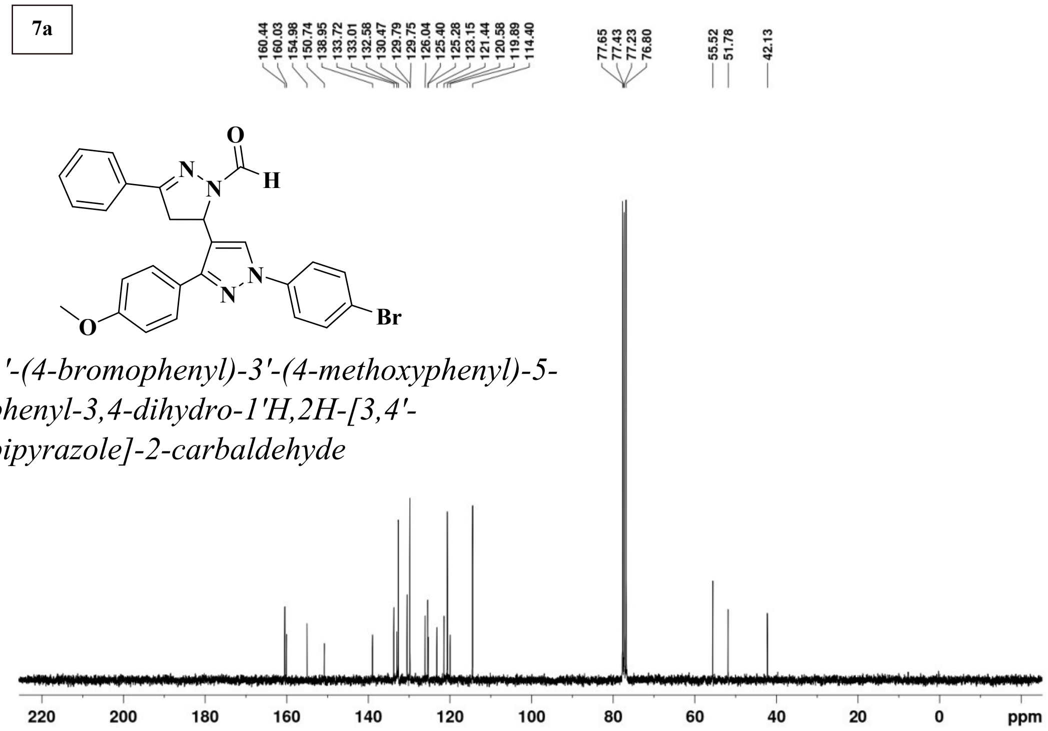

^{13}C-NMR spectra at 100 MHz in $CDCl_3$ of compound 7a

7b

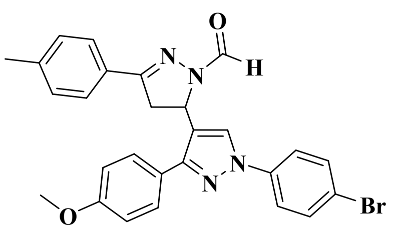

1'-(4-bromophenyl)-3'-(4-methoxyphenyl)-5-(p-tolyl)-3,4-dihydro-1'H,2H-[3,4'-bipyrazole]-2-carbaldehyde

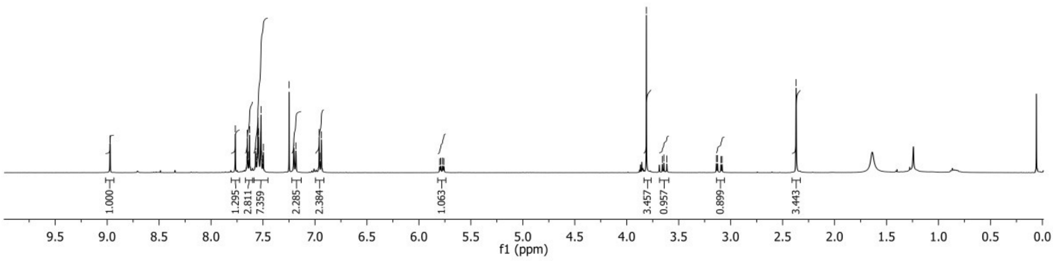

^{1}H-NMR spectra at 400 MHz in $CDCl_3$ of compound 7b

7b

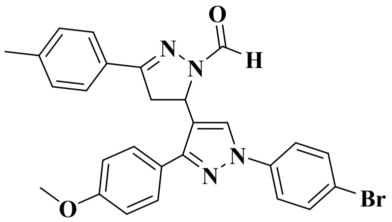

1'-(4-bromophenyl)-3'-(4-methoxyphenyl)-5-(p-tolyl)-3,4-dihydro-1'H,2H-[3,4'-bipyrazole]-2-carbaldehyde

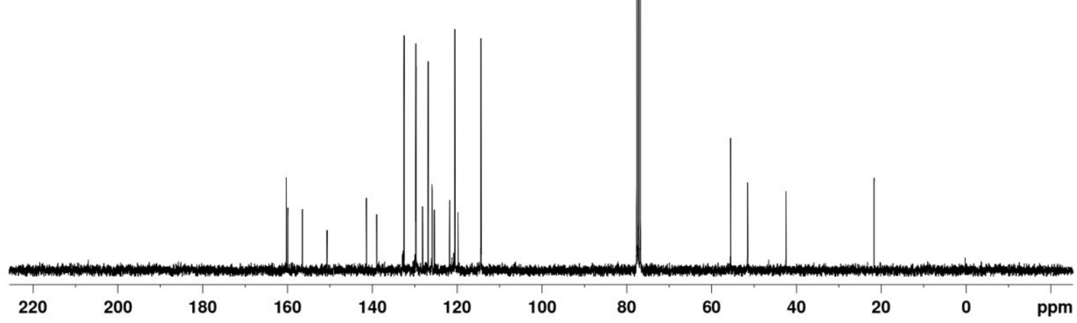

^{13}C-NMR spectra at 100 MHz in $CDCl_3$ of compound 7b

7c

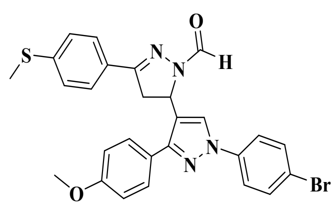

1'-(4-bromophenyl)-3'-(4-methoxyphenyl)-5-(4-(methylthio)phenyl)-3,4-dihydro-1'H,2H-[3,4'-bipyrazole]-2-carbaldehyde

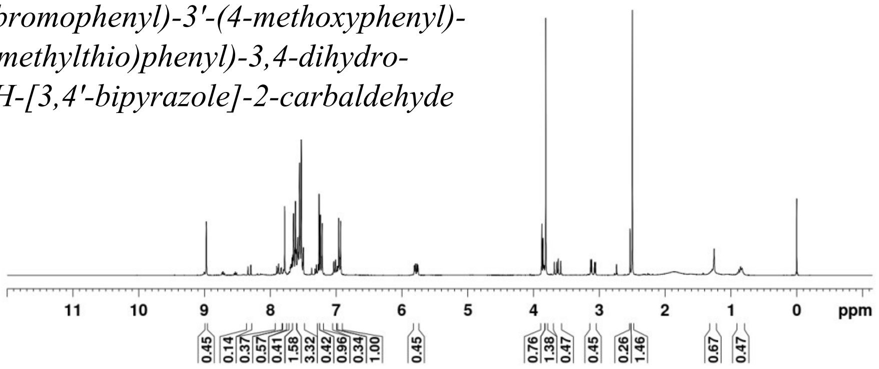

^{1}H-NMR spectra at 300 MHz in $CDCl_3$ of compound 7c

7c

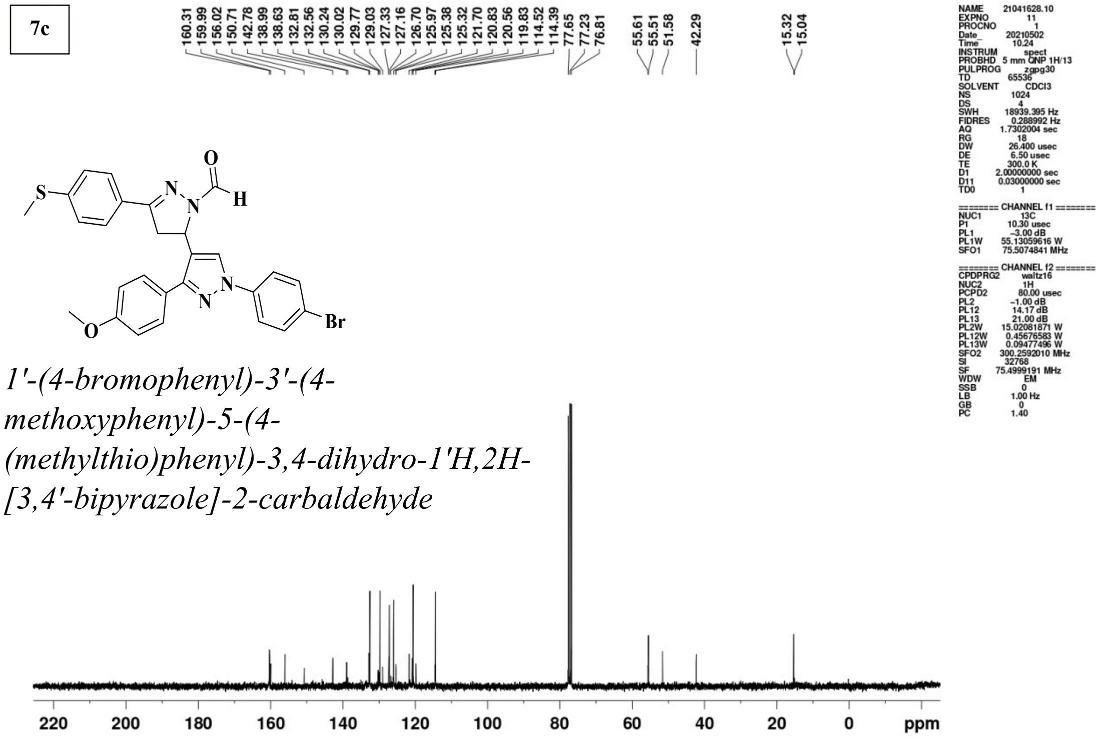

1'-(4-bromophenyl)-3'-(4-methoxyphenyl)-5-(4-(methylthio)phenyl)-3,4-dihydro-1'H,2H-[3,4'-bipyrazole]-2-carbaldehyde

^{13}C-NMR spectra at 75 MHz in $CDCl_3$ of compound 7c

7d

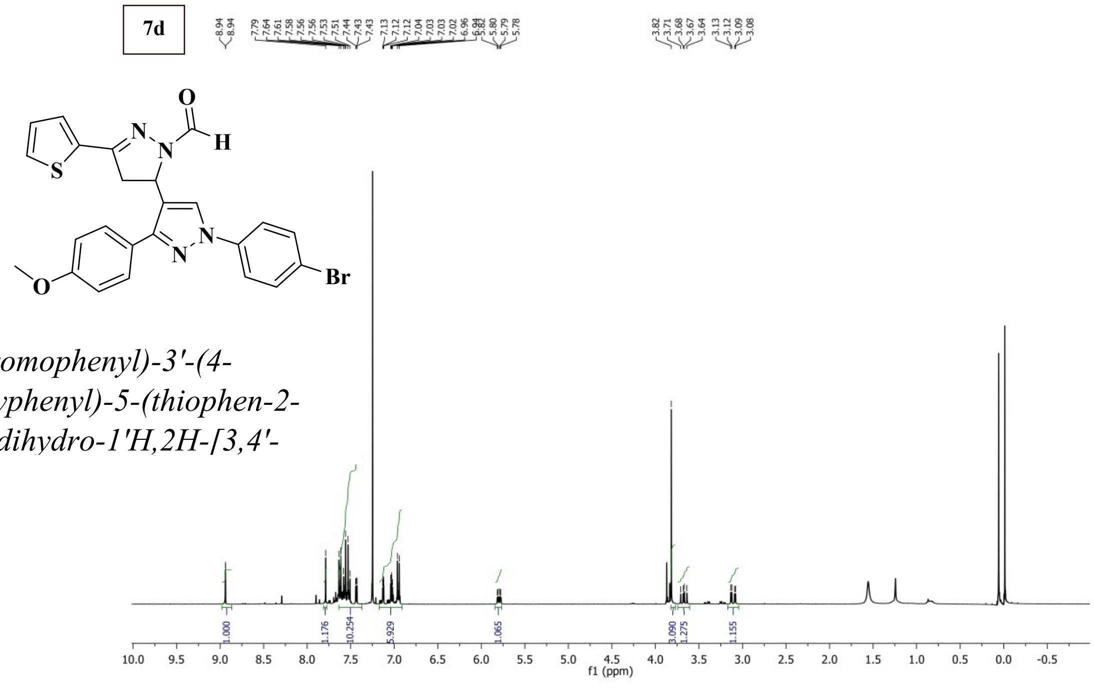

1'-(4-bromophenyl)-3'-(4-methoxyphenyl)-5-(thiophen-2-yl)-3,4-dihydro-1'H,2H-[3,4'-

^{1}H-NMR spectra at 400 MHz in $CDCl_3$ of compound 7d

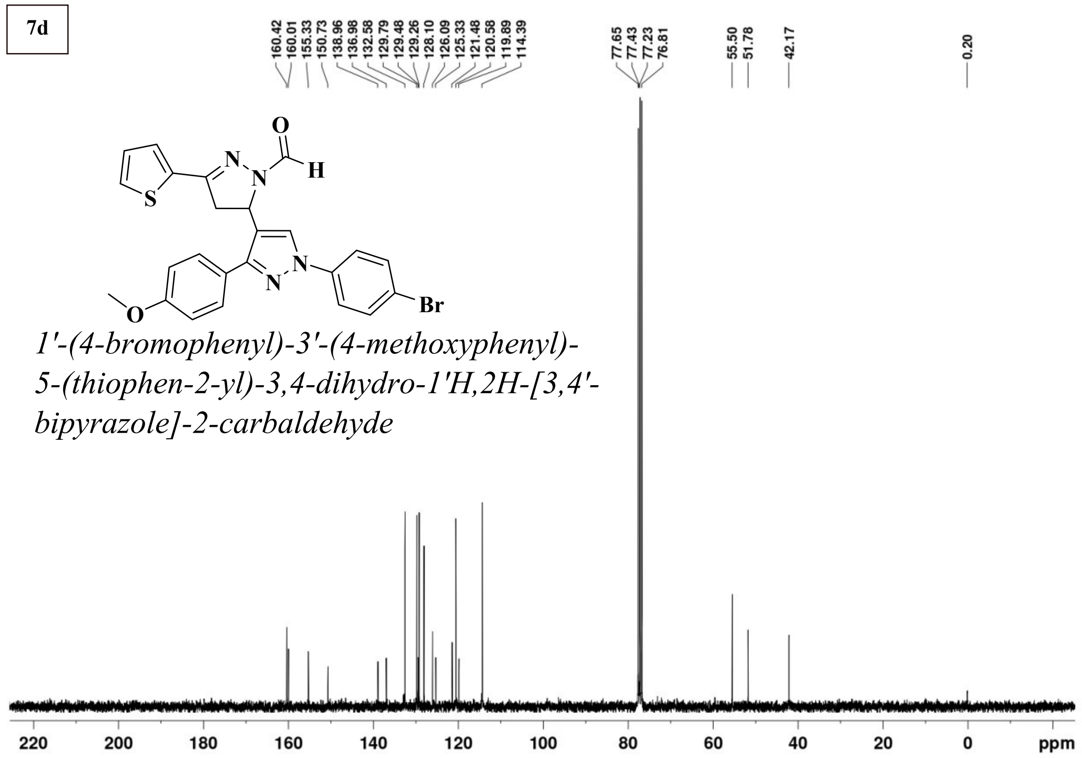

^{13}C-NMR spectra at 100 MHz in $CDCl_3$ of compound 7d

8a

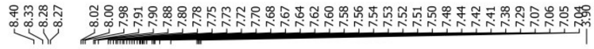

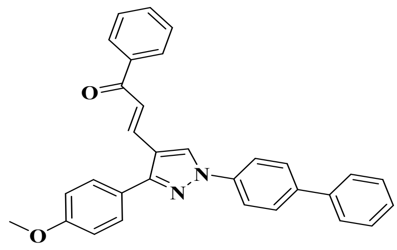

(E)-3-(1-([1,1'-biphenyl]-4-yl)-3-(4-methoxyphenyl)-1H-pyrazol-4-yl)-1-phenylprop-2-en-1-one

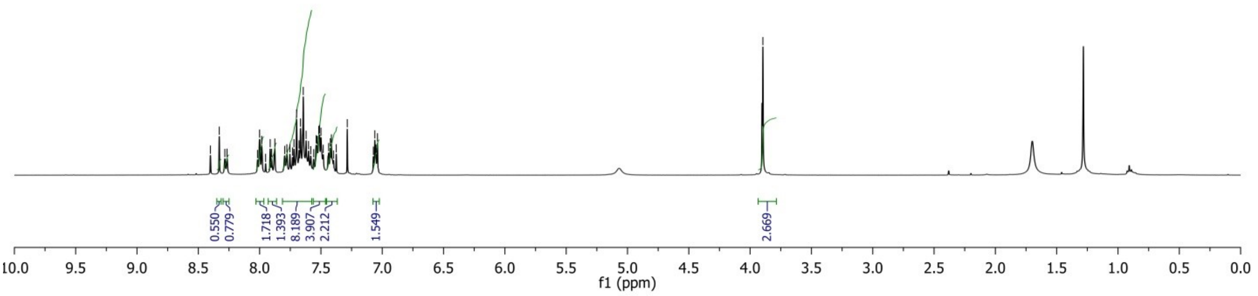

^{1}H-NMR spectra at 400 MHz in $CDCl_3$ of compound 8a

8a

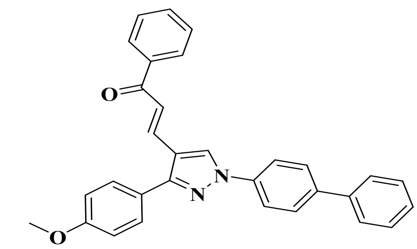

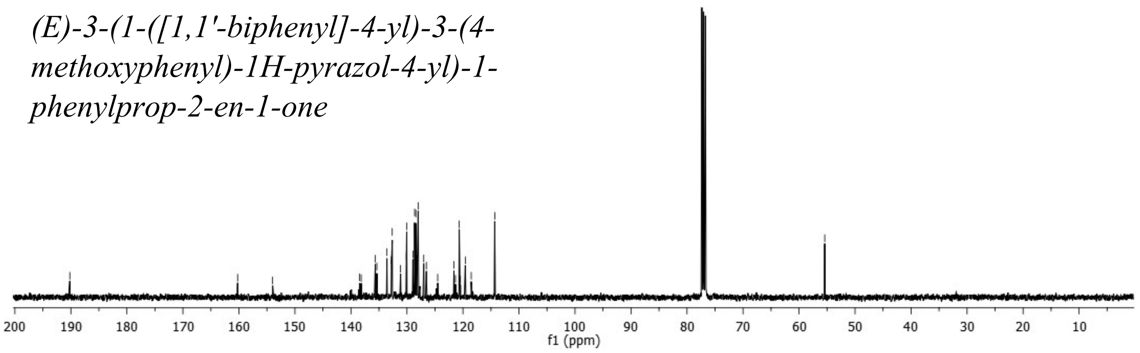

(E)-3-(1-([1,1'-biphenyl]-4-yl)-3-(4-methoxyphenyl)-1H-pyrazol-4-yl)-1-phenylprop-2-en-1-one

^{13}C-NMR spectra at 100 MHz in $CDCl_3$ of compound 8a

8b

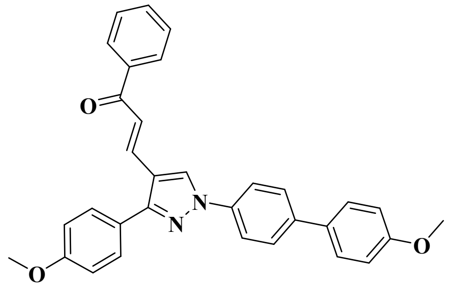

(E)-3-(1-(4'-methoxy-[1,1'-biphenyl]-4-yl)-3-(4-methoxyphenyl)-1H-pyrazol-4-yl)-1-phenylprop-2-en-1-one

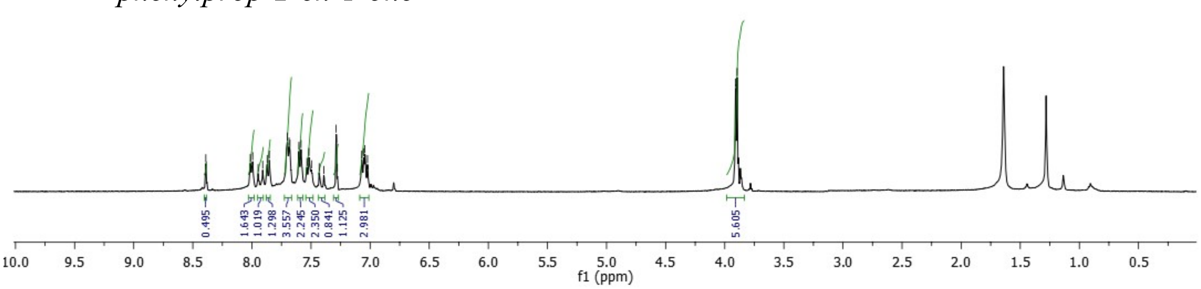

^{1}H-NMR spectra at 400 MHz in $CDCl_3$ of compound 8b

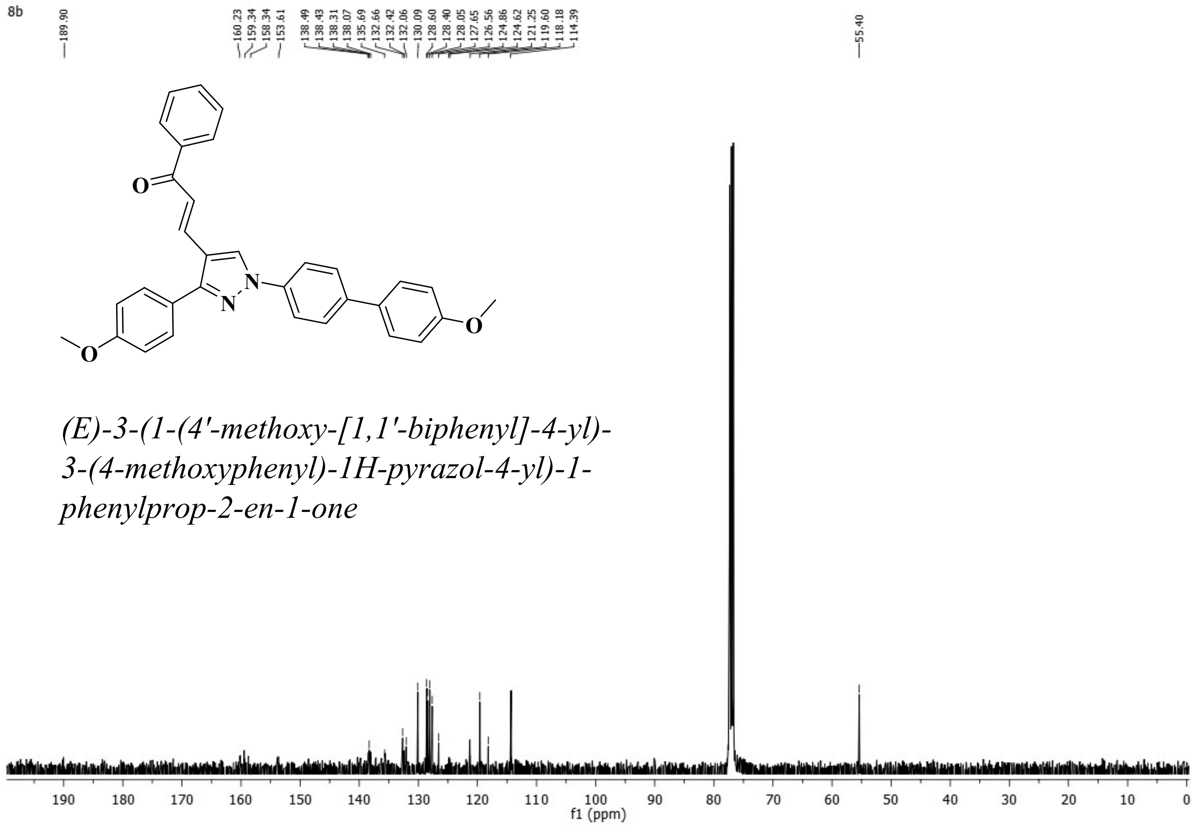

(E)-3-(1-(4'-methoxy-[1,1'-biphenyl]-4-yl)-3-(4-methoxyphenyl)-1H-pyrazol-4-yl)-1-phenylprop-2-en-1-one

^{13}C-NMR spectra at 100 MHz in $CDCl_3$ of compound 8b

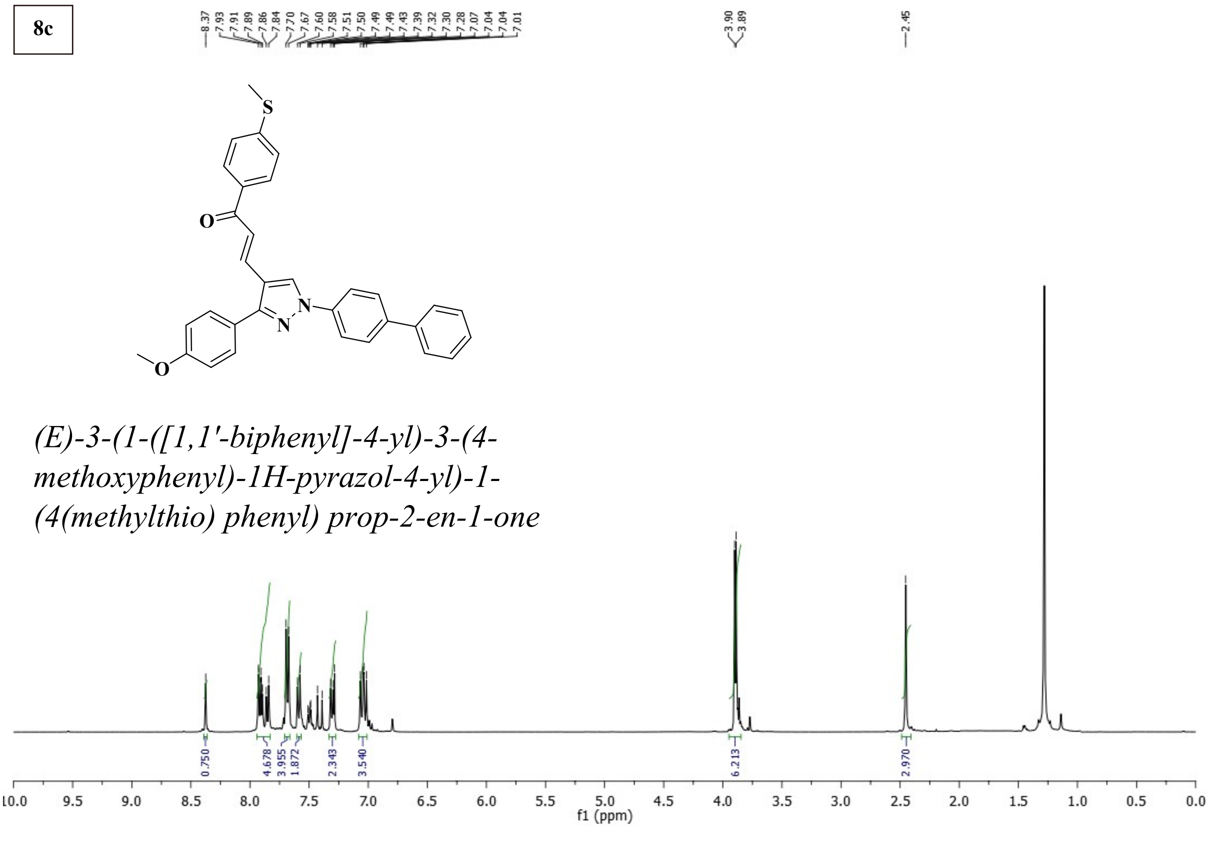

(E)-3-(1-([1,1'-biphenyl]-4-yl)-3-(4-methoxyphenyl)-1H-pyrazol-4-yl)-1-(4(methylthio) phenyl) prop-2-en-1-one

^{1}H-NMR spectra at 400 MHz in $CDCl_3$ of compound 8c

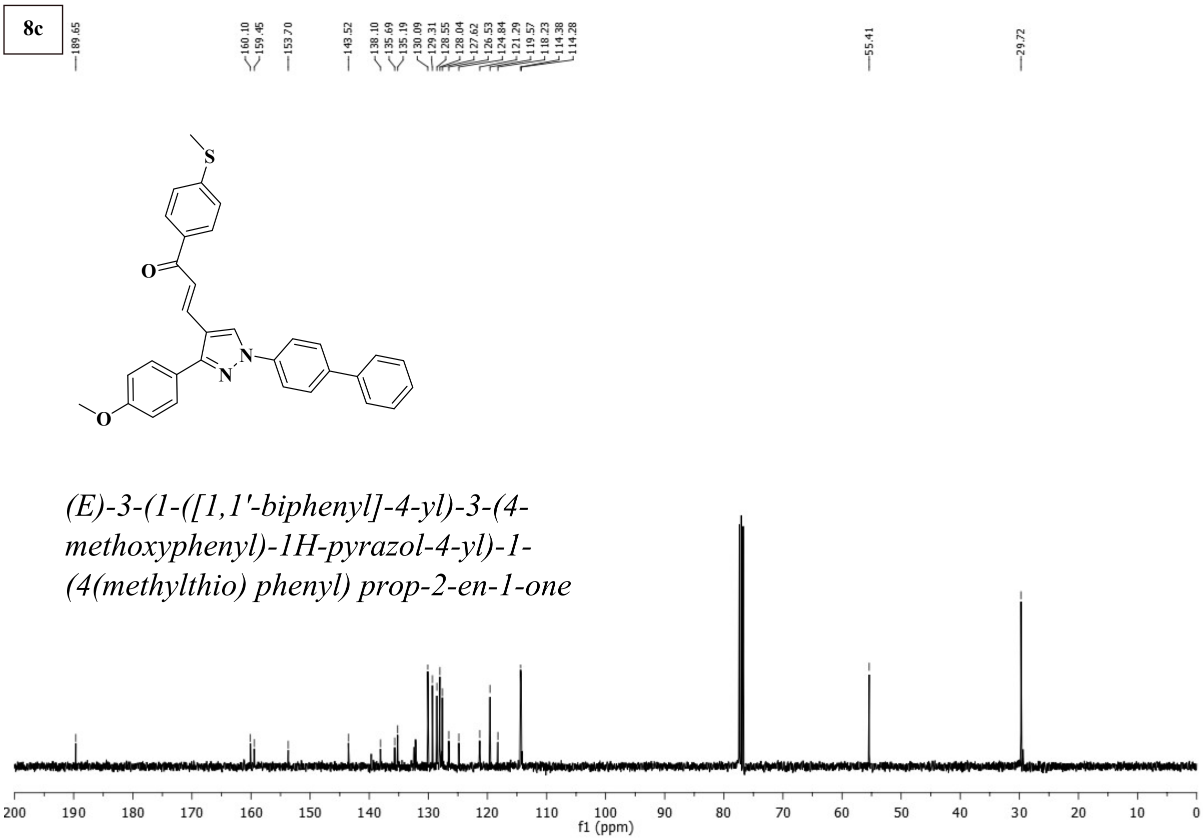

^{13}C-NMR spectra at 100 MHz in $CDCl_3$ of compound 8c

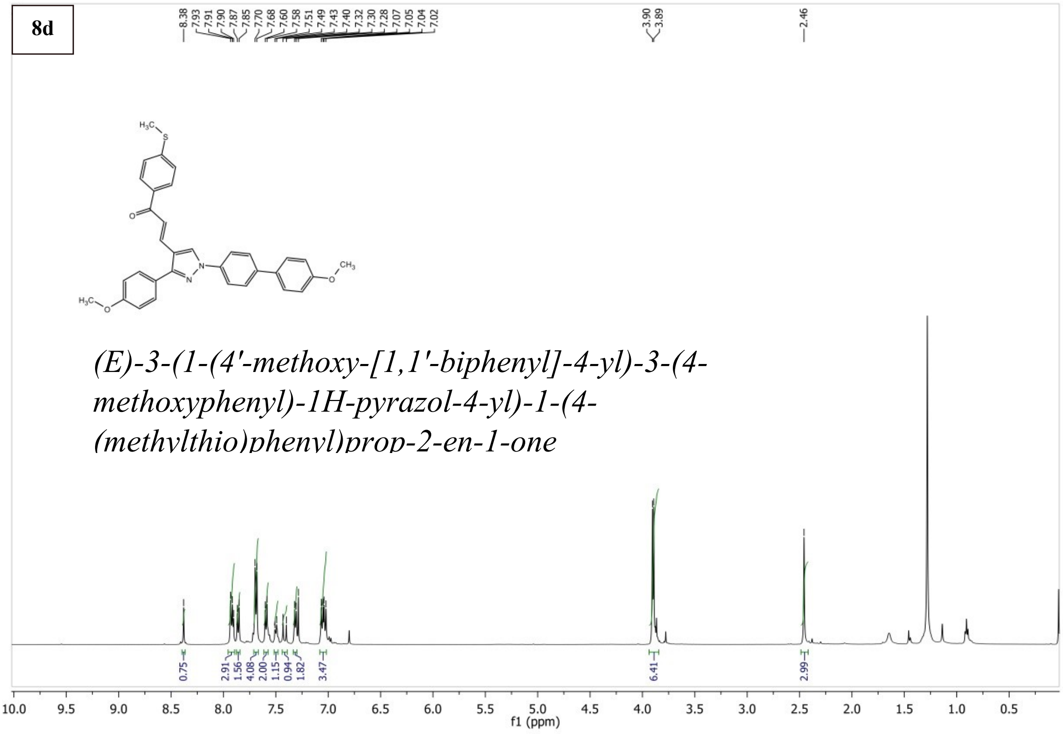

^{1}H-NMR spectra at 400 MHz in $CDCl_3$ of compound 8d

8d

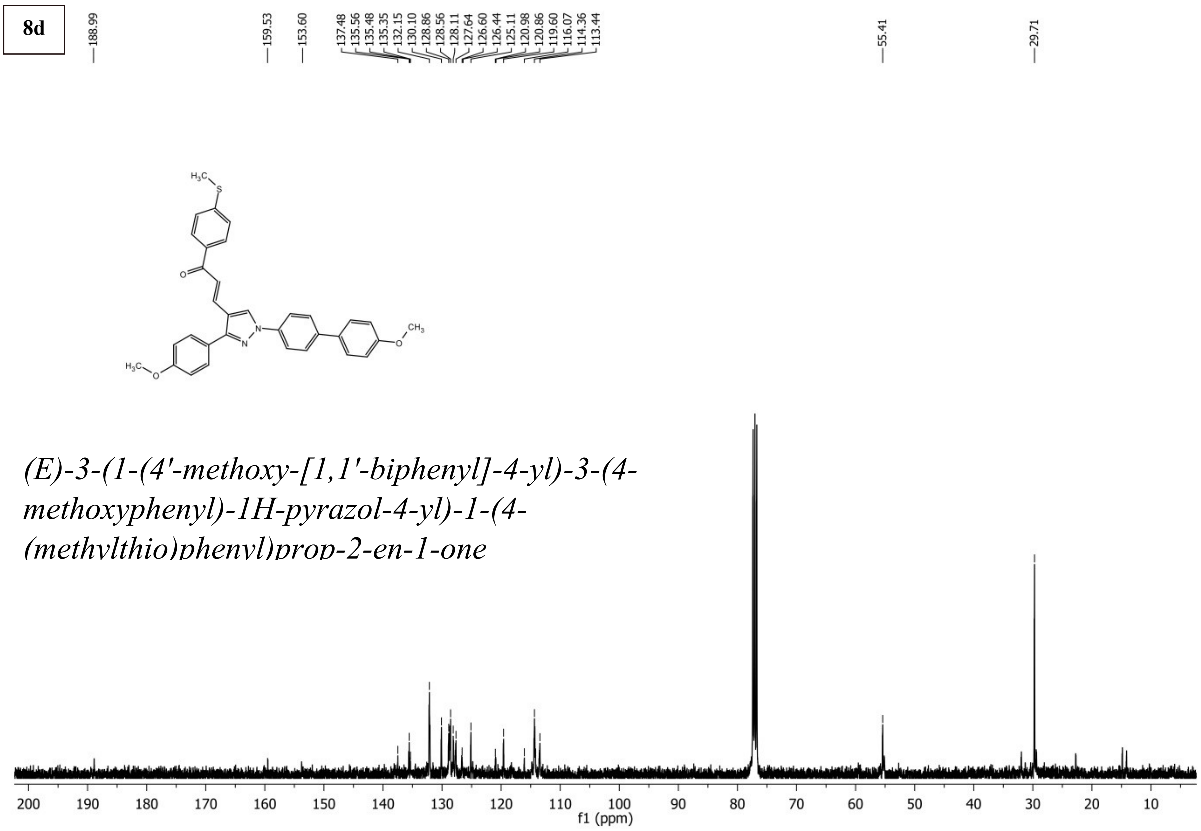

(E)-3-(1-(4'-methoxy-[1,1'-biphenyl]-4-yl)-3-(4-methoxyphenyl)-1H-pyrazol-4-yl)-1-(4-(methylthio)phenyl)prop-2-en-1-one

^{13}C-NMR spectra at 100 MHz in $CDCl_3$ of compound 8d

8e

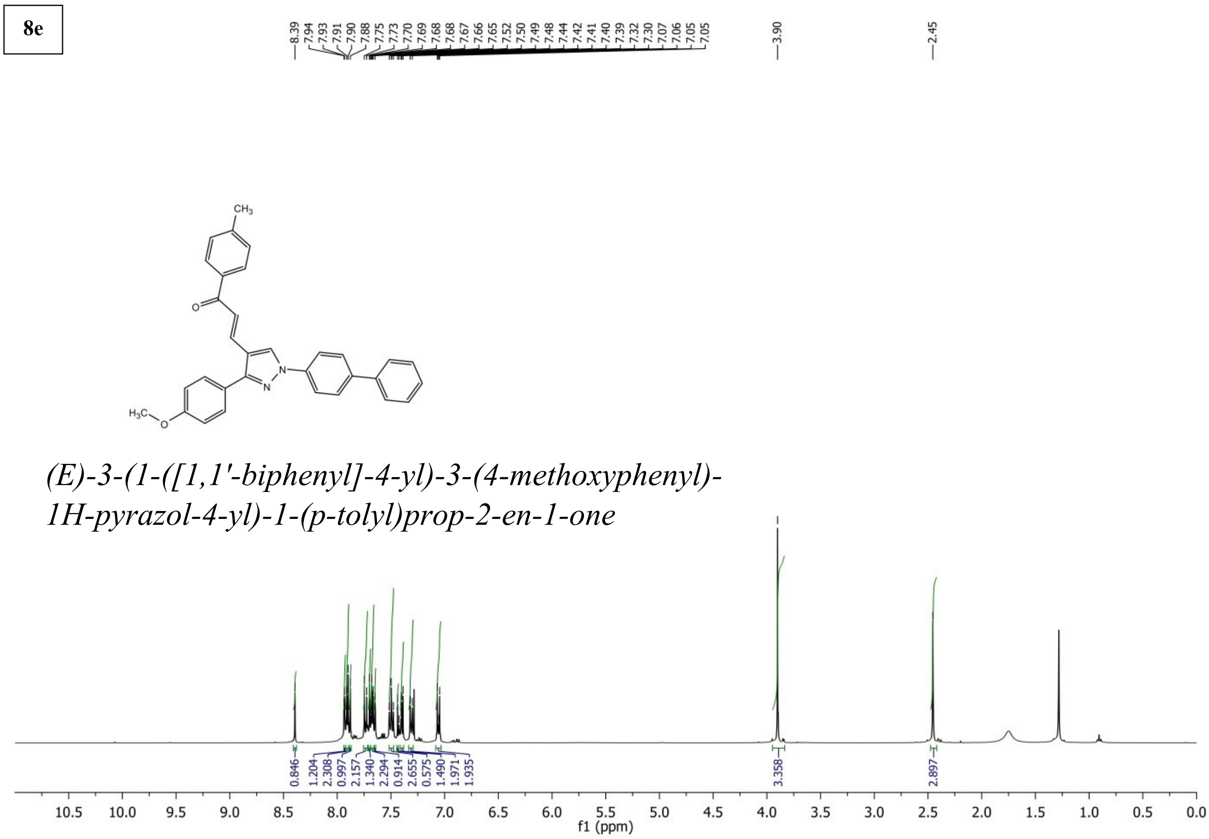

(E)-3-(1-([1,1'-biphenyl]-4-yl)-3-(4-methoxyphenyl)-1H-pyrazol-4-yl)-1-(p-tolyl)prop-2-en-1-one

^{1}H-NMR spectra at 400 MHz in $CDCl_3$ of compound 8e

8e

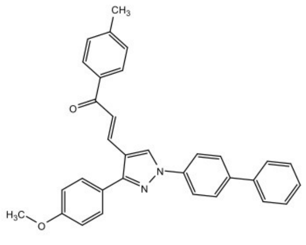

(E)-3-(1-([1,1'-biphenyl]-4-yl)-3-(4-methoxyphenyl)-1H-pyrazol-4-yl)-1-(p-tolyl)prop-2-en-1-one

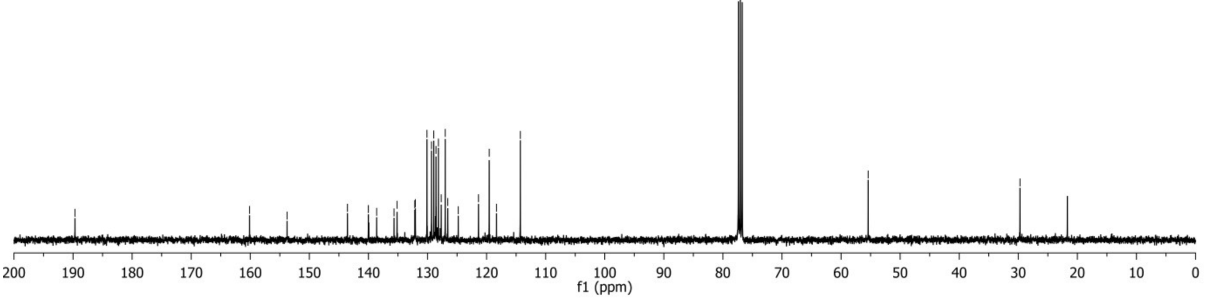

^{13}C-NMR spectra at 100 MHz in $CDCl_3$ of compound 8e

8f

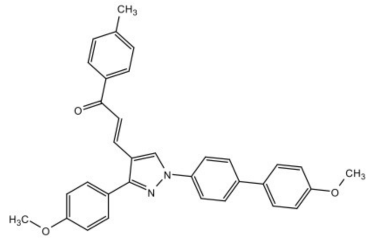

(E)-3-(1-(4'-methoxy-[1,1'-biphenyl]-4-yl)-3-(4-methoxyphenyl)-1H-pyrazol-4-yl)-1-(p-tolyl)prop-2-en-1-one

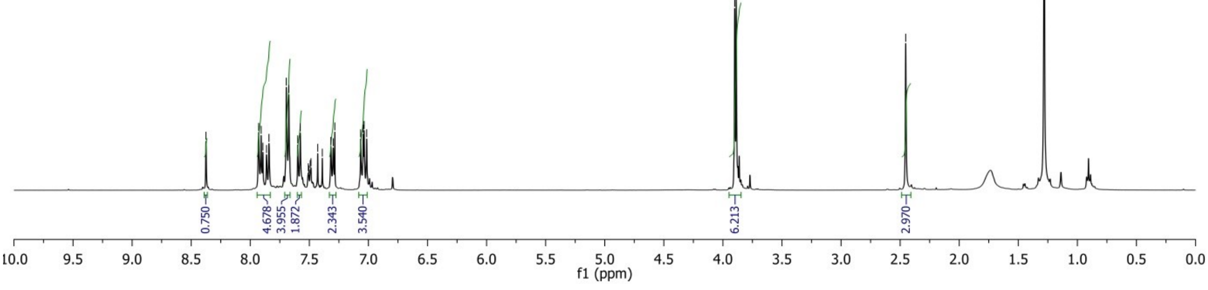

^{1}H-NMR spectra at 400 MHz in $CDCl_3$ of compound 8f

8f

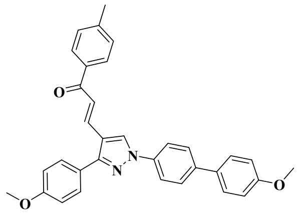

(E)-3-(1-(4'-methoxy-[1,1'-biphenyl]-4-yl)-3-(4-methoxyphenyl)-1H-pyrazol-4-yl)-1-(p-tolyl)prop-2-en-1-one

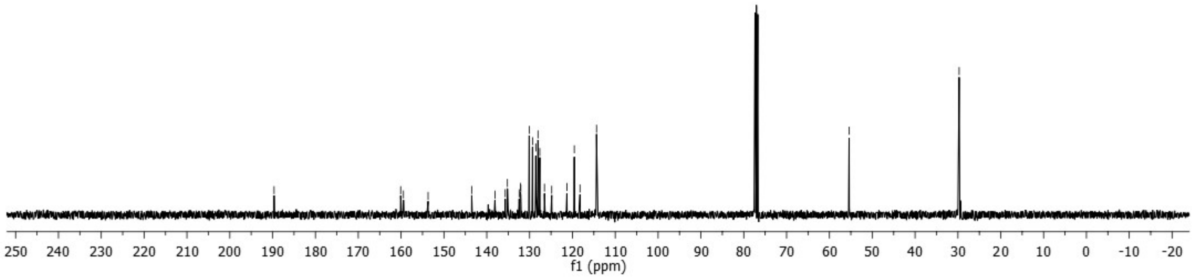

^{13}C-NMR spectra at 100 MHz in $CDCl_3$ of compound 8f

8g

8.32, 7.93, 7.90, 7.89, 7.88, 7.82, 7.80, 7.79, 7.78, 7.78, 7.77, 7.77, 7.70, 7.68, 7.67, 7.66, 7.64, 7.54, 7.52, 7.52, 7.50, 7.50, 7.48, 7.43, 7.41, 7.19, 7.19, 7.18, 7.17, 7.07, 7.07, 7.06, 7.05, 7.04, 3.90

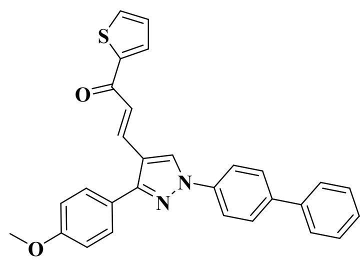

(E)-3-(1-([1,1'-biphenyl]-4-yl)-3-(4-methoxyphenyl)-1H-pyrazol-4-yl)-1-(thiophen-2-yl)prop-2-en-1-one

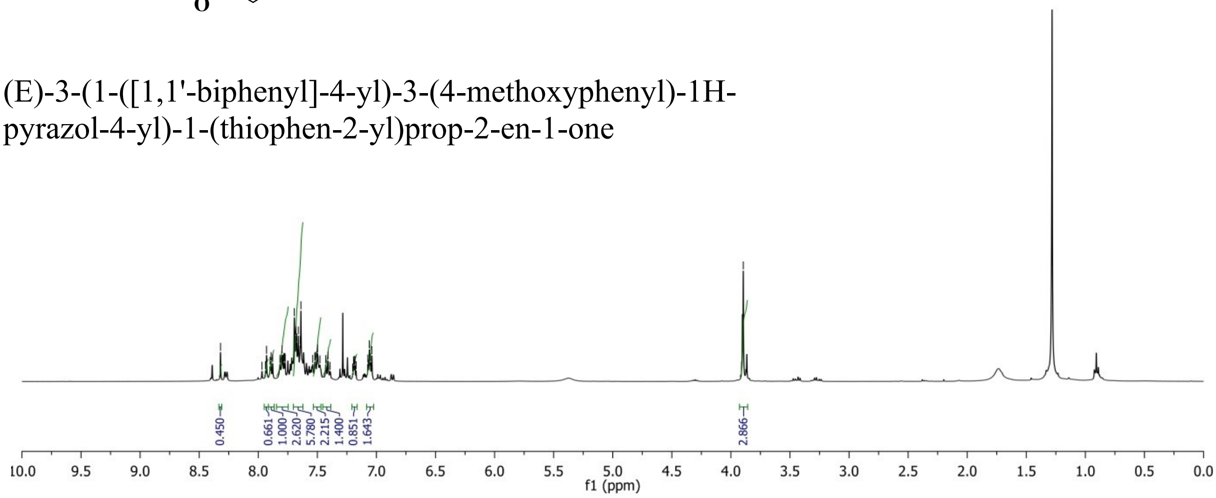

^{1}H-NMR spectra at 400 MHz in $CDCl_3$ of compound 8g

8g

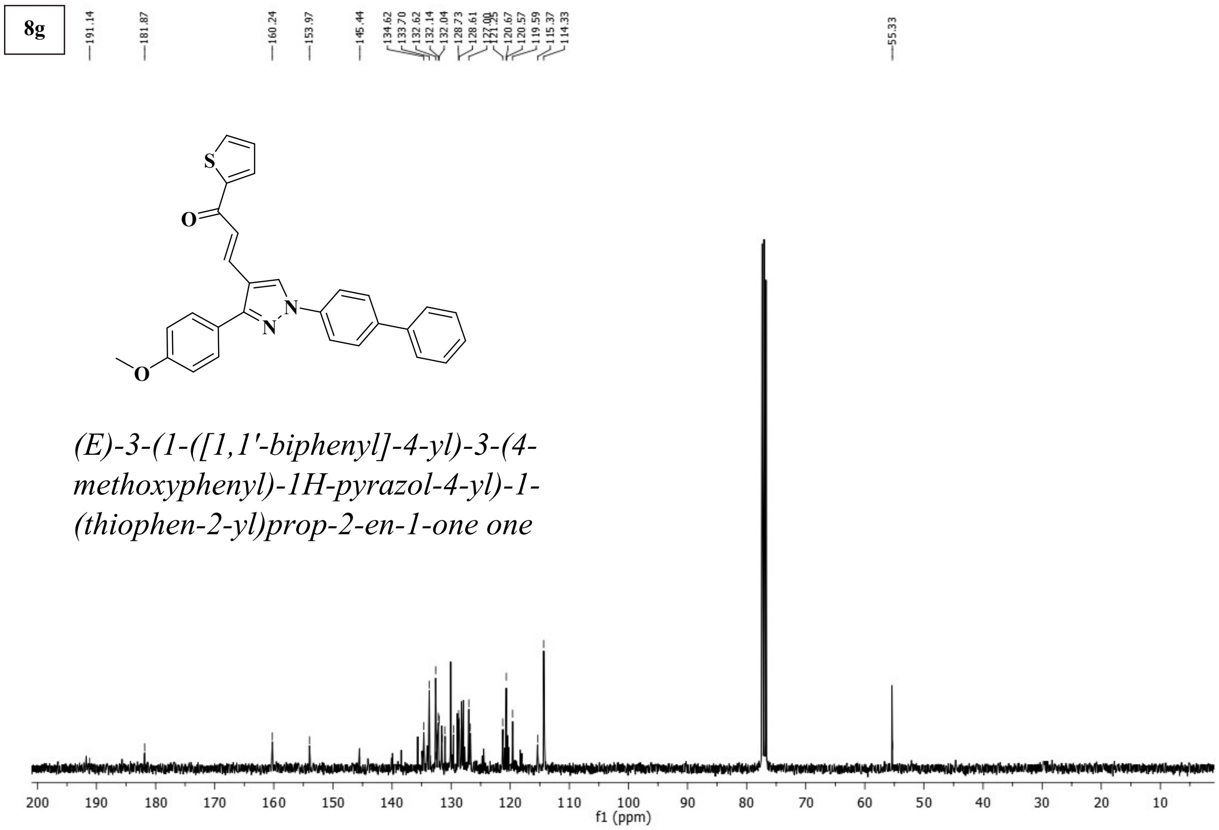

(E)-3-(1-([1,1'-biphenyl]-4-yl)-3-(4-methoxyphenyl)-1H-pyrazol-4-yl)-1-(thiophen-2-yl)prop-2-en-1-one one

^{13}C-NMR spectra at 100 MHz in $CDCl_3$ of compound 8g

8h

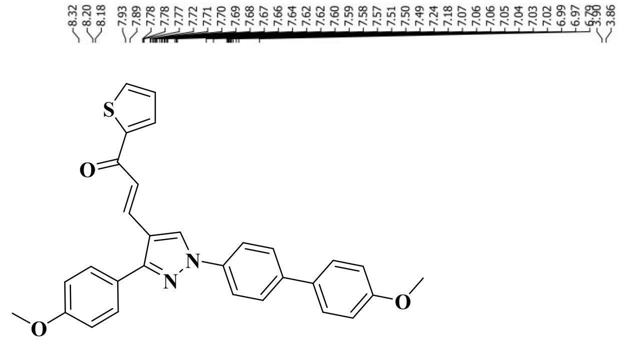

(E)-3-(1-(4'-methoxy-[1,1'-biphenyl]-4-yl)-3-(4-methoxyphenyl)-1H-pyrazol-4-yl)-1-(thiophen-2-yl)prop-2-en-1-one

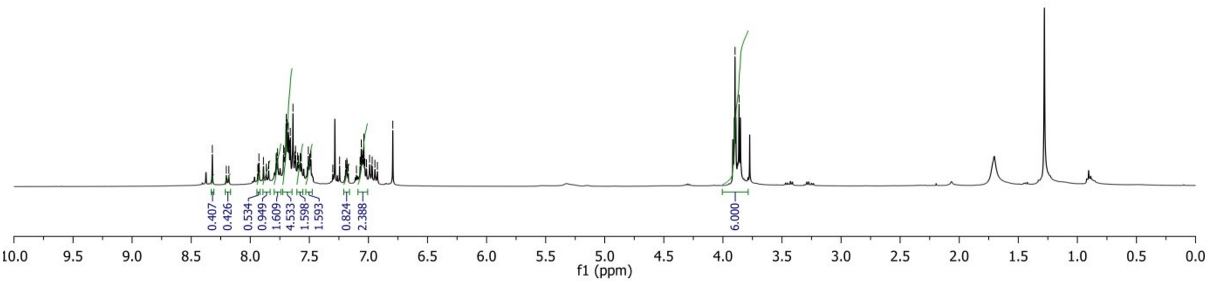

^{1}H-NMR spectra at 400 MHz in $CDCl_3$ of compound 8h

8h

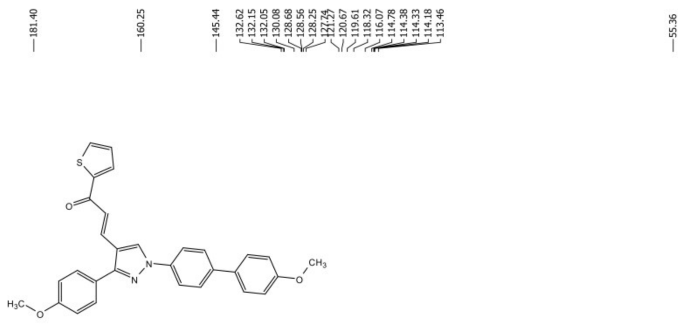

(E)-3-(1-(4'-methoxy-[1,1'-biphenyl]-4-yl)-3-(4-methoxyphenyl)-1H-pyrazol-4-yl)-1-(thiophen-2-yl)prop-2-en-1-one

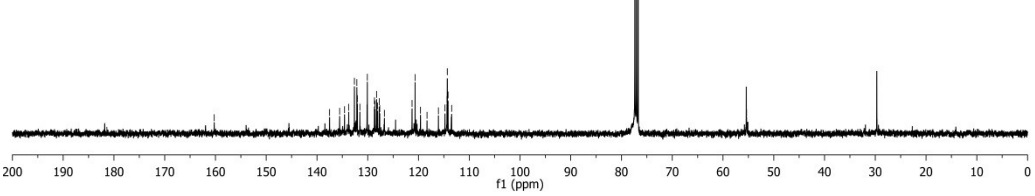

^{13}C-NMR spectra at 100 MHz in $CDCl_3$ of compound 8h

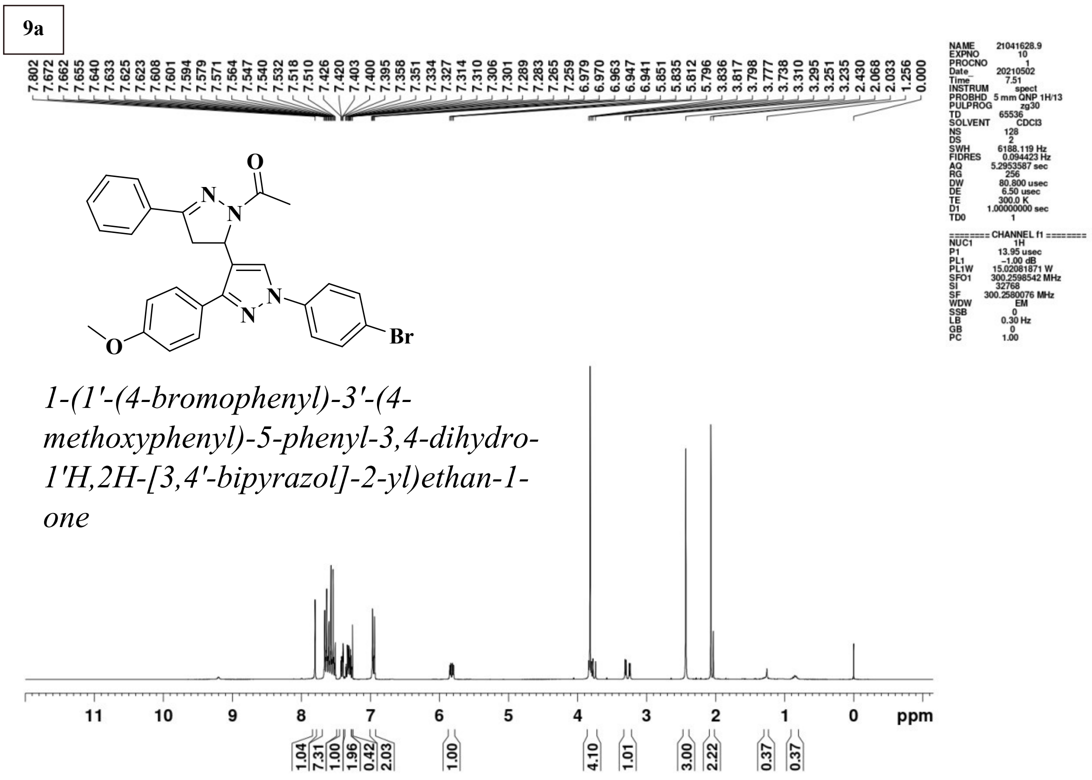

^{1}H-NMR spectra at 300 MHz in $CDCl_3$ of compound 9a

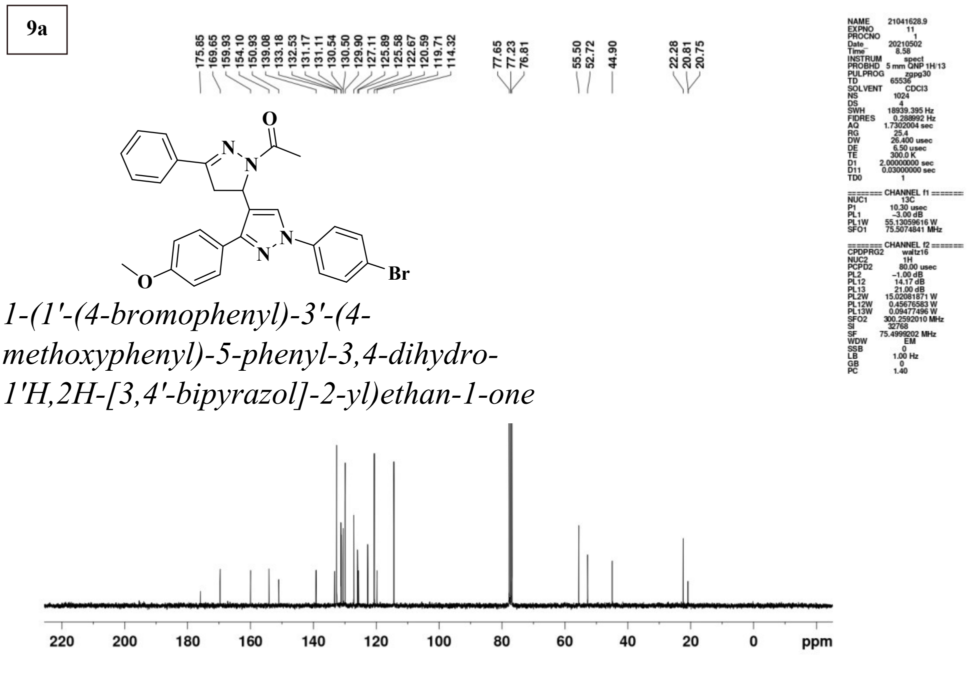

^{13}C-NMR spectra at 75 MHz in $CDCl_3$ of compound 9a

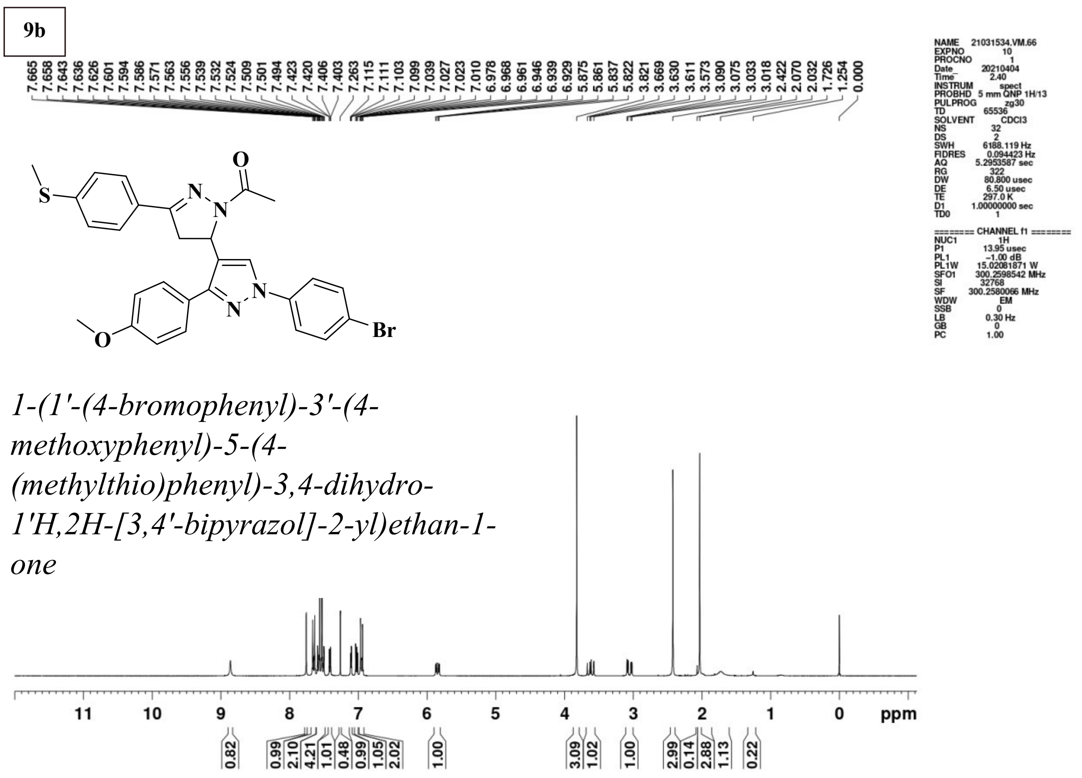

^{1}H-NMR spectra at 300 MHz in $CDCl_3$ of compound 9b

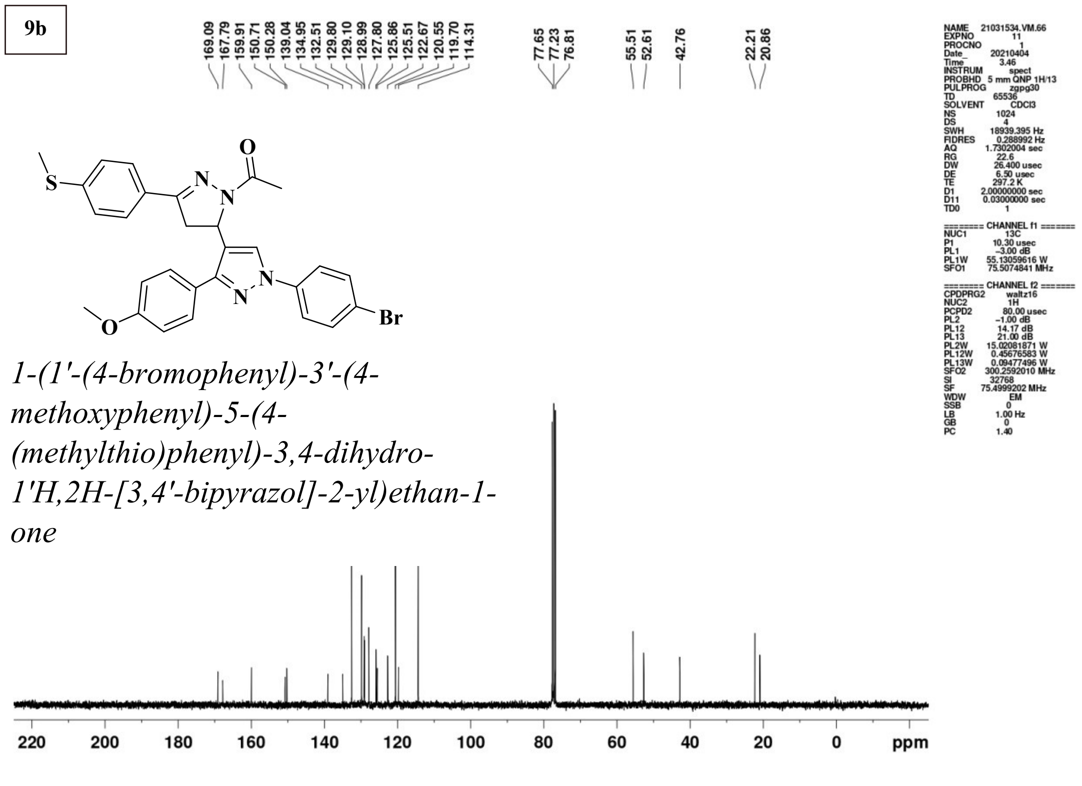

^{13}C-NMR spectra at 75 MHz in $CDCl_3$ of compound 9b

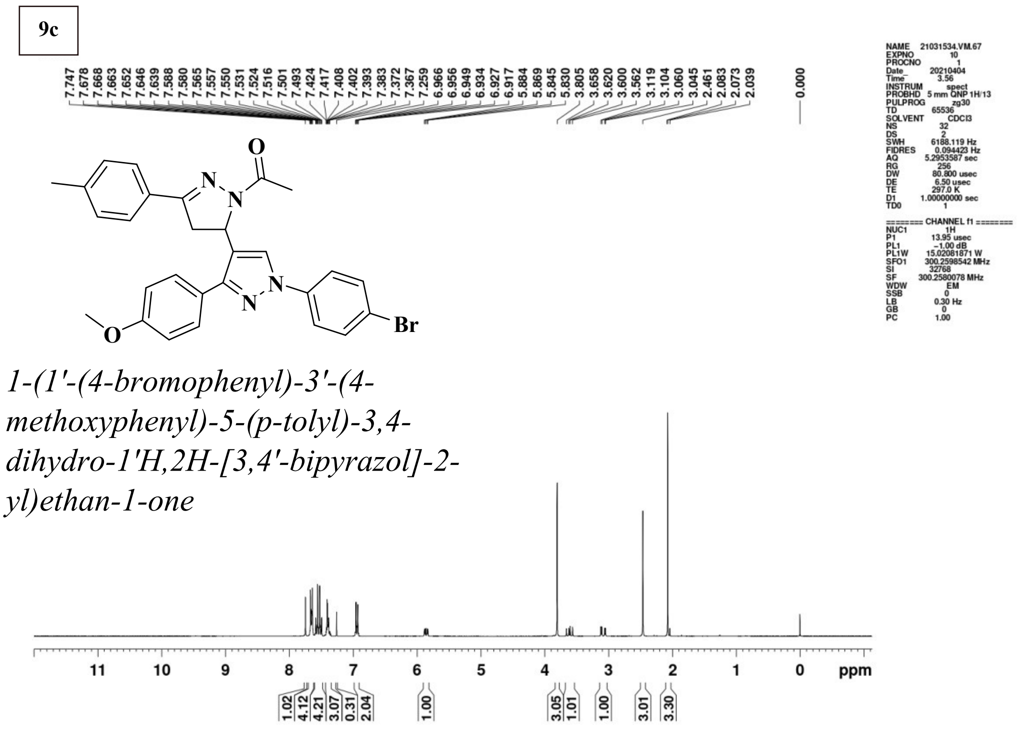

^{1}H-NMR spectra at 300 MHz in $CDCl_3$ of compound 9c

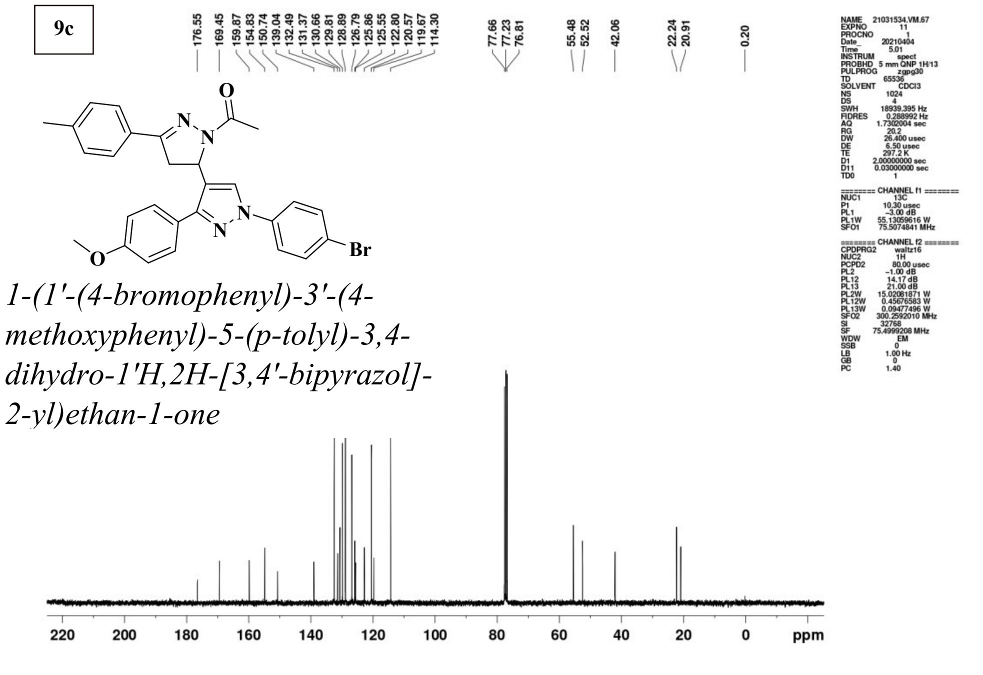

^{13}C-NMR spectra at 75 MHz in $CDCl_3$ of compound 9c

Figure S1. The ^{1}H and ^{13}C NMR spectra of **4**, **5a**, **5b**, **6a-6d**, **7a-7d**, **8a-8h** and **9a-9c** synthesised compounds.

Agilent Technologies

Sample ID:	4 1	Method Name:	jamia
Sample Scans:	64	User:	abid
Background Scans:	16	Date/Time:	30-08-2022 16:55:59
Resolution:	8 cm-1	Range:	4000.00 - 650.00
System Status:	Good	Apodization:	Happ-Genzel
File Location:	C:\Program Files\Agilent\MicroLab PC\Results\Masood\FSC1_2022-08-30T16-56-45.a2r		

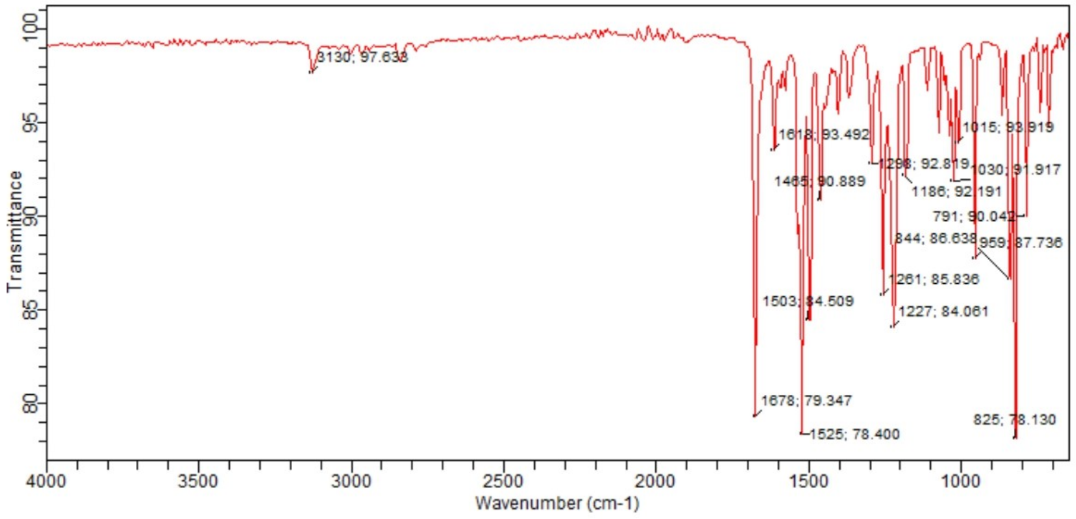

Agilent Technologies

Sample ID:	5a	Method Name:	jamia
Sample Scans:	64	User:	abid
Background Scans:	16	Date/Time:	30-08-2022 16:52:36
Resolution:	8 cm-1	Range:	4000.00 - 650.00
System Status:	Good	Apodization:	Happ-Genzel
File Location:	C:\Program Files\Agilent\MicroLab PC\Results\Masood\SC1_2022-08-30T16-53-31.a2r		

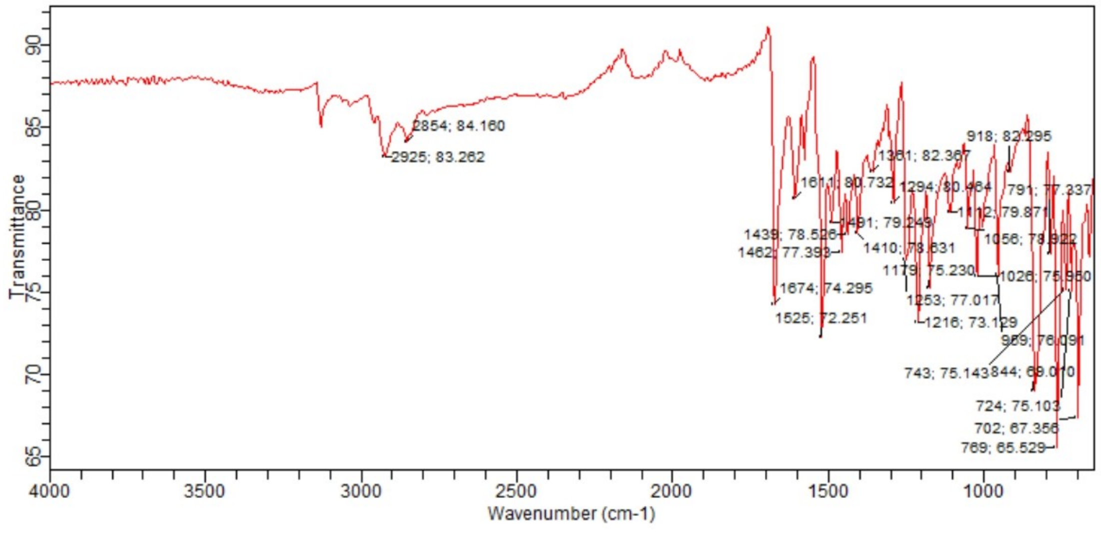

Agilent Technologies

Sample ID: 5b

Sample Scans: 64

Background Scans: 16

Resolution: 8 cm-1

System Status: Good

File Location: C:\Program Files\Agilent\MicroLab PC\Results\Masood\SC2_2022-08-30T16-55-10.a2r

Method Name: jamia

User: abid

Date/Time: 30-08-2022 16:54:29

Range: 4000.00 - 650.00

Apodization: Happ-Genzel

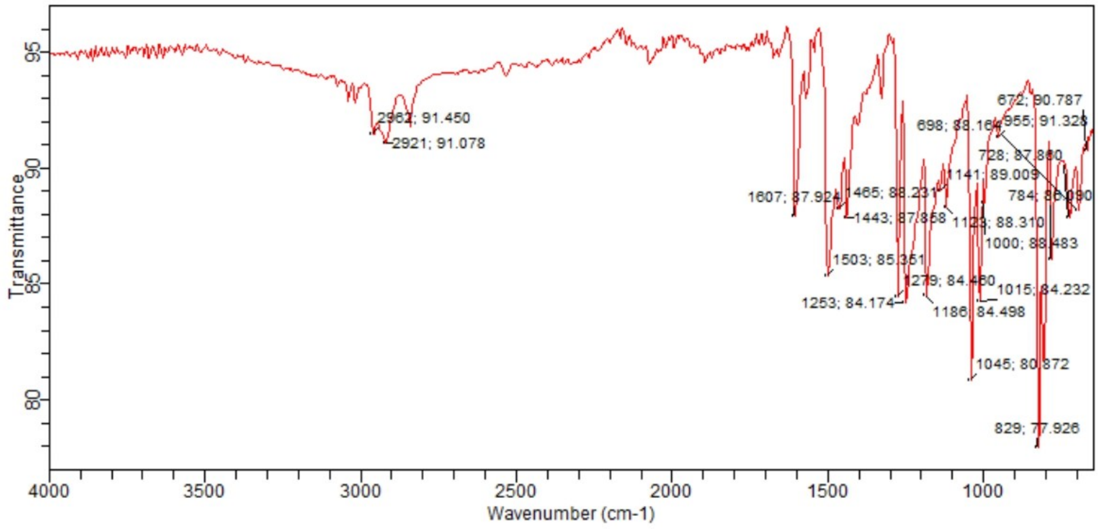

Agilent Technologies

Sample ID: 6a

Sample Scans: 64

Background Scans: 16

Resolution: 8 cm-1

System Status: Good

File Location: C:\Program Files\Agilent\MicroLab PC\Results\Masood\VM39_2022-08-30T17-04-28.a2r

Method Name: jamia

User: abid

Date/Time: 30-08-2022 17:03:50

Range: 4000.00 - 650.00

Apodization: Happ-Genzel

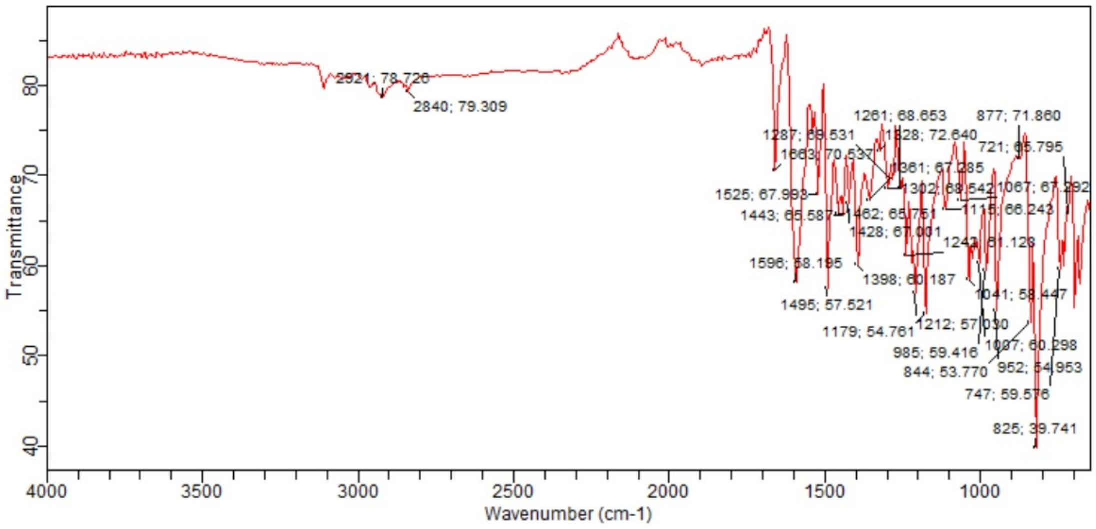

Agilent Technologies

Sample ID:	6b	Method Name:	jamia
Sample Scans:	64	User:	abid
Background Scans:	16	Date/Time:	30-08-2022 16:57:40
Resolution:	8 cm-1	Range:	4000.00 - 650.00
System Status:	Good	Apodization:	Happ-Genzel
File Location:	C:\Program Files\Agilent\MicroLab PC\Results\Masood\VM45_2022-08-30T16-58-17.a2r		

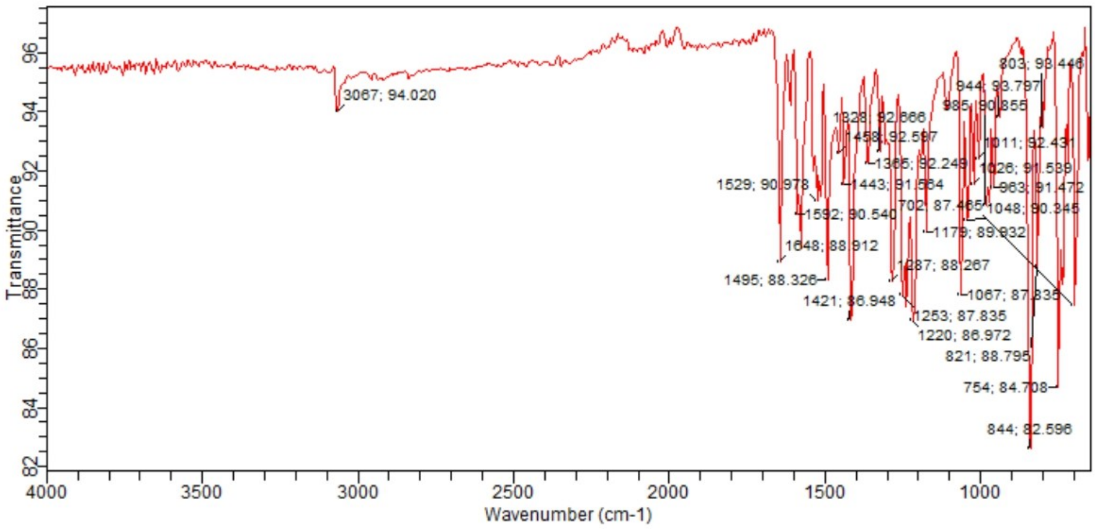

Agilent Technologies

Sample ID:	6c	Method Name:	jamia
Sample Scans:	64	User:	abid
Background Scans:	16	Date/Time:	30-08-2022 17:00:44
Resolution:	8 cm-1	Range:	4000.00 - 650.00
System Status:	Good	Apodization:	Happ-Genzel
File Location:	C:\Program Files\Agilent\MicroLab PC\Results\Masood\VM46_2022-08-30T17-01-22.a2r		

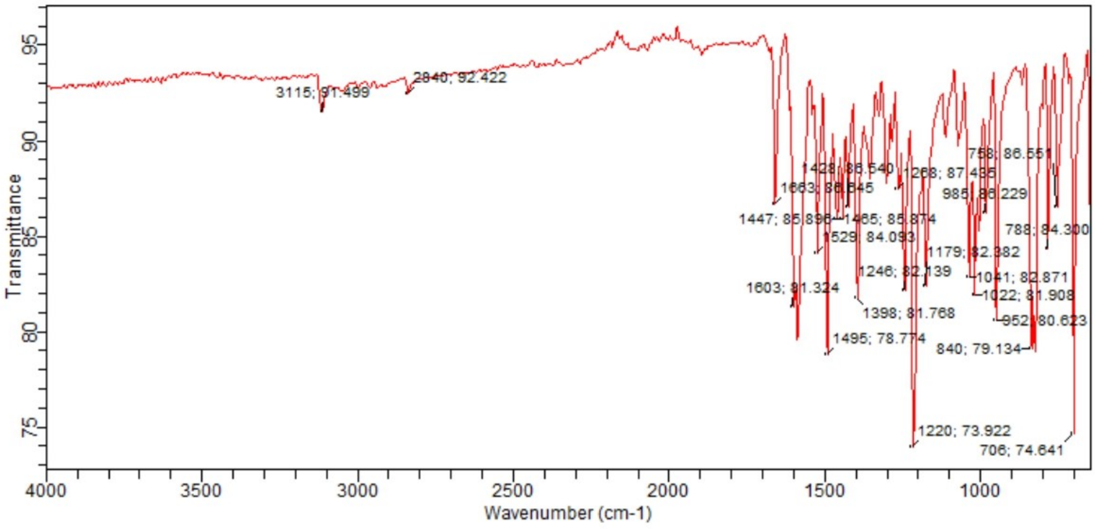

Agilent Technologies

Sample ID:	6d	Method Name:	jamia
Sample Scans:	64	User:	abid
Background Scans:	16	Date/Time:	30-08-2022 17:06:47
Resolution:	8 cm-1	Range:	4000.00 - 650.00
System Status:	Good	Apodization:	Happ-Genzel
File Location:	C:\Program Files\Agilent\MicroLab PC\Results\Masood\VM50_2022-08-30T17-07-40.a2r		

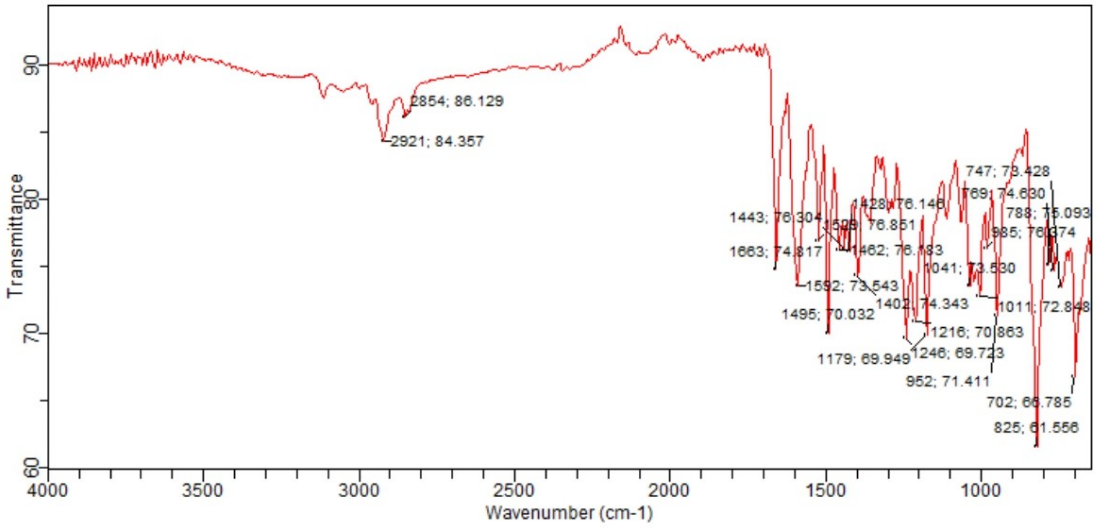

Agilent Technologies

Sample ID:	7a	Method Name:	jamia
Sample Scans:	64	User:	abid
Background Scans:	16	Date/Time:	30-08-2022 17:08:25
Resolution:	8 cm-1	Range:	4000.00 - 650.00
System Status:	Good	Apodization:	Happ-Genzel
File Location:	C:\Program Files\Agilent\MicroLab PC\Results\Masood\VM55_2022-08-30T17-08-59.a2r		

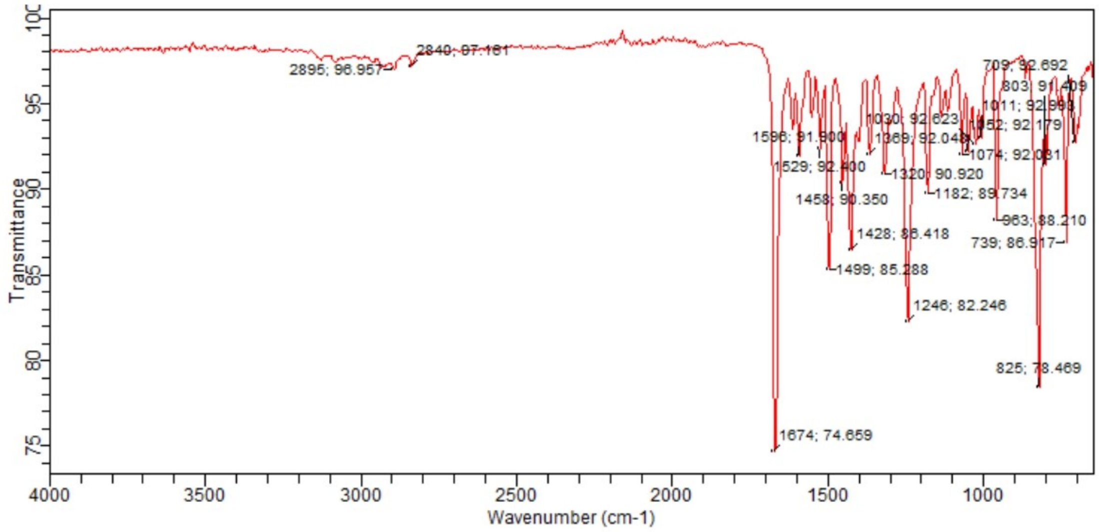

Agilent Technologies

Sample ID:	7b	Method Name:	jamia
Sample Scans:	64	User:	abid
Background Scans:	16	Date/Time:	30-08-2022 17:09:55
Resolution:	8 cm-1	Range:	4000.00 - 650.00
System Status:	Good	Apodization:	Happ-Genzel
File Location:	C:\Program Files\Agilent\MicroLab PC\Results\Masood\VM56_2022-08-30T17-10-33.a2r		

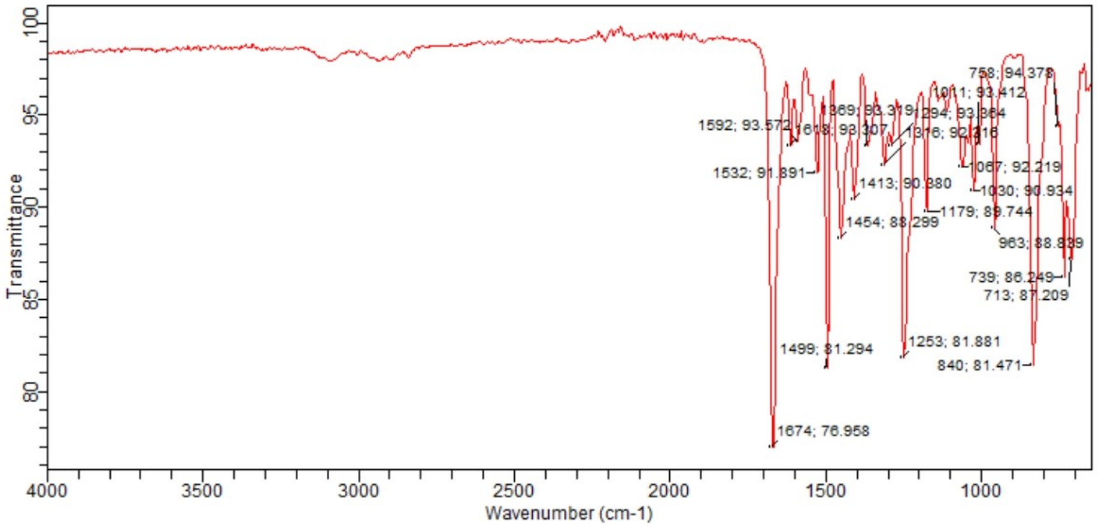

Agilent Technologies

Sample ID:	7c	Method Name:	jamia
Sample Scans:	64	User:	abid
Background Scans:	16	Date/Time:	30-08-2022 17:02:22
Resolution:	8 cm-1	Range:	4000.00 - 650.00
System Status:	Good	Apodization:	Happ-Genzel
File Location:	C:\Program Files\Agilent\MicroLab PC\Results\Masood\VM52_2022-08-30T17-02-56.a2r		

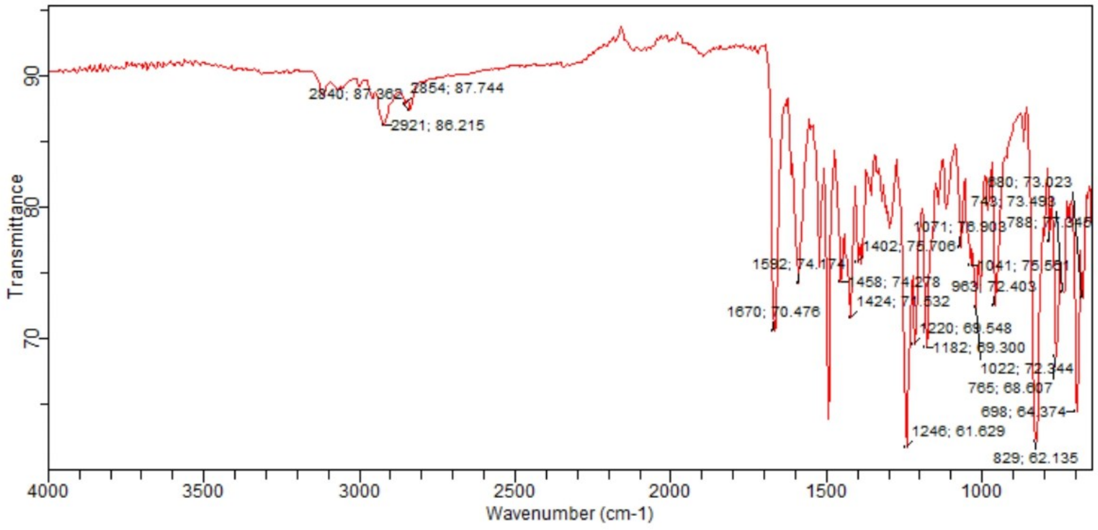

Agilent Technologies

Sample ID:	7d	Method Name:	jamia
Sample Scans:	64	User:	abid
Background Scans:	16	Date/Time:	30-08-2022 17:12:41
Resolution:	8 cm-1	Range:	4000.00 - 650.00
System Status:	Good	Apodization:	Happ-Genzel
File Location:	C:\Program Files\Agilent\MicroLab PC\Results\Masood\12_2022-08-30T17-13-23.a2r		

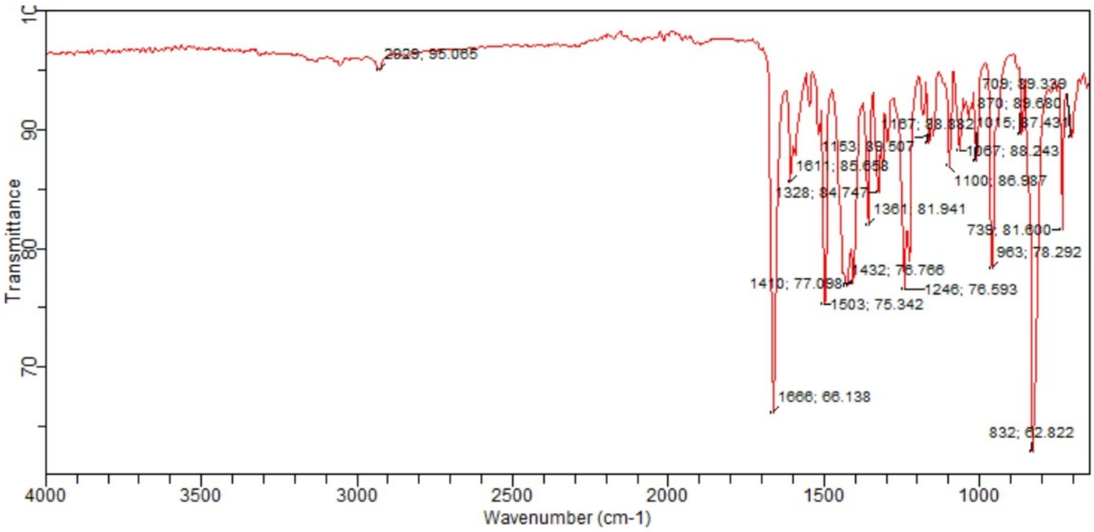

Agilent Technologies

Sample ID:	8a	Method Name:	jamia
Sample Scans:	64	User:	abid
Background Scans:	16	Date/Time:	30-08-2022 16:36:11
Resolution:	8 cm-1	Range:	4000.00 - 650.00
System Status:	Good	Apodization:	Happ-Genzel
File Location:	C:\Program Files\Agilent\MicroLab PC\Results\Masood\SC16_2022-08-30T16-37-27.a2r		

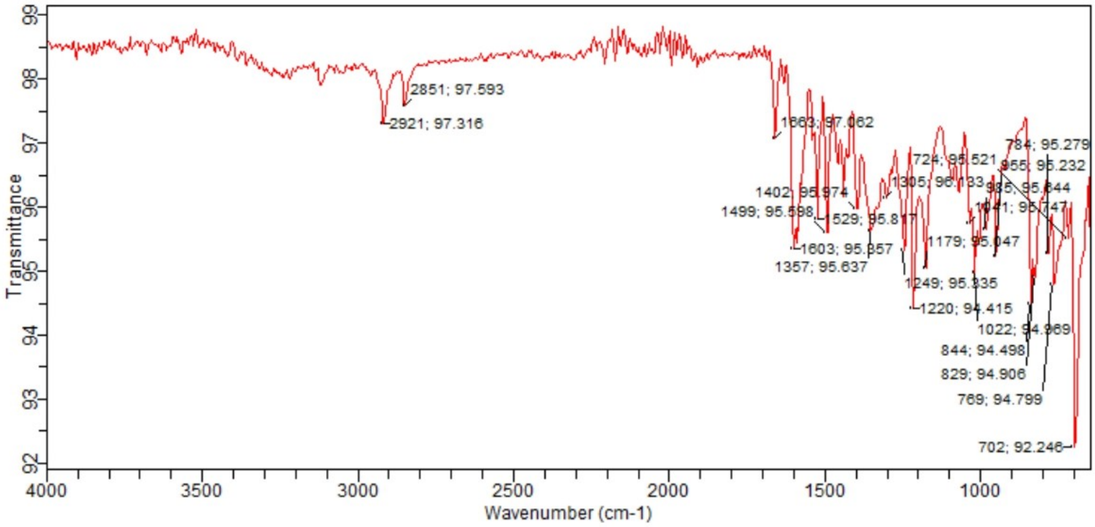

Agilent Technologies

Sample ID:	8b	Method Name:	jamia
Sample Scans:	64	User:	abid
Background Scans:	16	Date/Time:	30-08-2022 16:49:29
Resolution:	8 cm-1	Range:	4000.00 - 650.00
System Status:	Good	Apodization:	Happ-Genzel

File Location: C:\Program Files\Agilent\MicroLab PC\Results\Masood\SC17_2022-08-30T16-50-07.a2r

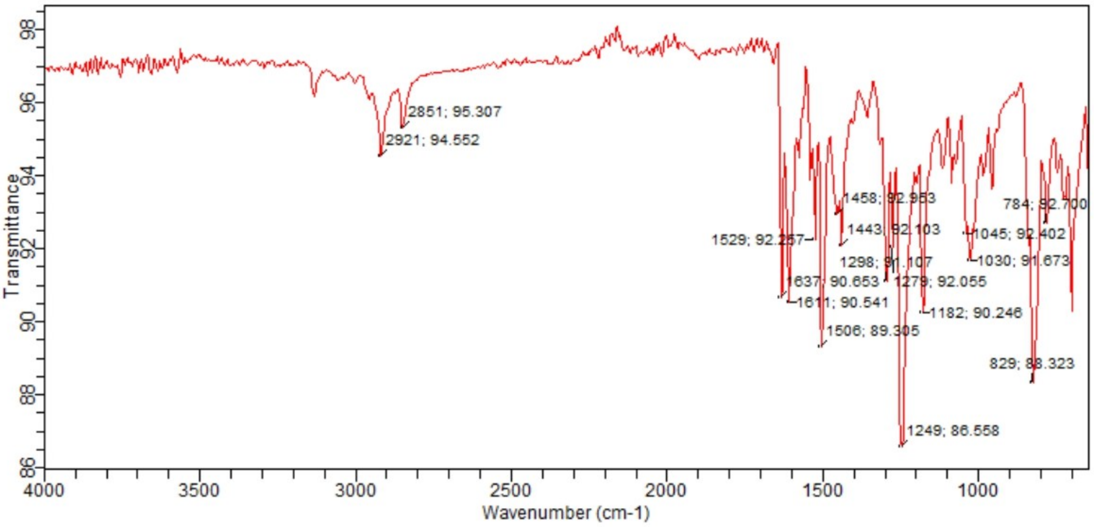

Agilent Technologies

Sample ID:	8c	Method Name:	jamia
Sample Scans:	64	User:	abid
Background Scans:	16	Date/Time:	30-08-2022 16:39:46
Resolution:	8 cm-1	Range:	4000.00 - 650.00
System Status:	Good	Apodization:	Happ-Genzel

File Location: C:\Program Files\Agilent\MicroLab PC\Results\Masood\SC19_2022-08-30T16-40-44.a2r

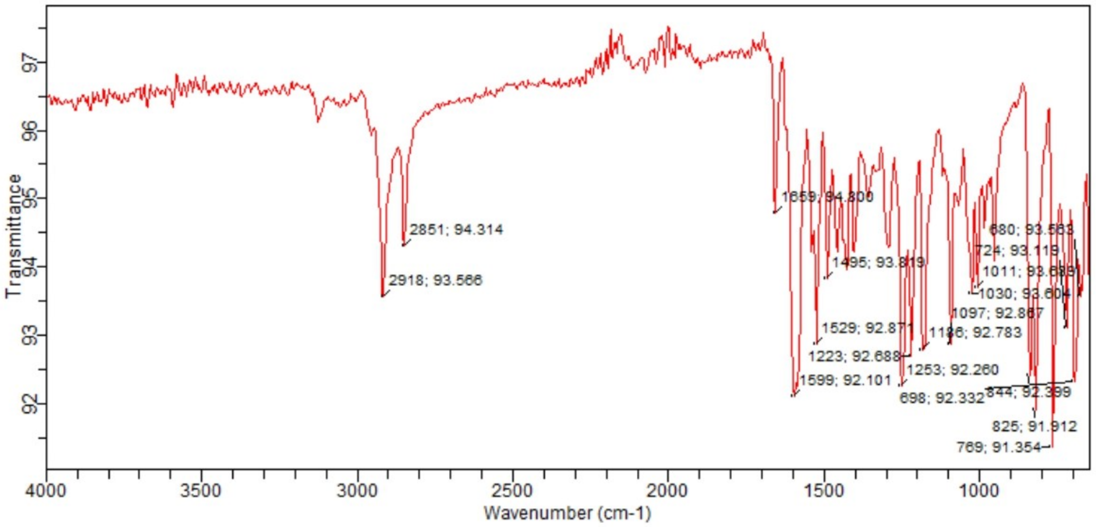

Agilent Technologies

Sample ID:	8d	Method Name:	jamia
Sample Scans:	64	User:	abid
Background Scans:	16	Date/Time:	30-08-2022 16:45:56
Resolution:	8 cm-1	Range:	4000.00 - 650.00
System Status:	Good	Apodization:	Happ-Genzel
File Location:	C:\Program Files\Agilent\MicroLab PC\Results\Masood\SC20_2022-08-30T16-46-35.a2r		

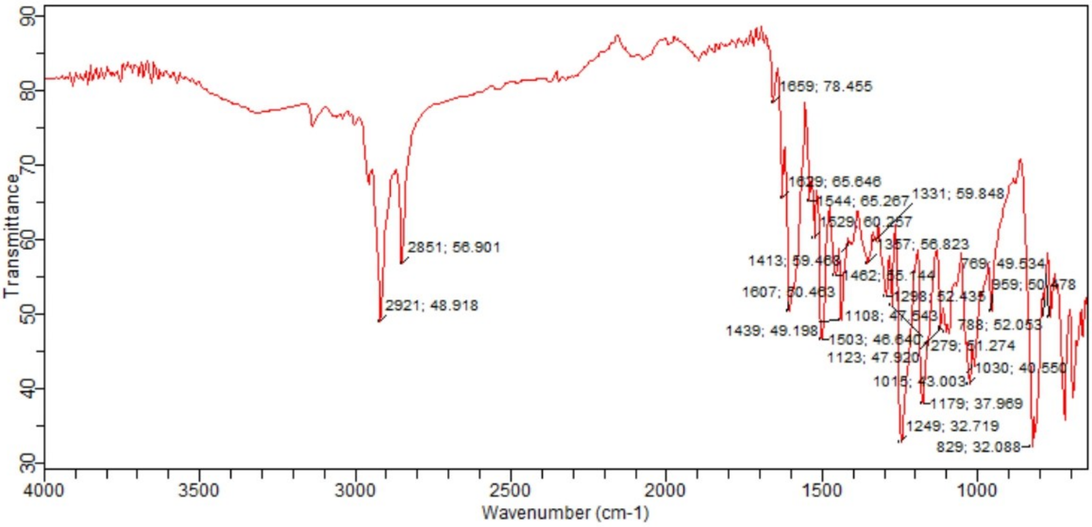

Agilent Technologies

Sample ID:	8e	Method Name:	jamia
Sample Scans:	64	User:	abid
Background Scans:	16	Date/Time:	30-08-2022 16:44:19
Resolution:	8 cm-1	Range:	4000.00 - 650.00
System Status:	Good	Apodization:	Happ-Genzel
File Location:	C:\Program Files\Agilent\MicroLab PC\Results\Masood\SC22_2022-08-30T16-44-59.a2r		

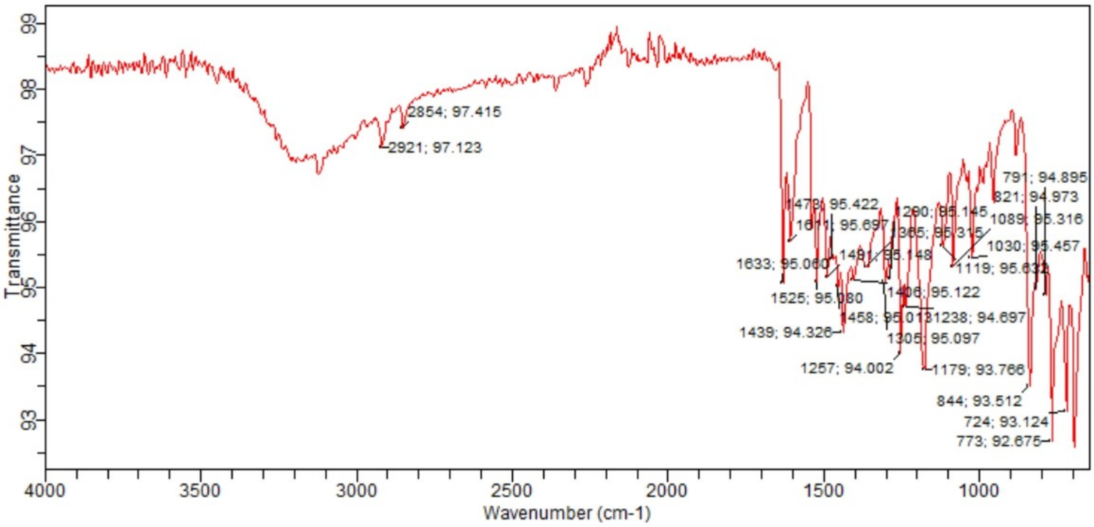

Agilent Technologies

Sample ID:	**8f**	Method Name:	jamia
Sample Scans:	64	User:	abid
Background Scans:	16	Date/Time:	30-08-2022 16:47:49
Resolution:	8 cm-1	Range:	4000.00 - 650.00
System Status:	Good	Apodization:	Happ-Genzel
File Location:	C:\Program Files\Agilent\MicroLab PC\Results\Masood\SC23_2022-08-30T16-48-32.a2r		

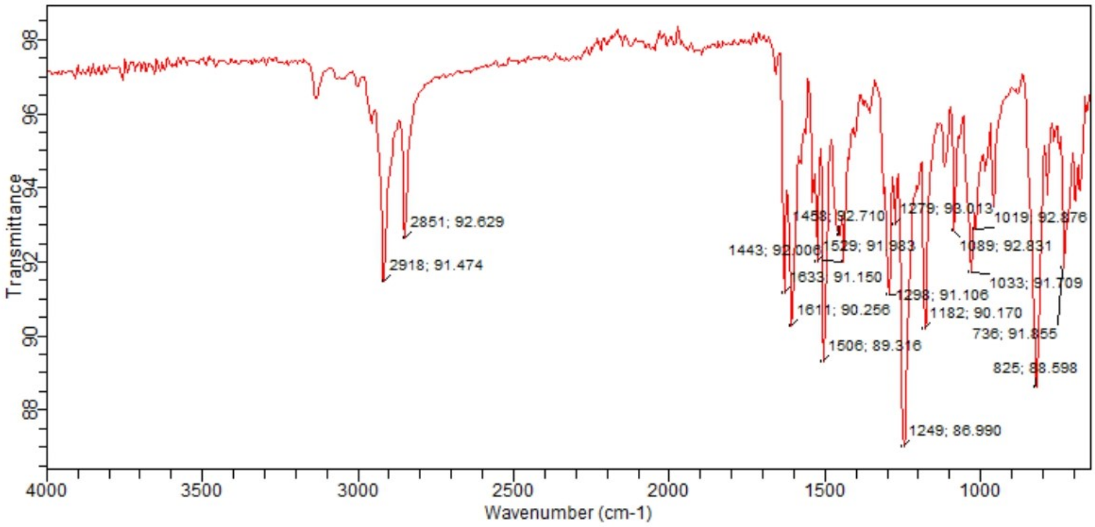

Agilent Technologies

Sample ID:	**8g**	Method Name:	jamia
Sample Scans:	64	User:	abid
Background Scans:	16	Date/Time:	30-08-2022 16:42:12
Resolution:	8 cm-1	Range:	4000.00 - 650.00
System Status:	Good	Apodization:	Happ-Genzel
File Location:	C:\Program Files\Agilent\MicroLab PC\Results\Masood\SC24_2022-08-30T16-42-49.a2r		

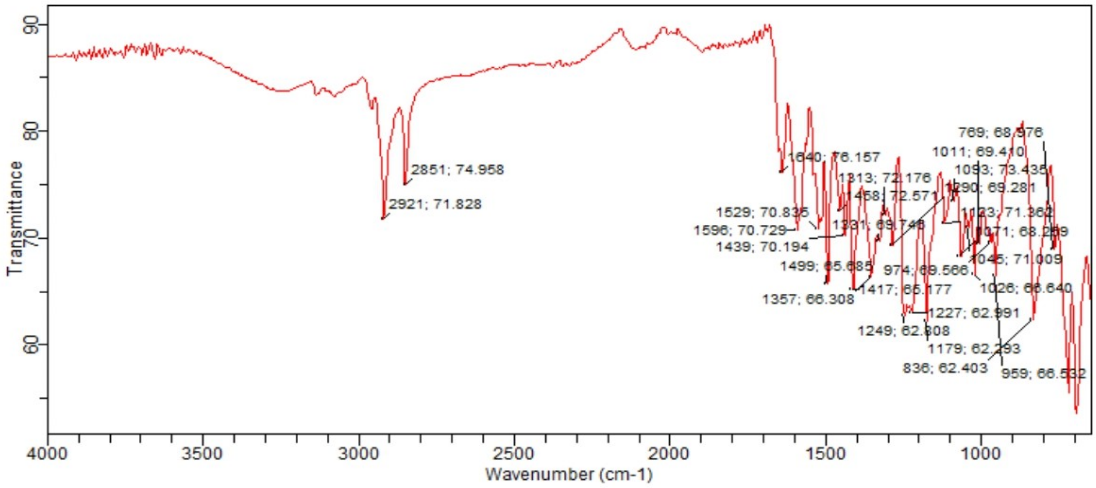

Agilent Technologies

Sample ID:	**8h**	Method Name:	jamia
Sample Scans:	64	User:	abid
Background Scans:	16	Date/Time:	30-08-2022 16:51:03
Resolution:	8 cm-1	Range:	4000.00 - 650.00
System Status:	Good	Apodization:	Happ-Genzel
File Location:	C:\Program Files\Agilent\MicroLab PC\Results\Masood\SC25_2022-08-30T16-51-44.a2r		

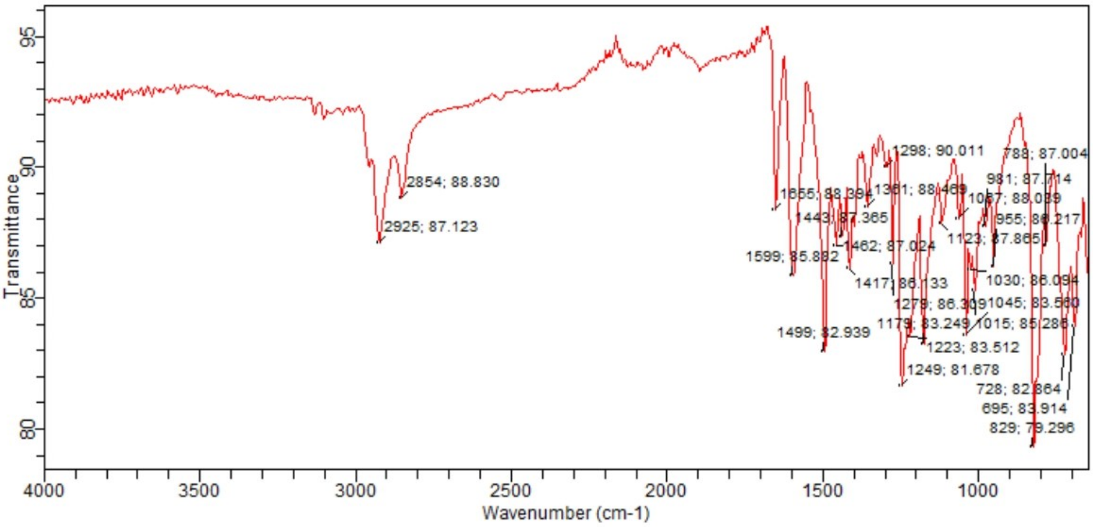

Agilent Technologies

Sample ID:	**9a**	Method Name:	jamia
Sample Scans:	64	User:	abid
Background Scans:	16	Date/Time:	30-08-2022 17:11:20
Resolution:	8 cm-1	Range:	4000.00 - 650.00
System Status:	Good	Apodization:	Happ-Genzel
File Location:	C:\Program Files\Agilent\MicroLab PC\Results\Masood\VM64_2022-08-30T17-11-57.a2r		

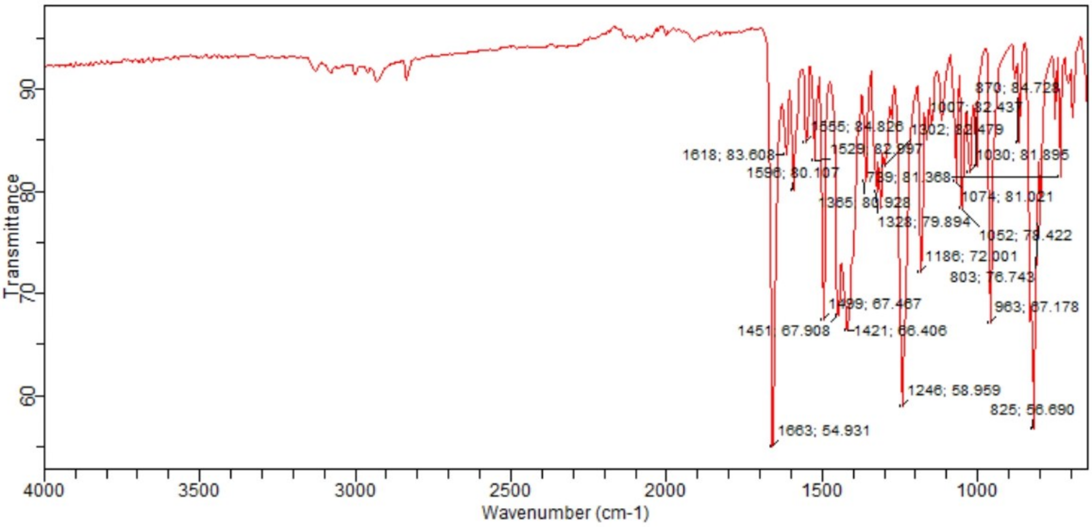

Agilent Technologies

Sample ID:	9b	Method Name:	jamia
Sample Scans:	64	User:	abid
Background Scans:	16	Date/Time:	30-08-2022 17:05:21
Resolution:	8 cm-1	Range:	4000.00 - 650.00
System Status:	Good	Apodization:	Happ-Genzel
File Location:	C:\Program Files\Agilent\MicroLab PC\Results\Masood\VM66_2022-08-30T17-05-58.a2r		

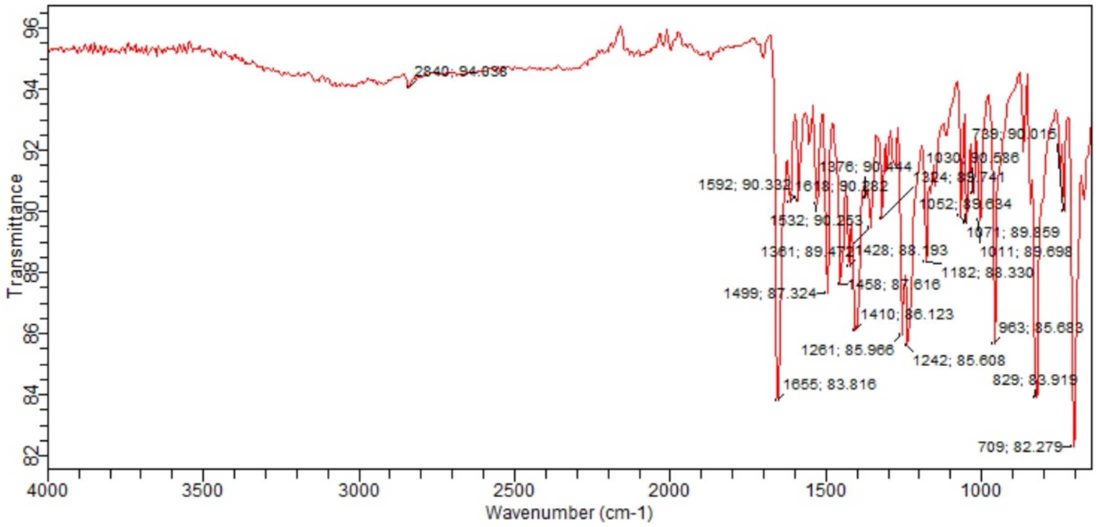

Agilent Technologies

Sample ID:	9c	Method Name:	jamia
Sample Scans:	64	User:	abid
Background Scans:	16	Date/Time:	30-08-2022 16:59:18
Resolution:	8 cm-1	Range:	4000.00 - 650.00
System Status:	Good	Apodization:	Happ-Genzel
File Location:	C:\Program Files\Agilent\MicroLab PC\Results\Masood\VM67_2022-08-30T16-59-55.a2r		

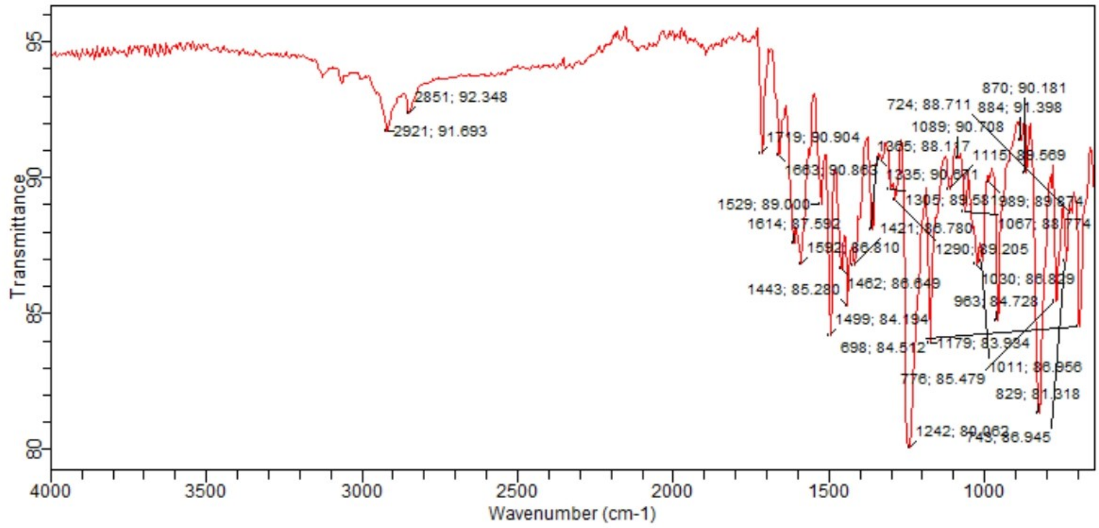

Agilent Technologies

Sample ID:	9d	Method Name:	jamia
Sample Scans:	64	User:	abid
Background Scans:	16	Date/Time:	30-08-2022 17:14:14
Resolution:	8 cm-1	Range:	4000.00 - 650.00
System Status:	Good	Apodization:	Happ-Genzel
File Location:	C:\Program Files\Agilent\MicroLab PC\Results\Masood\3_2022-08-30T17-14-52.a2r		

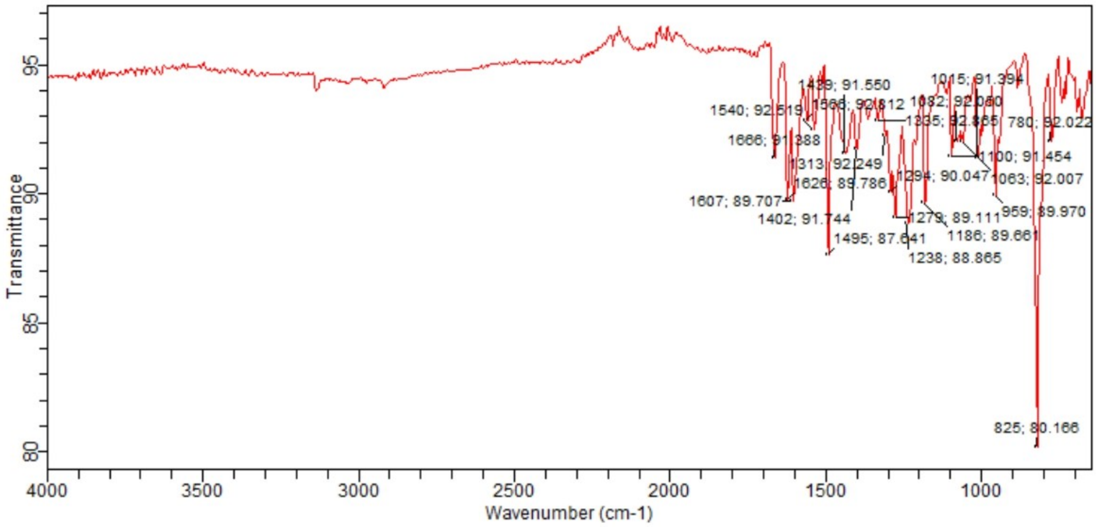

Figure S2. The FT-IR spectra of **4**, **5a**, **5b**, **6a-6d**, **7a-7d**, **8a-8h** and **9a-9d** synthesised compounds.

356

Sample Name : P-1
Test Name : NEW- JBL-3-MIN-elsd
Date & Time : 02-Sep-2022,20:45:03

Instrument ID # LCM-0001
INTERNAL

4

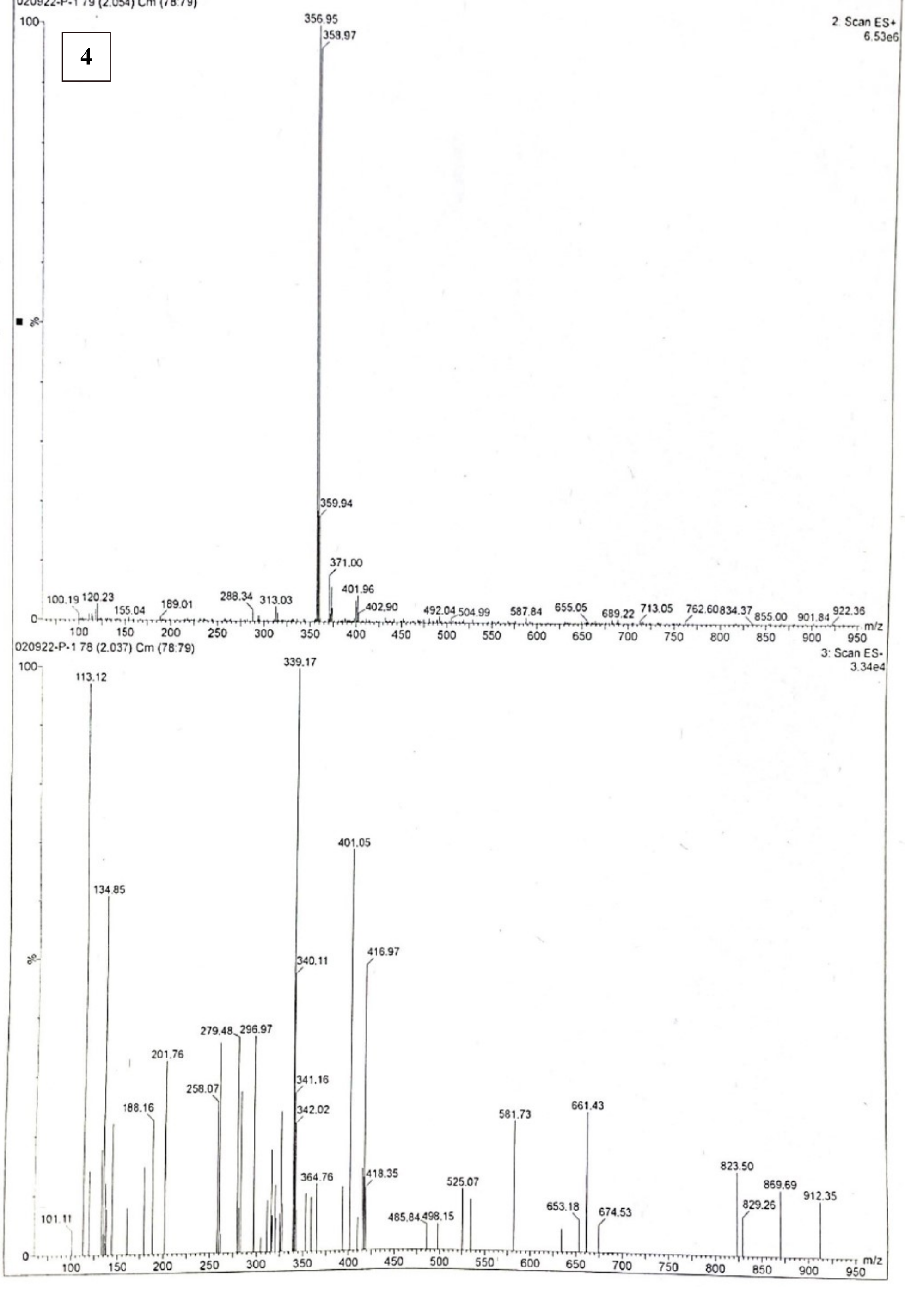

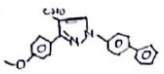

Sample Name : M-1
Test Name : NEW-4-MIN-AA-1
Date & Time : 16-Sep-2021, 15:01:16

Instrument ID # QDA 01
Internal

5a

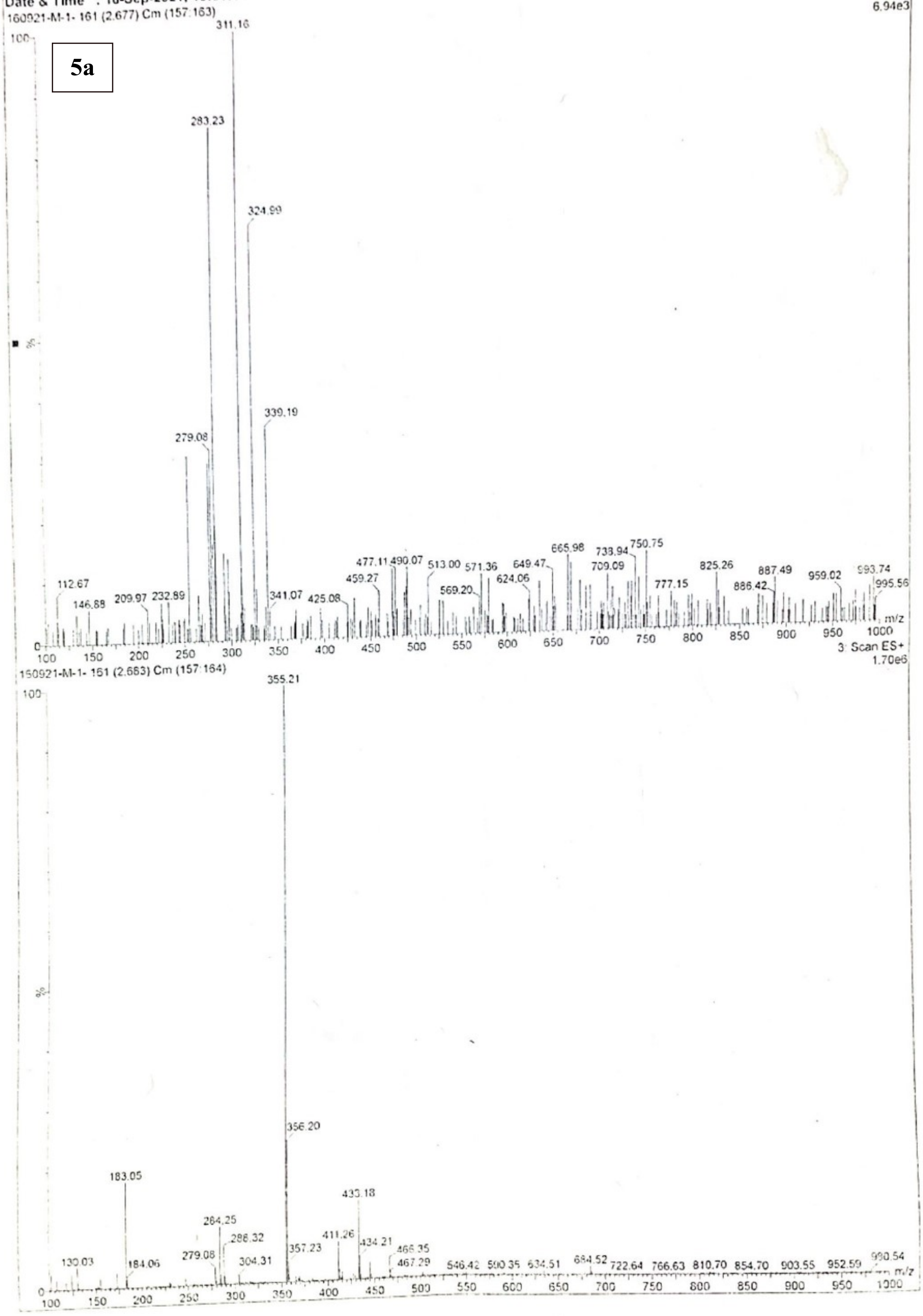

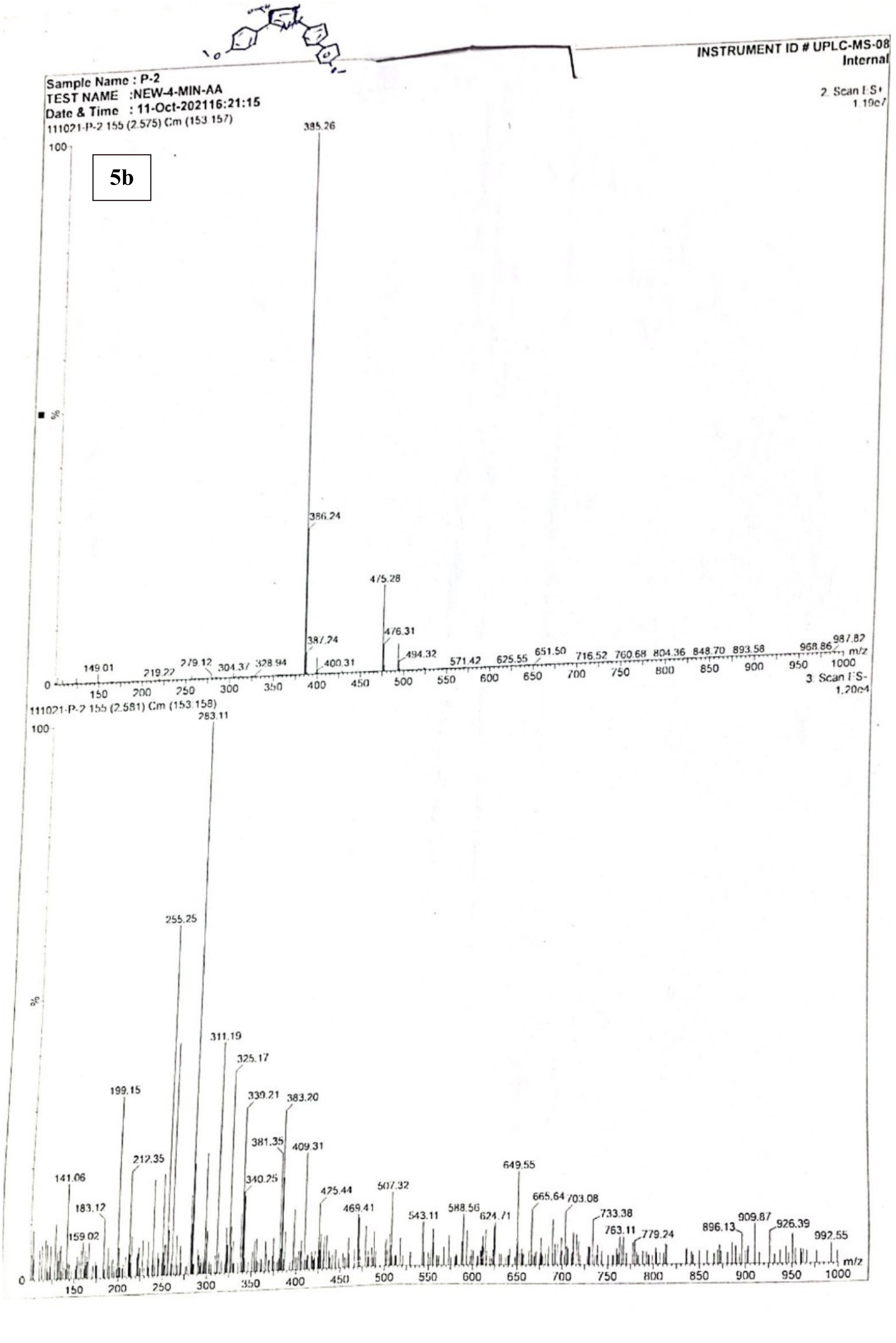

INSTRUMENT ID # UPLC-MS-08
Internal
Sample Name : P-2
TEST NAME :NEW-4-MIN-AA
Date & Time : 11-Oct-202116:21:15
111021-P-2 155 (2.575) Cm (153:157)
2: Scan ES+
1.19e7
5b
385.26
386.24
475.28
476.31
387.24
400.31
494.32
149.01
219.22
279.12
304.37
328.94
571.42
625.55
651.50
716.52
760.68
804.36
848.70
893.58
968.86
987.82
m/z
3: Scan ES-
1.20e4
111021-P-2 155 (2.581) Cm (153:158)
283.11
255.25
311.19
325.17
199.15
339.21
383.20
381.35
409.31
212.35
649.55
141.06
340.25
475.44
507.32
665.64
703.08
183.12
469.41
543.11
588.56
624.71
733.38
763.11
779.24
896.13
909.87
926.39
992.55
159.02
%

458

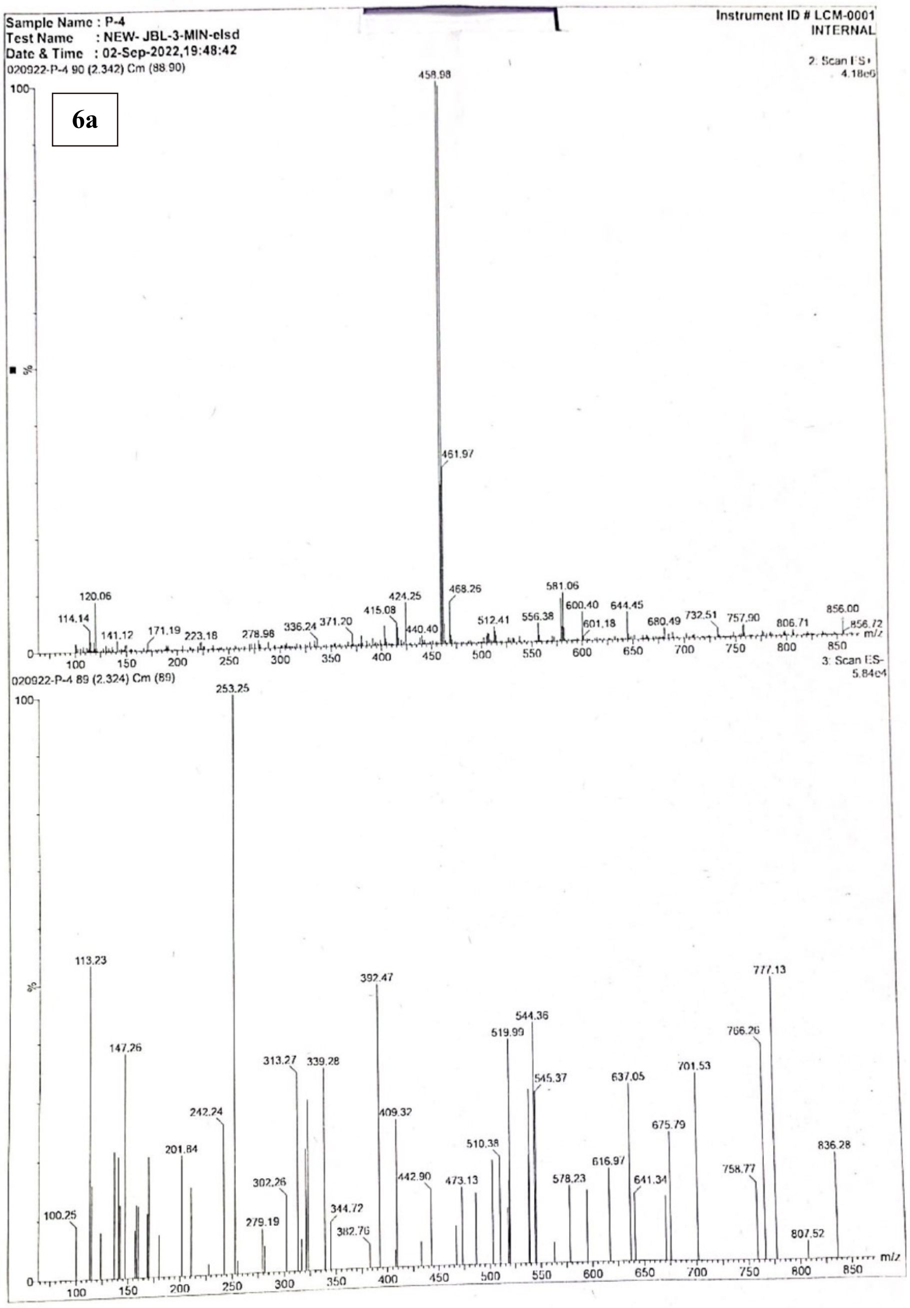

Sample Name : P-4
Test Name : NEW- JBL-3-MIN-elsd
Date & Time : 02-Sep-2022,19:48:42
Instrument ID # LCM-0001
INTERNAL
020922-P-4 90 (2.342) Cm (88:90)
2: Scan ES+
4.18e6
6a
458.98
461.97
468.26
424.25
415.08
440.40
120.06
114.14
141.12
171.19
223.18
278.96
336.24
371.20
512.41
556.38
581.06
600.40
601.18
644.45
680.49
732.51
757.90
806.71
856.00
856.72
m/z
020922-P-4 89 (2.324) Cm (89)
3: Scan ES-
5.84e4
253.25
113.23
147.26
392.47
777.13
544.36
519.99
766.26
313.27
339.28
545.37
637.05
701.53
242.24
409.32
675.79
201.84
510.38
616.97
836.28
302.26
442.90
473.13
578.23
641.34
758.77
100.25
279.19
344.72
382.76
807.52
%

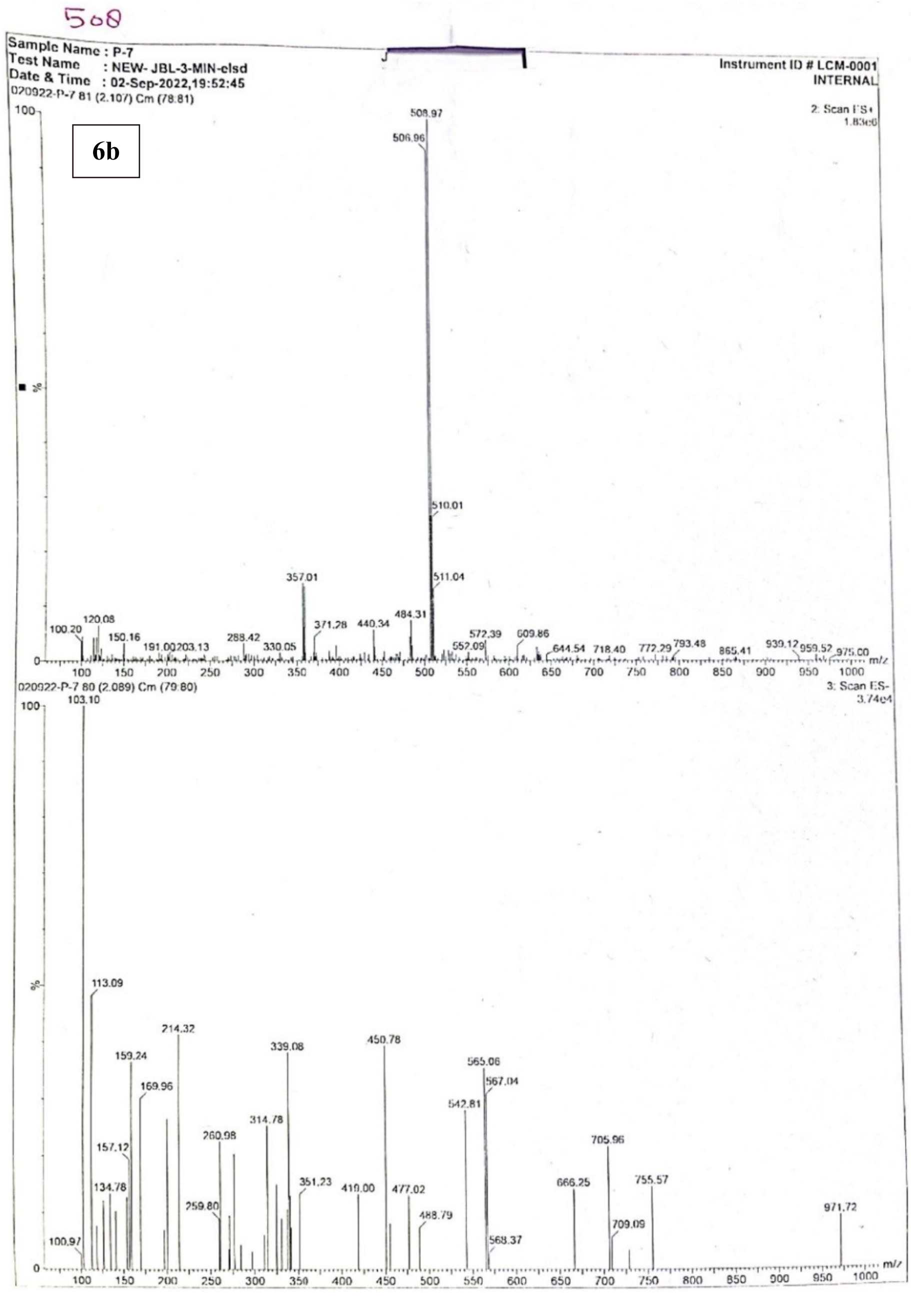
508
Sample Name : P-7
Test Name : NEW- JBL-3-MIN-elsd
Date & Time : 02-Sep-2022,19:52:45
Instrument ID # LCM-0001
INTERNAL
020922-P-7 81 (2.107) Cm (78:81)
2: Scan ES+
1.83e6
6b
508.97
506.96
510.01
511.04
357.01
484.31
440.34
371.28
100.20
120.08
150.16
191.00
203.13
288.42
330.05
572.39
609.86
552.09
644.54
718.40
772.29
793.48
865.41
939.12
959.52
975.00
020922-P-7 80 (2.089) Cm (79:80)
3: Scan ES-
3.74e4
103.10
113.09
214.32
159.24
169.96
339.08
450.78
565.08
567.04
542.81
314.78
260.98
157.12
134.78
259.80
351.23
419.00
477.02
488.79
568.37
666.25
705.96
709.09
755.57
971.72
100.97
%
m/z

6c

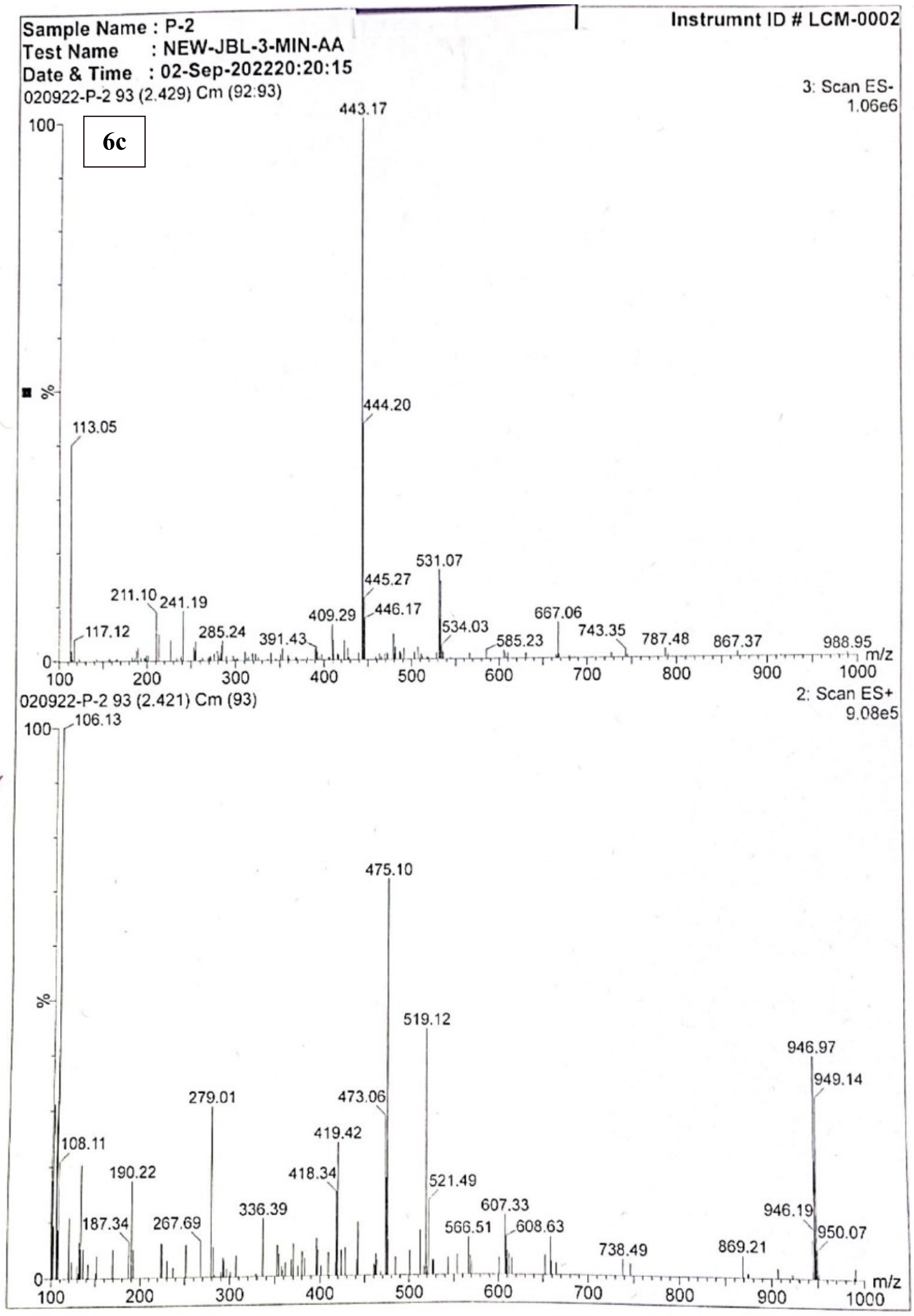

Sample Name : P-2
Test Name : NEW-JBL-3-MIN-AA
Date & Time : 02-Sep-202220:20:15
Instrumnt ID # LCM-0002
020922-P-2 93 (2.429) Cm (92:93)
3: Scan ES-
1.06e6
443.17
444.20
113.05
531.07
445.27
211.10
241.19
409.29
446.17
117.12
285.24
391.43
534.03
667.06
743.35
585.23
787.48
867.37
988.95
m/z
020922-P-2 93 (2.421) Cm (93)
2: Scan ES+
9.08e5
106.13
475.10
519.12
946.97
949.14
279.01
473.06
419.42
108.11
190.22
418.34
521.49
607.33
336.39
946.19
187.34
267.69
566.51
608.63
950.07
738.49
869.21
472

466

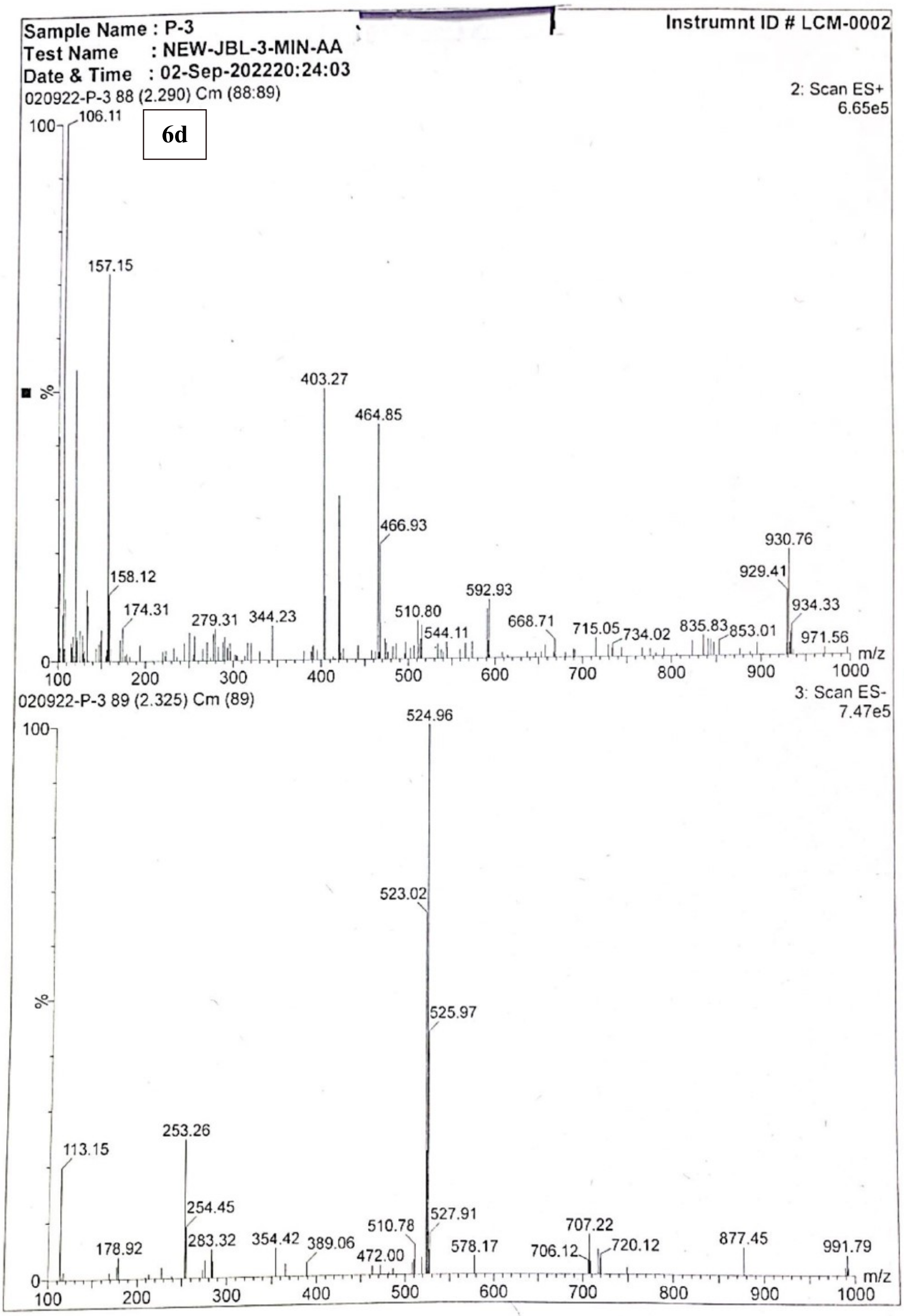

Sample Name : P-3
Test Name : NEW-JBL-3-MIN-AA
Date & Time : 02-Sep-202220:24:03
Instrumnt ID # LCM-0002
6d
020922-P-3 88 (2.290) Cm (88:89)
2: Scan ES+
6.65e5
106.11
157.15
403.27
464.85
466.93
930.76
929.41
934.33
158.12
174.31
279.31
344.23
510.80
544.11
592.93
668.71
715.05
734.02
835.83
853.01
971.56
m/z
020922-P-3 89 (2.325) Cm (89)
3: Scan ES-
7.47e5
524.96
523.02
525.97
113.15
253.26
254.45
283.32
354.42
389.06
472.00
510.78
527.91
578.17
178.92
707.22
706.12
720.12
877.45
991.79
%

502

Sample Name : P-01
Test Name : NEW- JBL-3-MIN-elsd
Date & Time : 02-Sep-2022,20:48:58

Instrument ID # LCM-000
INTERNA

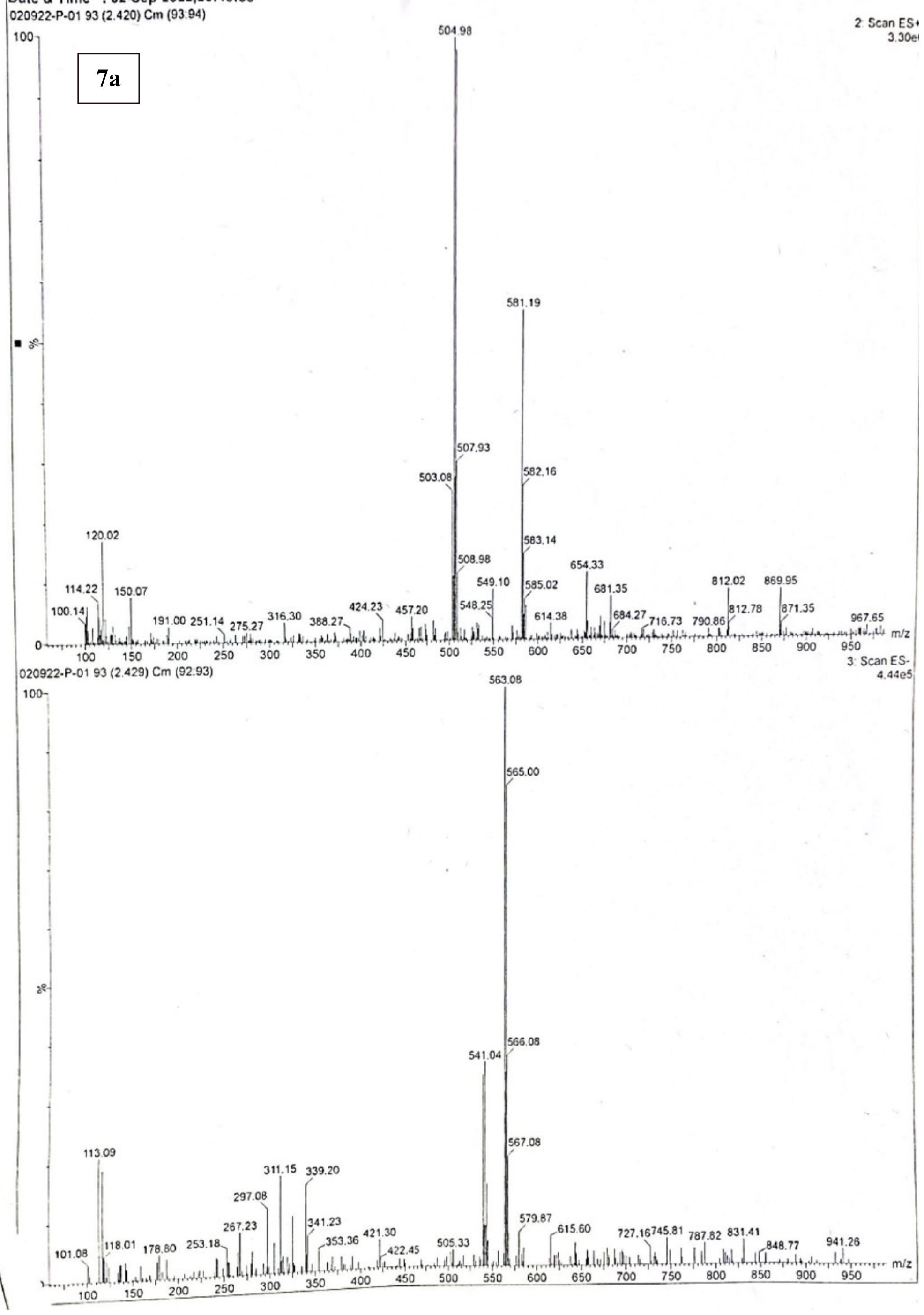

514

7b

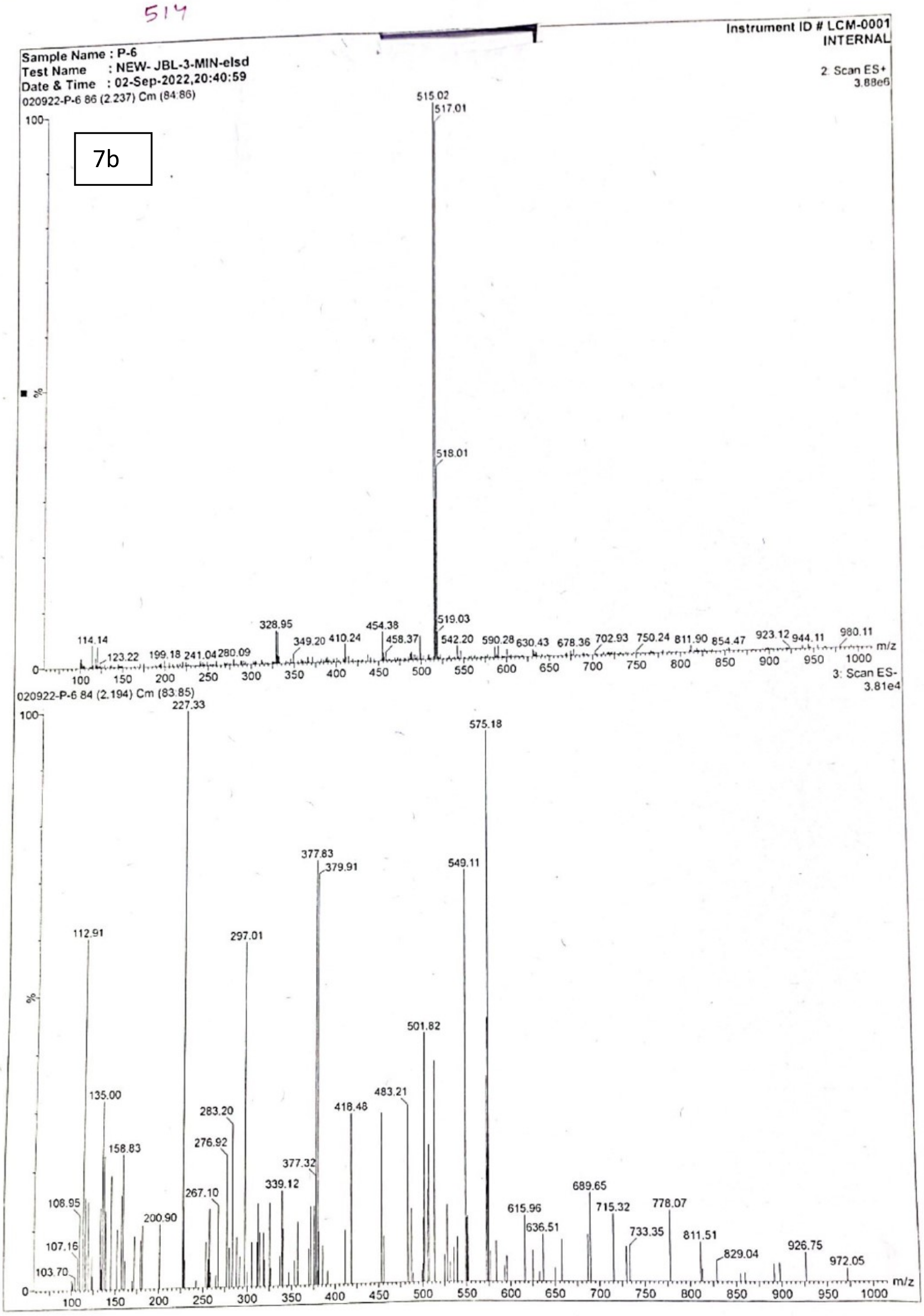

Instrument ID # LCM-0001
INTERNAL
Sample Name : P-6
Test Name : NEW- JBL-3-MIN-elsd
Date & Time : 02-Sep-2022,20:40:59
020922-P-6 86 (2.237) Cm (84:86)
2: Scan ES+
3.88e6
515.02
517.01
518.01
519.03
114.14
123.22
199.18
241.04
280.09
328.95
349.20
410.24
454.38
458.37
542.20
590.28
630.43
678.36
702.93
750.24
811.90
854.47
923.12
944.11
980.11
m/z
020922-P-6 84 (2.194) Cm (83:85)
3: Scan ES-
3.81e4
227.33
575.18
377.83
379.91
549.11
112.91
297.01
501.82
135.00
483.21
418.48
283.20
276.92
158.83
377.32
339.12
267.10
108.95
200.90
107.16
103.70
615.96
636.51
689.65
715.32
778.07
733.35
811.51
829.04
926.75
972.05
m/z
%

548-12

Sample Name :P-3
Test Name :NEW-JBL-3-MIN-AA
Date & Time :14-Sep-202221:15:10

Instrument ID # LCM-0005
INTERNAL

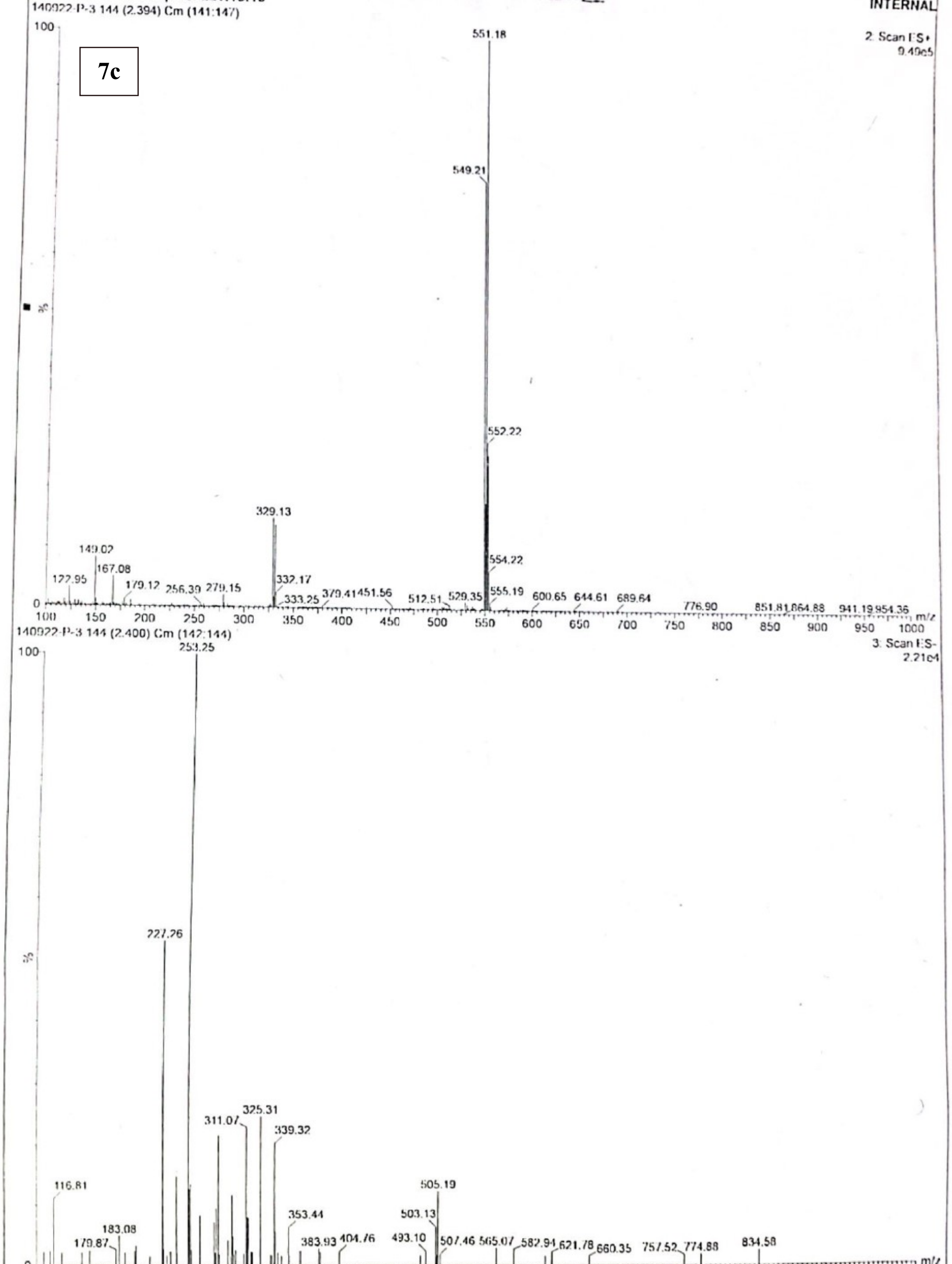

500

Sample Name : P-8 [redacted] Instrumnt ID # LCM-0002
Test Name : NEW-JBL-3-MIN-AA
Date & Time : 02-Sep-202220:27:50

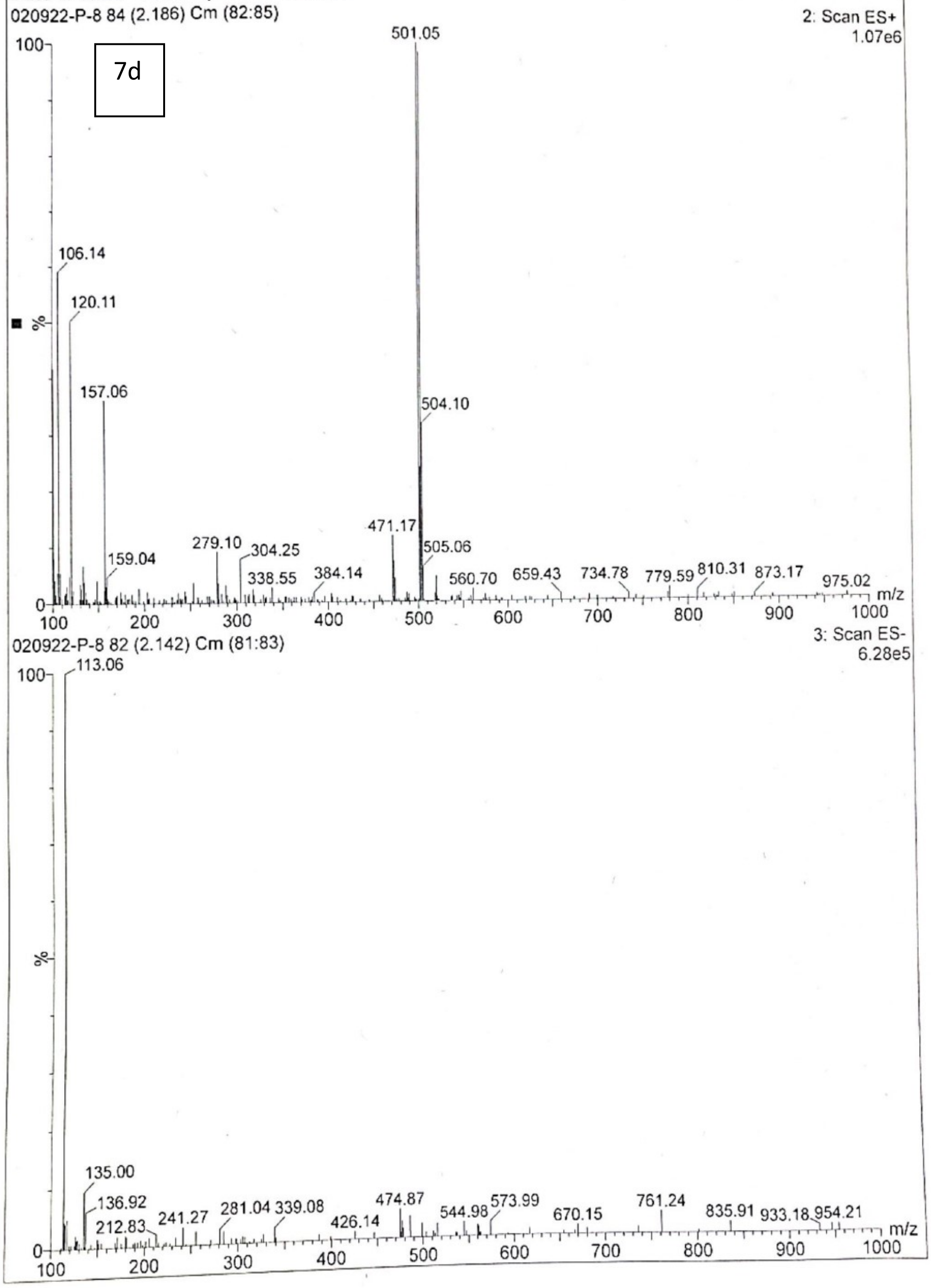

8a

8b

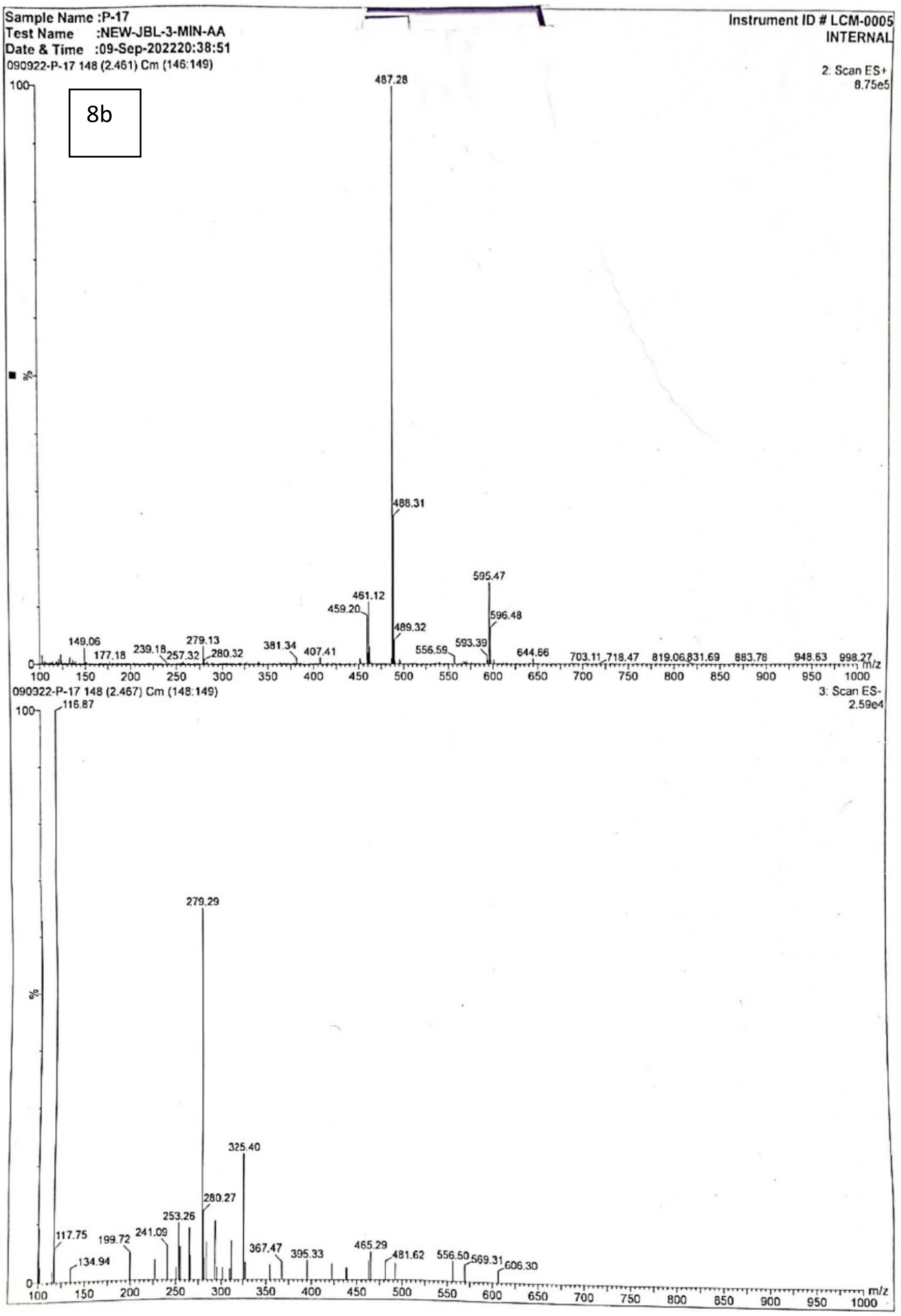
Sample Name :P-17
Test Name :NEW-JBL-3-MIN-AA
Date & Time :09-Sep-202220:38:51
Instrument ID # LCM-0005
INTERNAL
090922-P-17 148 (2.461) Cm (146:149)
2: Scan ES+
8.75e5
487.28
488.31
595.47
596.48
461.12
459.20
489.32
149.06
279.13
381.34
407.41
556.59
593.39
644.66
703.11
718.47
819.06
831.69
883.78
948.63
998.27
090922-P-17 148 (2.467) Cm (148:149)
3: Scan ES-
2.59e4
116.87
279.29
325.40
280.27
253.26
241.09
199.72
117.75
134.94
367.47
395.33
465.29
481.62
556.50
569.31
606.30
m/z

8c

532

Sample Name :P-4
Test Name :NEW-JBL-3-MIN-AA
Date & Time :16-Sep-202219:22:30

Instrument ID # LCM-0005
INTERNAL

8d

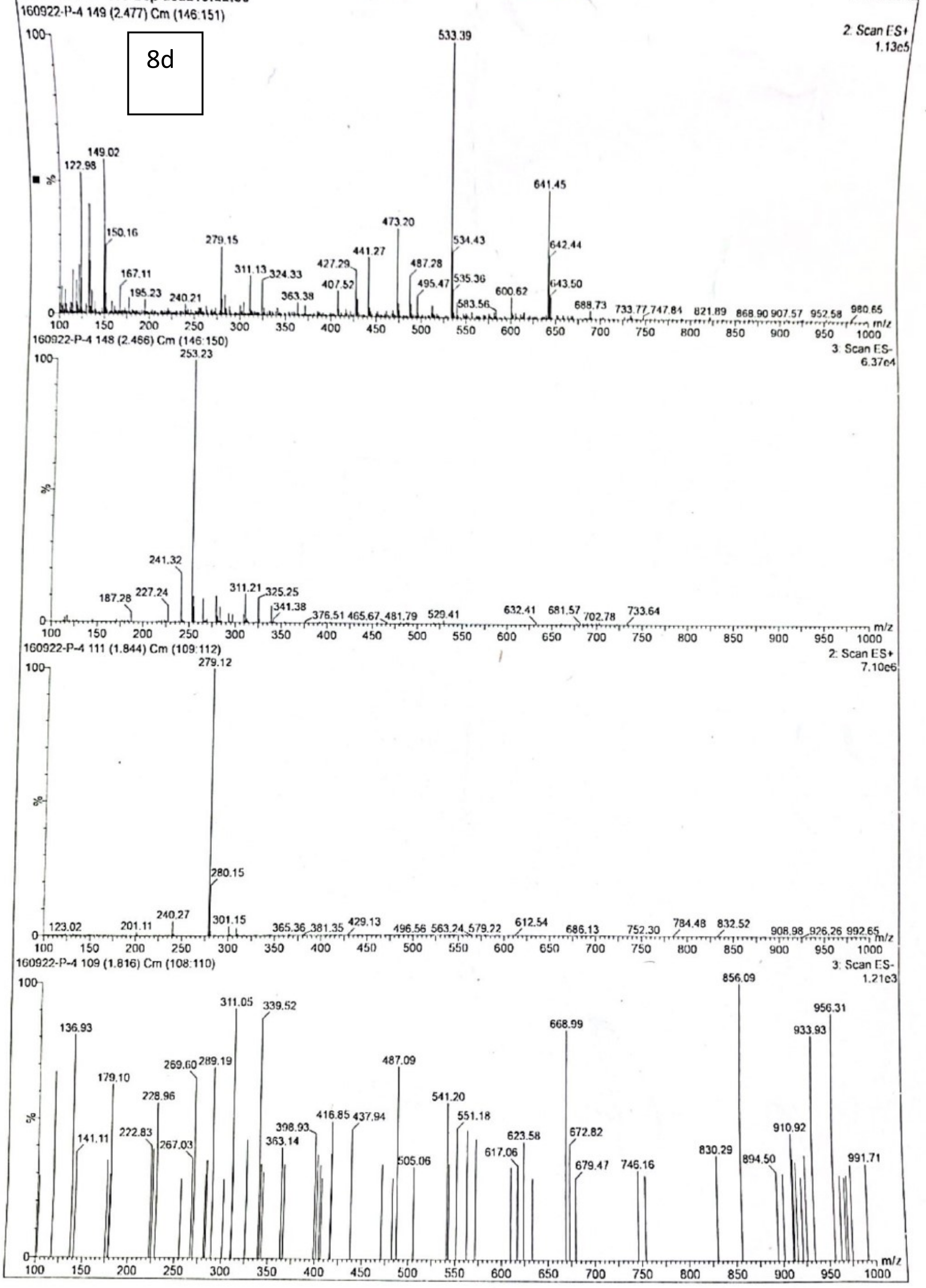

470.20

8e

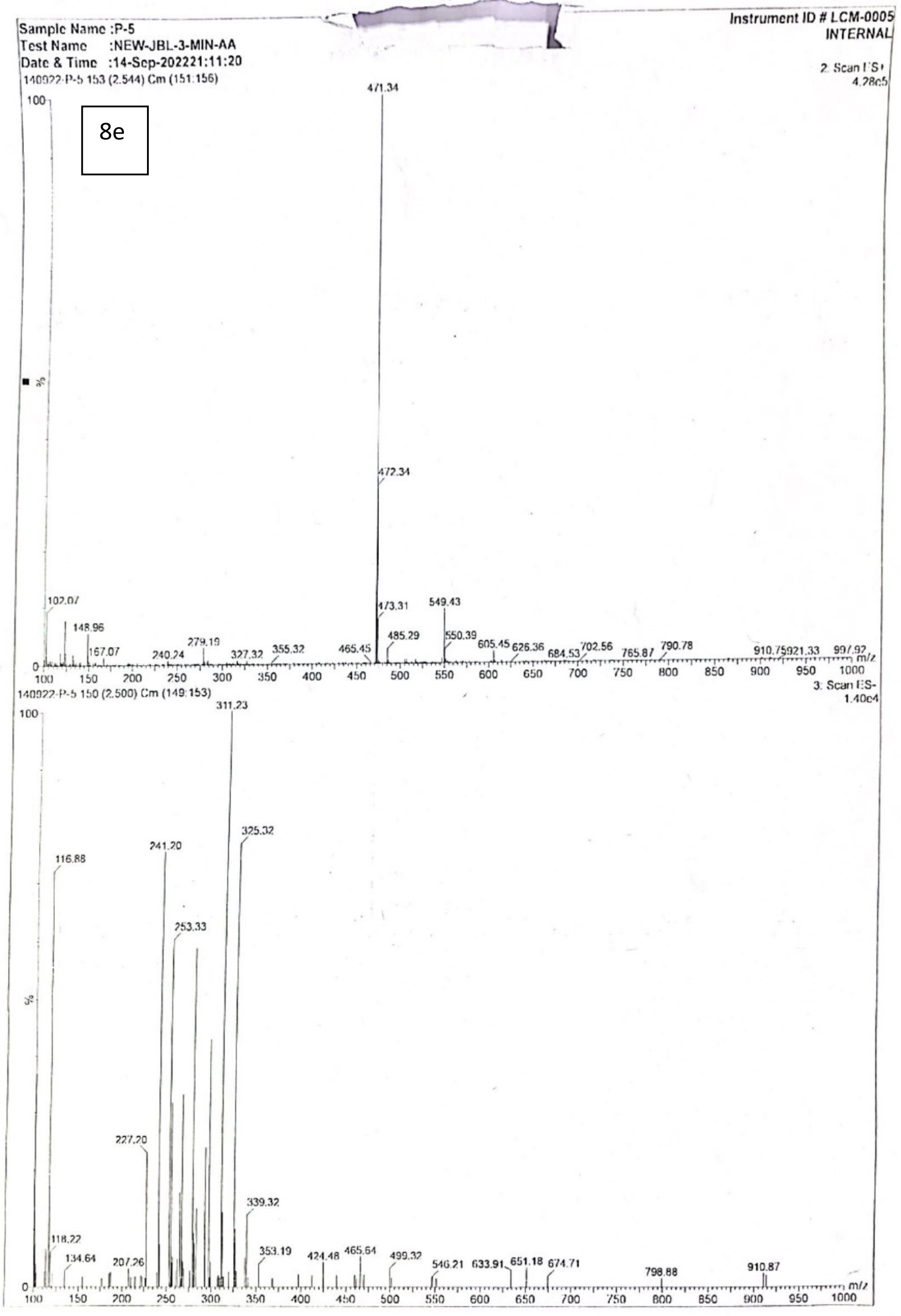

Sample Name :P-5
Test Name :NEW-JBL-3-MIN-AA
Date & Time :14-Sep-202221:11:20
Instrument ID # LCM-0005
INTERNAL
140922-P-5 153 (2.544) Cm (151:156)
2: Scan ES+
4.28e5
471.34
472.34
102.07
148.96
167.07
240.24
279.19
327.32
355.32
465.45
473.31
485.29
549.43
550.39
605.45
626.36
684.53
702.56
765.87
790.78
910.75
921.33
997.92
m/z
140922-P-5 150 (2.500) Cm (149:153)
3: Scan ES-
1.40e4
311.23
325.32
241.20
116.88
253.33
227.20
339.32
118.22
134.64
207.26
353.19
424.48
465.64
499.32
546.21
633.91
651.18
674.71
798.88
910.87
%
m/z

Sample Name :P-21
Test Name :NEW-JBL-3-MIN-AA
Date & Time :09-Sep-202220:50:27

Instrument ID # LCM-0005
INTERNAL

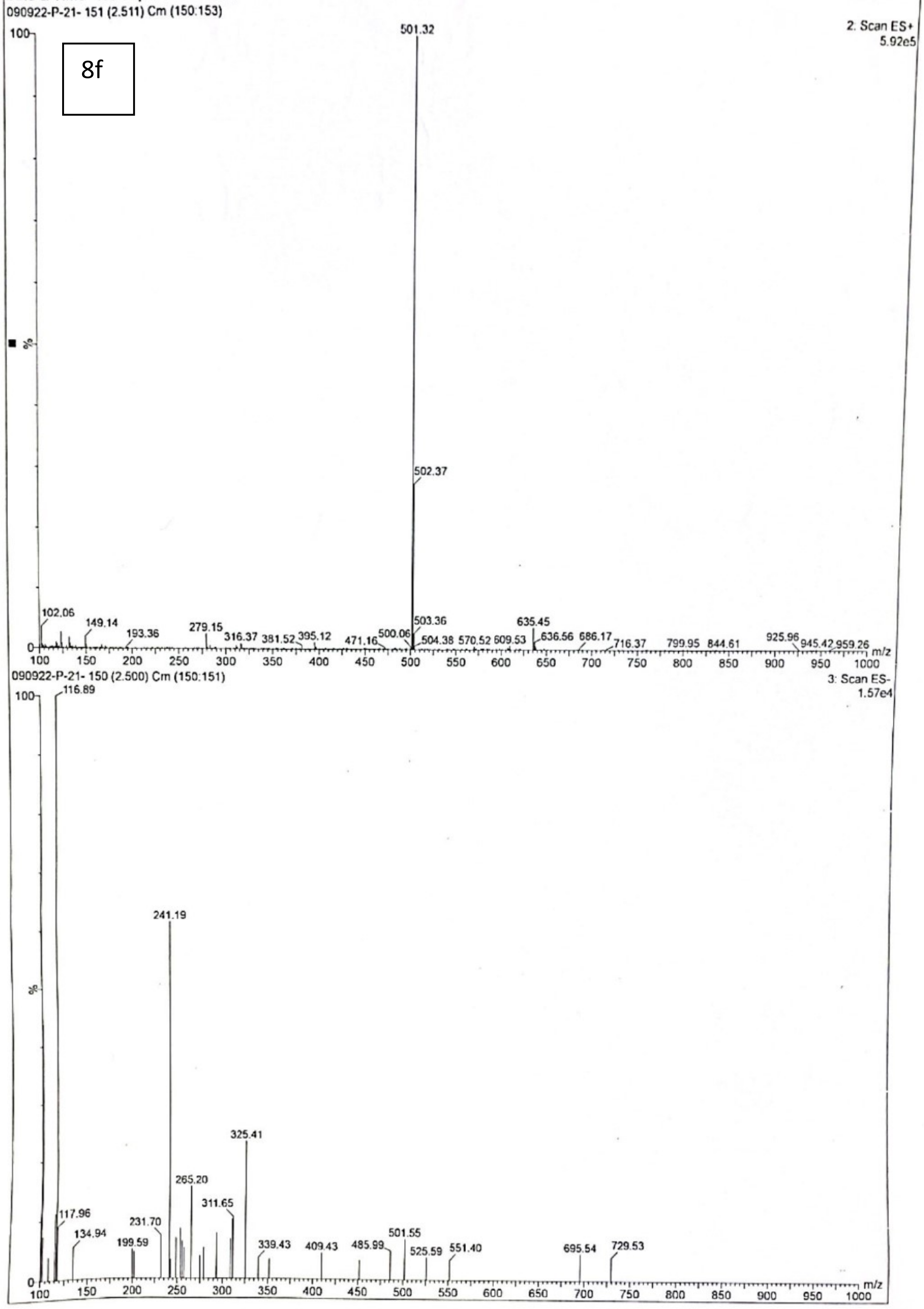

Sample Name :P-22
Test Name :NEW-JBL-3-MIN-AA
Date & Time :09-Sep-202222:30:12

Instrument ID # LCM-0005
INTERNAL

8g

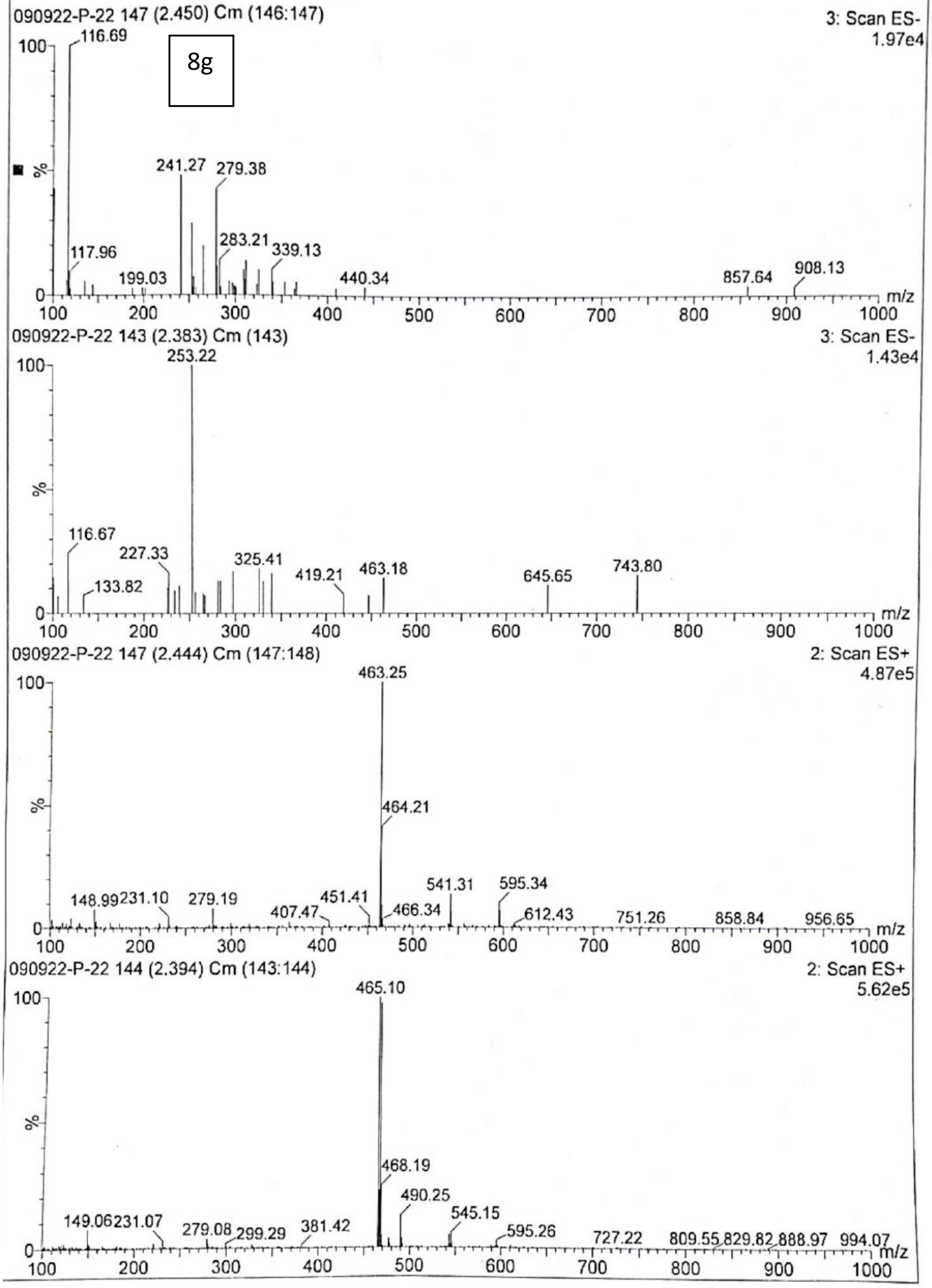

Sample Name :P-23
Test Name :NEW-JBL-3-MIN-AA
Date & Time :09-Sep-202220:46:41

[redacted]

Instrument ID # LCM-0005
INTERNAL

8h

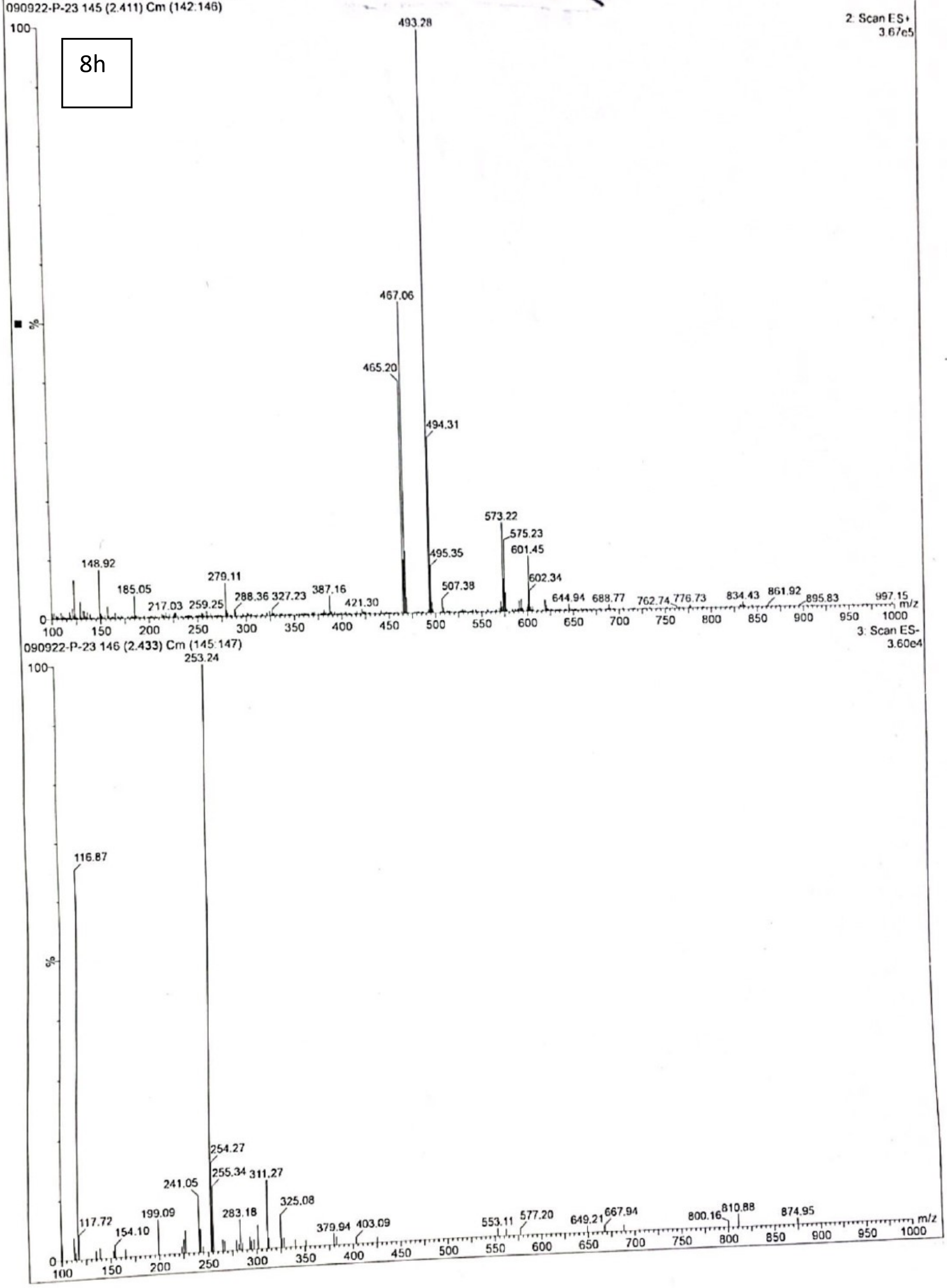

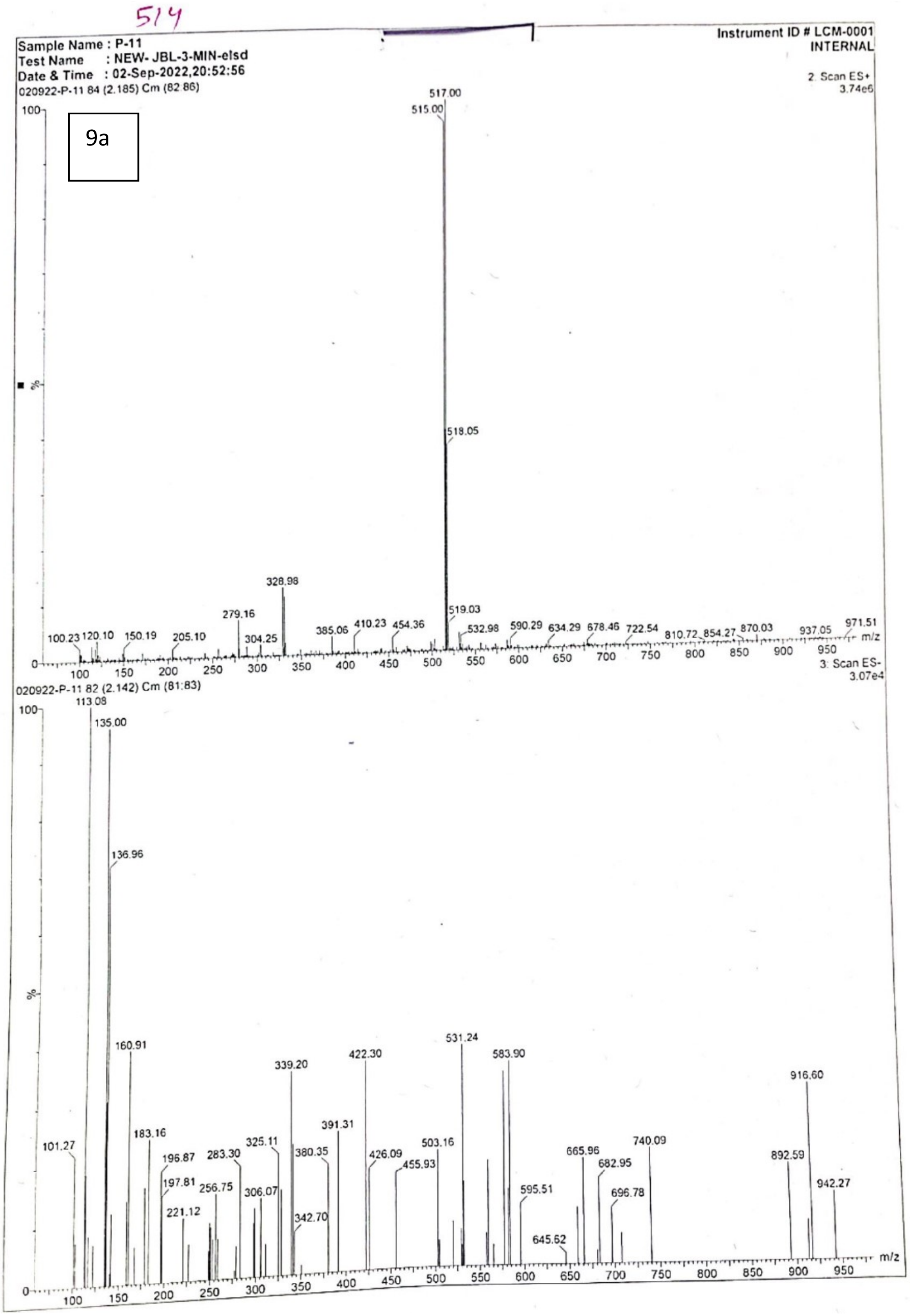
514
Sample Name : P-11
Test Name : NEW- JBL-3-MIN-elsd
Date & Time : 02-Sep-2022,20:52:56
Instrument ID # LCM-0001
INTERNAL
020922-P-11 84 (2.185) Cm (82:86)
2: Scan ES+
3.74e6
9a
517.00
515.00
518.05
519.03
328.98
279.16
304.25
100.23
120.10
150.19
205.10
385.06
410.23
454.36
532.98
590.29
634.29
678.46
722.54
810.72
854.27
870.03
937.05
971.51
m/z
%
020922-P-11 82 (2.142) Cm (81:83)
3: Scan ES-
3.07e4
113.08
135.00
136.96
160.91
183.16
101.27
196.87
197.81
221.12
256.75
283.30
306.07
325.11
339.20
342.70
380.35
391.31
422.30
426.09
455.93
503.16
531.24
583.90
595.51
645.62
665.96
682.95
696.78
740.09
892.59
916.60
942.27

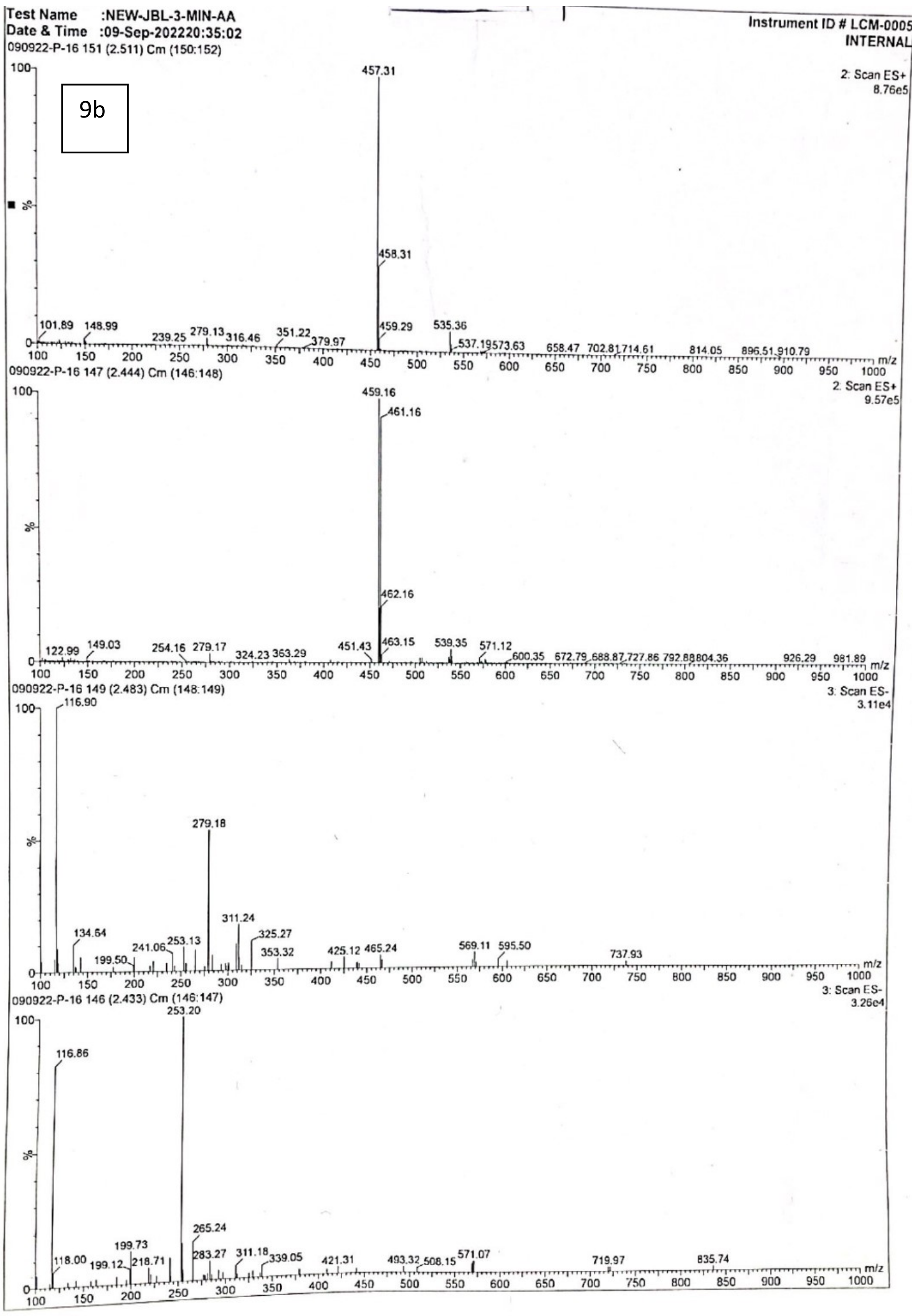
Test Name :NEW-JBL-3-MIN-AA
Date & Time :09-Sep-202220:35:02
Instrument ID # LCM-0005
INTERNAL
9b
090922-P-16 151 (2.511) Cm (150:152)
2: Scan ES+
8.76e5
457.31
458.31
459.29
535.36
090922-P-16 147 (2.444) Cm (146:148)
2: Scan ES+
9.57e5
459.16
461.16
462.16
463.15
090922-P-16 149 (2.483) Cm (148:149)
3: Scan ES-
3.11e4
116.90
279.18
311.24
325.27
090922-P-16 146 (2.433) Cm (146:147)
3: Scan ES-
3.26e4
253.20
116.86
265.24
m/z
%

528

Sample Name : P-9
Test Name : NEW- JBL-3-MIN-elsd
Date & Time : 02-Sep-2022,19:44:43

Instrument ID # LCM-0001
INTERNAL

9c

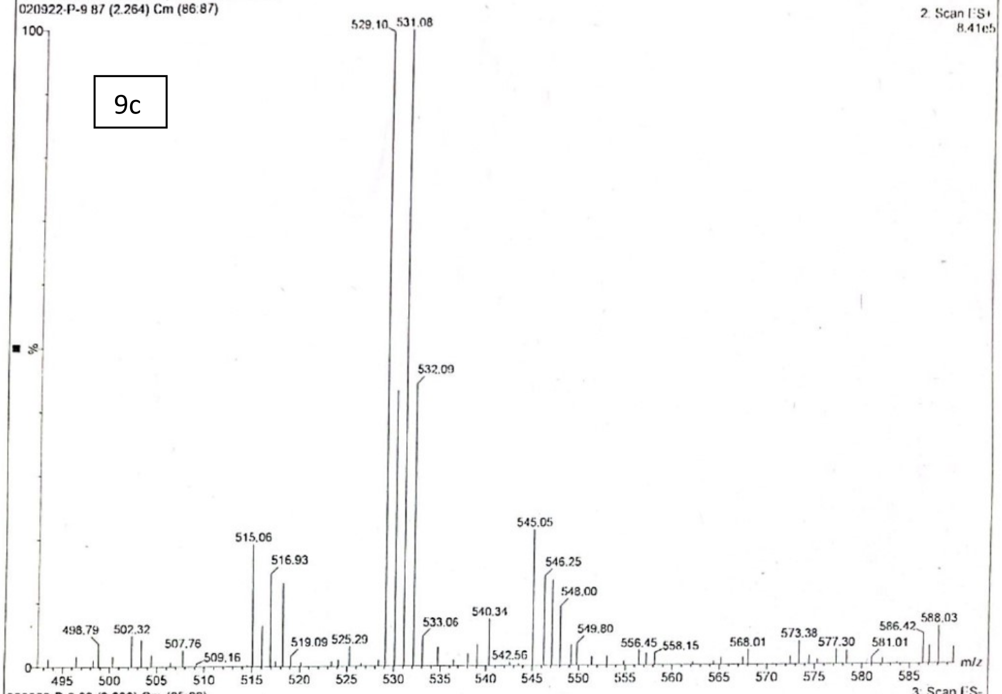

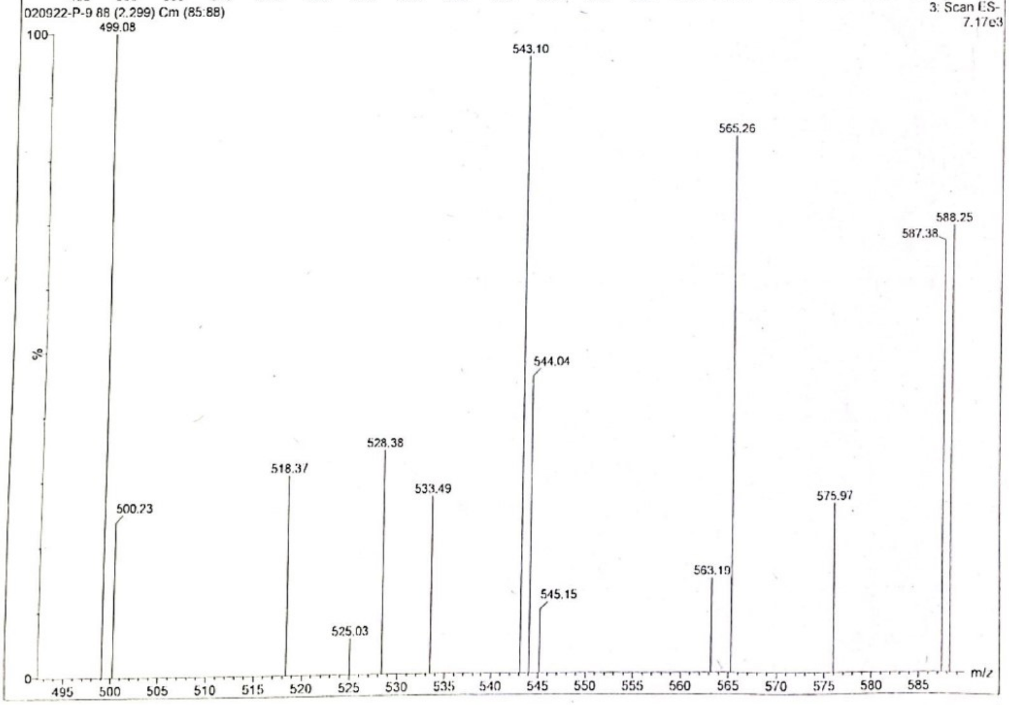

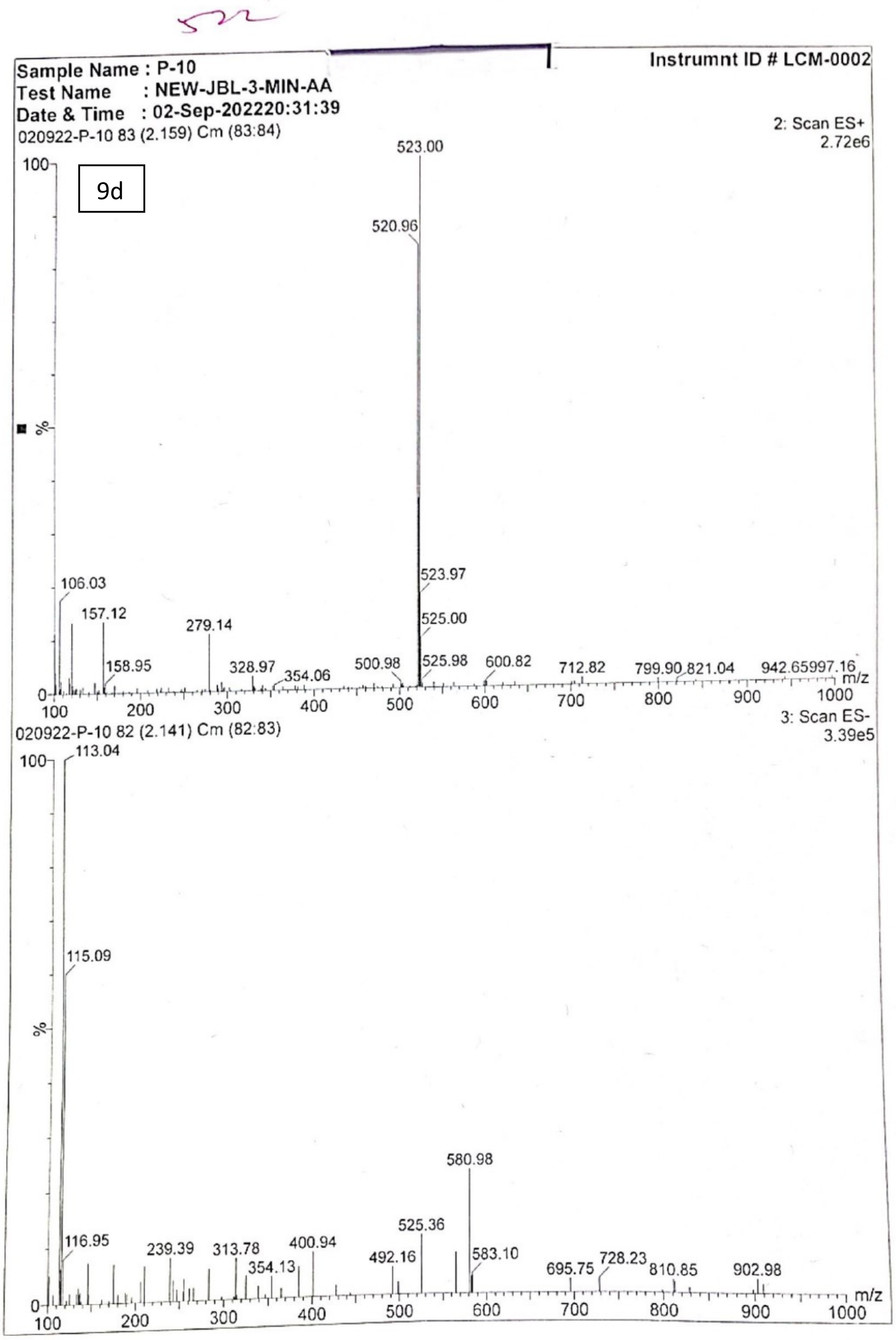

Figure S3. The mass spectra of **4**, **5a**, **5b**, **6a-6d**, **7a-7d**, **8a-8h** and **9a-9d** synthesised compounds.
